# Messenger RNAs of Yeast Virus-Like Elements Contain Non-templated 5′ Poly(A) Leaders, and Their Expression Is Independent of eIF4E and Pab1

**DOI:** 10.3389/fmicb.2019.02366

**Published:** 2019-10-30

**Authors:** Václav Vopálenský, Michal Sýkora, Tomáš Mašek, Martin Pospíšek

**Affiliations:** Laboratory of RNA Biochemistry, Department of Genetics and Microbiology, Faculty of Science, Charles University, Prague, Czechia

**Keywords:** virus-like element, linear cytoplasmic plasmid, pGKL, poly(A) leader, poxvirus, eIF4E, Pab1, Lsm1

## Abstract

We employed virus-like elements (VLEs) pGKL1,2 from *Kluyveromyces lactis* as a model to investigate the previously neglected transcriptome of the broader group of yeast cytoplasmic linear dsDNA VLEs. We performed 5′ and 3′ RACE analyses of all pGKL1,2 mRNAs and found them not 3′ polyadenylated and containing frequently uncapped 5′ poly(A) leaders that are not complementary to VLE genomic DNA. The degree of 5′ capping and/or 5′ mRNA polyadenylation is specific to each gene and is controlled by the corresponding promoter region. The expression of pGKL1,2 transcripts is independent of eIF4E and Pab1 and is enhanced in *lsm1*Δ and *pab1*Δ strains. We suggest a model of primitive pGKL1,2 gene expression regulation in which the degree of 5′ mRNA capping and 5′ non-template polyadenylation, together with the presence of negative regulators such as Pab1 and Lsm1, play important roles. Our data also support a hypothesis of a close relationship between yeast linear VLEs and poxviruses.

## Introduction

Yeast double-stranded DNA virus-like elements (VLEs; also termed linear cytoplasmic plasmids) have been found in the cytoplasm of yeast species from nine genera, including *Kluyveromyces*, *Debaryomyces*, *Saccharomyces*, *Saccharomycopsis*, *Wingea*, and *Pichia* (Fukuhara, 1995; Gunge and Tokunaga, 2004). The overall genetic organization of all yeast VLEs is identical to that of the most studied VLEs, pGKL1 (also termed K1) and pGKL2 (also termed K2), from the yeast *Kluyveromyces lactis*, where their presence is associated with the killer phenotype (Gunge et al., 1981). Killer strains contain 50–100 copies of each of the two linear VLEs per cell (Gunge, 1983). pGKL1 and pGKL2 VLEs have compact and extremely AT-rich genomes of sizes 8874 and 13,447 bp, respectively, and carry terminal inverted repeats with proteins covalently attached to their 5′ ends (Hishinuma et al., 1984; Stam et al., 1986; Tommasino et al., 1988).

**FIGURE 1:**
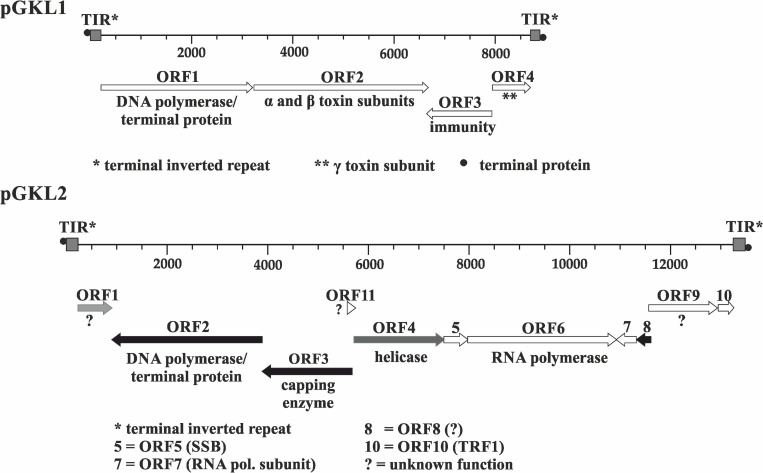
Genetic organization of pGKL VLEs (linear cytoplasmic plasmids). Known gene functions are indicated. Shades of gray indicate the extent of transcript capping as determined in this work, from black ORFs (>85% mRNAs 5′ capped) to white ORFs (<35% mRNAs 5′ capped). Transcripts from gray ORFs comprise 45–60% 5′ capped mRNAs and represent an intermediate degree of capping. All pGKL1 ORFs belong to the group with a low occurrence of 5′ capped transcripts. TIR, terminal inverted repeat; ^•^, terminal protein; SSB, single-stranded binding protein; TRF1, terminal recognition factor 1; ?, unknown protein function.

The smaller VLE, pGKL1, contains four open reading frames (ORFs), two of which (*K1ORF2* and *K1ORF4*) encode precursors of killer toxin subunits (Stark et al., 1984; Stark and Boyd, 1986; Tokunaga et al., 1989); *K1ORF3* is involved in an immunity phenotype in an unknown manner (Tokunaga et al., 1987), and *K1ORF1* codes for the pGKL1 VLE-specific DNA polymerase and a terminal protein (Fukuhara, 1987; Jung et al., 1987). Eleven ORFs have been reported in the larger pGKL2 VLE, which provides vital functions for pGKL1/pGKL2 maintenance in host cells (Schaffrath and Meacock, 1995). Some functions have been attributed to more than half of the proteins encoded by the pGKL2 VLE ORFs. *K2ORF2* codes for a pGKL2-specific DNA polymerase and a terminal protein (Tommasino et al., 1988; Takeda et al., 1996). *K2ORF3* codes for a virus-like mRNA capping enzyme (Larsen et al., 1998; Tiggemann et al., 2001). *K2ORF4* encodes a putative helicase, which is involved in the transcription of VLE-specific mRNAs (Stark et al., 1990; Sýkora et al., 2018), *K2ORF5* codes for a single-stranded DNA-binding protein, and *K2ORF10* encodes a protein bound to the terminal inverted repeats of both pGKL VLEs. All these proteins, with the exception of K2Orf3p and K2Orf4p, are probably involved in pGKL1/2 replication (Stark et al., 1990; McNeel and Tamanoi, 1991; Tommasino, 1991; Schaffrath and Meacock, 2001; Jeske et al., 2007). *K2ORF6* and *K2ORF7* code for putative subunits of a pGKL1/2-specific RNA polymerase (Wilson and Meacock, 1988; Schaffrath et al., 1997). We showed in our parallel study that K2Orf6p, K2Orf7p, and K2Orf3p interact and form a pGKL transcription machinery core complex, which loosely interacts with the putative helicase K2Orf4p (Sýkora et al., 2018). No function has been assigned to the four remaining ORFs (*K2ORF1, 8, 9*, and *11*) (Figure 1). All ORFs are likely expressed independently. Each gene is preceded by an upstream conserved sequence (UCS) motif, which is located approximately −30 nucleotides from the putative start codon for pGKL1 UCSs (AT^A^/_C_TGA) or up to −100 nucleotides in the case of pGKL2 UCSs (ATNTGA) (for review, see Stark et al., 1990; Gunge and Tokunaga, 2004). Sequences located between AUG initiation codons and their respective UCS (inclusive) are called UCRs (upstream control regions) and act as promoters in *sensu lato* (Meinhardt et al., 1994; Schrunder and Meinhardt, 1995; Schrunder et al., 1996).

In summary, yeast linear pGKL VLEs represent a model and the most studied example of a unique population of yeast cytoplasmically localized linear double-stranded DNA VLEs. They code for their own replication, transcription, and an RNA-modification apparatus, making them remarkably independent of their host cells and evoking thoughts about their similarity to other cytoplasmically localized linear DNA genomes such as viruses belonging to the families *Poxviridae* and *Asfarviridae* (Jeske et al., 2007). In our parallel study, we analyzed a transcription apparatus of pGKL VLEs, demonstrating that the pGKL RNA polymerase K2Orf6p/K2Orf7p is related to viral RNA polymerases from the *Poxviridae* and *Iridoviridae* families and that an extended pGKL UCS motif shows high similarity to the UCE motif of the vaccinia virus early promoters (Sýkora et al., 2018).

The *K. lactis* heterotrimeric killer toxin mechanism of action is well understood. A γ-subunit of the toxin inhibits the growth of sensitive yeast cells by cleaving their tRNAs on the 3′ side of the modified wobble nucleoside 5-methoxycarbonylmethyl-2-thiouridine (mcm5s2U), which leads to cell cycle arrest in the G1 phase (Butler et al., 1991; Huang et al., 2005; Lu et al., 2005; Jablonowski et al., 2006; Meineke et al., 2012). Readers are referred to work done by Meineke et al. (2012) and Kast et al. (2014) who provide more information concerning the action of the *K. lactis* toxin and related ribotoxins.

In this work, we focus on the uncharacterized transcriptome of yeast cytoplasmic linear dsDNA VLEs and investigate the expression of VLE-specific mRNAs. We analyzed transcripts from all pGKL ORFs using the rapid amplification of 5′ cDNA ends (5′ RACE) method, revealing that mRNAs transcribed from pGKL VLEs contain poly(A) leaders of variable lengths at their 5′ ends that are not complementary to the VLE genomic DNA sequence and thus must be synthesized in a template-independent manner. We demonstrate that VLE promoters directly determine the formation and length of the mRNA 5′ end leaders and their capping, suggesting a possible role of the VLE RNA polymerase in this unusual phenomenon. We also show that pGKL mRNAs are not 3′ polyadenylated and that most even lack the m^7^G cap at their 5′ ends. These findings directed our interest to the translation of pGKL mRNAs. Using *in vitro* and *in vivo* approaches, we demonstrate that the expression of some VLE genes is independent of the major cellular cap-binding protein, translation initiation factor 4E (eIF4E), the poly(A)-binding protein 1 (Pab1), and the Sm-like protein Lsm1. The latter two proteins were previously speculated to be possible translational enhancers and/or stabilizers of cellular and viral mRNAs bearing 5′ poly(A) stretches (Bergman et al., 2007; Gilbert et al., 2007). Thus, our results suggest the existence of an unusual translation initiation mechanism.

## Materials and Methods

### Modification of pGKL VLEs Using Homologous Recombination *in viv*o

In general, *K. lactis* IFO1267 cells were transformed with a PCR-generated fragment (for an example of such a construct, see Supplementary Figure S5) consisting of 5′ and 3′ ends homologous to the part of the pGKL VLE to be modified, a non-homologous part that can be used to introduce mutation/s into a VLE-specific protein coding sequence, and a gene encoding a resistance marker (typically against G418 or hygromycin B) whose transcription was driven by the UCR of *K1ORF1* or *K1ORF2*. This type of construct was prepared by fusion PCR as follows. The first primary PCR product was amplified from a native pGKL2 VLE using Pfu DNA polymerase (Fermentas) and the primers ORF3_SAM_del_F1 and ORF3_SAMdel_R1. The second primary PCR product was amplified using Pfu DNA polymerase (Fermentas) and the primers KL_orf6C_Flag2F and ORF3_SAMdel_R2 (see Supplementary Table S1) from a modified pGKL1 VLE whose *K1ORF2* coding sequence was previously replaced with the *G418* resistance gene. Both primary PCR products, which had defined overlapping ends, were synthesized as follows: 5 min at 95°C; 30 cycles of 30 s at 94°C, 30 s at 56.2°C, and 90 s at 72°C; and finally, 10 min at 72°C. Then, 1 μl of each PCR mixture was used without purification for a second PCR using Pfu DNA polymerase (Fermentas), the primers ORF3_SAM_del_F1 and ORF3_SAMdel_R2, and the following conditions: 5 min at 95°C; 35 cycles of 30 s at 94°C, 30 s at 60.1°C, and 2 min at 72°C; and finally, 10 min at 72°C. The resulting fusion PCR product was purified by agarose gel electrophoresis and transformed into *K. lactis* IFO1267. After 5 h of cultivation under non-selective conditions, the cells were plated onto solid medium containing G418 (250 μg/ml). The DNA content of selected clones was analyzed for the presence of the modified pGKL VLE using agarose gel electrophoresis. Both modified and wild-type target VLEs were detected within a single clone after transformation. Colonies containing both the modified and wild-type target VLEs were selected and cultivated under selective conditions for approximately 60 generations, and their DNA contents were analyzed using agarose gel electrophoresis. For subsequent analysis, only colonies containing a modified version of the target VLE were used. Site-specific integration of the PCR cassette into a pGKL2 VLE was evaluated by PCR with primers (in_Kan_rev1 and in_ORF3_forw) followed by sequencing of the gel-purified PCR fragment.

### RNA Purification, Electrophoresis, Reverse Transcription, and 5′ and 3′ RACE

Total yeast RNA was purified by the hot acidic phenol procedure (Lin et al., 1996). The remaining DNA was removed using a DNA-free Kit (Ambion) according to the manufacturer’s protocol. The quality of RNA was assessed by electrophoresis according to the protocol by Masek et al. (2005). For 5′ RACE, RT-PCR was carried out as follows: 0.5 μg of total yeast RNA and 0.15 μg of random primers (Invitrogen) were used for cDNA synthesis using 100 U of SSC III Reverse Transcriptase (Invitrogen) in a 20-μl reaction (25°C for 10 min, 50°C for 99 min, and 70°C for 15 min). After cDNA purification using the High Pure PCR Product Purification Kit (Roche), 800 U of recombinant TdT (Roche) and 0.5 mM dGTP (Fermentas) were used for cDNA tailing in 1 × TdT buffer without CoCl_2_ for 30 min at 37°C with subsequent TdT inactivation at 70°C for 10 min. For amplification of cDNA, 2.5 μl of the reaction mixture was used for the following PCR with the universal olig2(dC) anchor primer and an appropriate gene-specific primer. In the case of 3′ RACE, 0.5 μg of total yeast RNA was polycytidinylated using a Poly(A) Tailing Kit (Applied Biosystems) with 2 mM CTP in a total volume of 25 μl for 90 min at 37°C. Reverse transcription was then performed using the oligo(dG)anch2 primer as indicated above. After cDNA purification using the High Pure PCR Product Purification Kit (Roche), 2.5 μl of the purified cDNA was used for a subsequent PCR with the universal primer anch2 and an appropriate gene-specific primer. In both types of RACE experiments, following PCR amplification and electrophoresis, the corresponding fragments were purified from the gel using a FastBack DNA Minispin Kit (Renogen Biolab), cloned into a pCR4-TOPO plasmid using the TOPO strategy and sequenced using the universal T7 promoter primer and/or a T3 primer. All primers used in this study are listed in Supplementary Table S1.

### eIF4E Expression and Purification

The pGEX4T2::CDC33 plasmid was transformed into the expression strain *Escherichia coli* BL21(DE3) (Merck). A 500-ml culture was grown at 28°C in 2xTY medium supplemented with ampicillin (100 μg/ml) until the OD_600_ reached 0.20. At this point, the culture was cooled to 15°C, cultivated for 4 h, and then induced by 0.1 mM isopropyl β-D-thiogalactopyranoside. Production of recombinant protein was carried out with shaking at 15°C for another 14–16 h. Lysates were prepared using the B-PER Protein Extraction Reagent (Thermo Scientific) according to the manufacturer’s instructions and stored at −70°C for further use.

### mRNA/eIF4E Binding Assay

On the day of the binding experiment, bacterial lysate containing the GST-eIF4E fusion protein was thawed in an ice bath and centrifuged for 10 min at 15,000 *g* (4°C); GST-eIF4E was then purified using GST-affinity chromatography in a batch setup. In brief, 1 ml of the lysate was incubated for 80 min with 100 μl of the glutathione-Sepharose 4 Fast Flow resin (GE Healthcare) at 4°C and washed four times with at least 40 volumes of ice-cold 1× phosphate-buffered saline (PBS). After the last wash step, the GST-eIF4E fusion protein bound to glutathione-Sepharose was resuspended in 200 μl of buffer I (20 mM HEPES, pH 7.5; 0.1 mM EDTA, pH 8.0; 100 mM KCl; and 1 mM β-mercaptoethanol) and mixed with 3 μg of DNase I-treated total RNA purified from *K. lactis* IFO1267. Next, 2 μl of the reaction mixture was removed for subsequent RT-PCR and real-time quantitative PCR (qRT-PCR) analyses. The rest of the mixture was incubated for 2 h at room temperature and washed six times (approximately 70 volumes in each wash step) with buffer I. After the last wash step, the GST-eIF4E-glutathione-Sepharose slurry containing bound mRNA was subjected to RT-PCR and detection of specific mRNAs. All buffers used for protein purification contained Complete (EDTA-free) Protease Inhibitor (Roche), and all buffers used for handling RNA contained RNasin Ribonuclease Inhibitor (Promega).

Two microliters of the reverse transcriptase reaction mixture (obtained from the eIF4E-mRNA *in vitro* binding assay) was subjected to PCR amplification (5 min at 94°C; 35 cycles of 30 s at 94°C; 30 s at 50°C; 45 s at 72°C; and finally, 4 min at 72°C) using Taq DNA polymerase (Roche) and *HGT1* and *K2ORF5* gene-specific primers (listed in Supplementary Table S1).

As a control, total RNA used for the experiment was reverse transcribed and subjected to real-time PCR amplification (Supplementary Figure S3).

### Transfer of Cytoplasmic VLEs to Yeast Strains by Incomplete Mating

The *Saccharomyces cerevisiae* strain YAT547 (*kar1*) containing pGKL VLEs (Gunge and Yamane, 1984) and isogenic *S. cerevisiae* ρ^0^ strains bearing the wild-type *CDC33* gene (CWO4ρ^0^*CDC33*wt) and its temperature-sensitive mutations (CWO4ρ^0^*cdc33-1* and CWO4ρ^0^*cdc33-42*, for exact genotypes see Supplementary Table S2) were cultured separately in YPD medium at 24°C overnight. Cells were pelleted the next day and resuspended in 200 μl of water, and 5 μl from each of the two required strains (approximately 3 × 10^6^ cells) was mixed on the YPD agar plate and further incubated for 5 h at 24°C. The cells were then scraped into liquid YPD medium, incubated at 24°C with gentle shaking overnight and plated on selective plates. The resulting strains were tested for auxotrophic markers, mating type and killer toxin production under both permissive and non-permissive conditions. Strains displaying a temperature-sensitive phenotype (when applicable), killer toxin production, growth on SD medium lacking methionine, no visible growth on SD medium lacking uracil, and a MATa mating type were used for subsequent analyses.

### pGKL VLE Purification and Electrophoresis

For the analysis of pGKL VLEs, a protocol based on that of Pospisek and Palkova (1991) was used. Briefly, cells were grown for 3 days on a dish containing the appropriate antibiotics, transferred into a microplate well, and dried for 2 h at 45°C. After complete drying, the cells were resuspended in 40 μl of freshly prepared TESP buffer (20 mM Tris–Cl, pH 8; 50 mM EDTA-NaOH, pH 8; 2% SDS; and 0.5 mg/ml pronase E) and dried at 37°C overnight. The sample was completely resuspended the next day in 40 μl of 1 × DNA loading buffer (Fermentas). Then, 15 μl of the sample was analyzed using agarose electrophoresis (0.5% agarose in TAE buffer, 1 V/cm) for at least 20 h. After electrophoresis, the gel was incubated in a solution containing ethidium bromide (0.5 μg/ml) and RNase A (50 μg/ml) for 3 h.

### Removal of Mitochondrial DNA Using Ethidium Bromide Treatment

Isogenic *S. cerevisiae* strains bearing the wild-type *CDC33* gene (CWO4*CDC33*wt) and its temperature-sensitive mutations (CWO4*cdc33-1* and CWO4*cdc33-42*; for exact genotypes, see Supplementary Table S2) (Altmann et al., 1989; Altmann and Trachsel, 1989) were cultivated for 2 days in synthetic dropout minimal medium (SD) that lacked leucine and tryptophan (SD-TL) and contained ethidium bromide at a final concentration of 25 μg/ml. Afterward, these cultures were diluted 5000 times, cultivated again for 2 days, and then diluted and cultivated once more. Loss of mitochondria was verified by the lack of colony growth on medium with glycerol as the sole carbon source and by DAPI staining followed by fluorescence microscopy.

### Plasmid Construction for Killer Toxin Production in CWO4 Yeast Strains

The *CDC33* (eIF4E) gene was amplified from *S. cerevisiae* genomic DNA using Pfu DNA polymerase (Fermentas) with eIF4Ef and eIF4Er primers containing *Nco*I and *Hin*dIII restriction sites as follows: 5 min at 95°C; 25 cycles of 30 s at 94°C, 30 s at 55°C, and 2 min at 72°C; and finally, 10 min at 72°C. The PCR cassette was digested and inserted into the *Nco*I and *Hin*dIII restriction sites of pGEX-4T2 (GE Healthcare) to generate a pGEX4T2::CDC33 construct. A pYX212 yeast shuttle plasmid (Ingenius) was digested with *Eco*RI and *Xho*I restriction endonucleases. A sequence encoding the K1 toxin was excised from a pYX213::M1 (Valis et al., 2006) plasmid using the same restriction enzymes and ligated into the digested pYX212 plasmid to generate the plasmid pYX212::M1. All clones were verified by restriction endonuclease digestion and sequencing. All primers and plasmids used in this study are listed in Supplementary Tables S1, S3, respectively.

### Transformation of Yeast Cells

All yeast transformations with plasmid DNA were performed with the one-step LiCl method (Gietz and Woods, 2002). The cells were grown in a shaker at 28°C in SD minimal medium lacking selected amino acids or nucleotide bases to ensure plasmid maintenance. Transformation using a PCR fragment for homologous recombination was carried out in the same way with the exception of a 5-h incubation under non-selective conditions immediately after transformation but prior to plating.

### Killer Toxin Production in Modified CWO4 Yeast Strains

Tested strains (50 ml) were cultured in an appropriate medium at 24°C with shaking. When the *A*_600_ reached 0.9, the cells were harvested and washed three times with the same medium preheated to 24°C. One-half of the culture (25 ml) was mixed with 25 ml of fresh medium preheated to 24°C and then cultivated at 24°C for more than 20 h. The second half of the tested culture (25 ml) was mixed with 25 ml of fresh medium preheated to 52°C and then cultivated at 37°C for more than 20 h. Aliquots (2 ml) were taken at 0, 3, 6, and 12 h. Samples were harvested, sterilized using 0.2-μm filters, and assayed for killer toxin activity in the culture medium by an agar well diffusion assay using *S. cerevisiae* S6/1 as a sensitive strain.

### Assay of Killer Toxin Activity

Filter-sterilized culture medium was tested for killer toxin activity by the agar well diffusion assay using *S. cerevisiae* S6/1 as a sensitive strain. Approximately 2 × 10^5^ sensitive yeast cells were plated onto OSS1 agar plates (5.3% worth agar; 0.7% agar; 1% glucose; 1 M sorbitol; 0.002% methylene blue; buffered to pH 4.7 with sodium citrate) for testing M1 killer toxin activity or onto YPD plates (1% yeast extract; 2% peptone; 2% glucose; 2% agar) for testing pGKL1 killer toxin activity. Wells were made with an 8-mm-diameter cork borer, and 100 μl of filter-sterilized culture medium or 100 μl of serially diluted filter-sterilized culture medium was pipetted into the well. For quantitative results, inhibition zones were measured using a calibrated digital microscope after 48 h of incubation at 24°C.

### *PBP1*, *PAB1*, and *LSM1* Gene Deletions

The *PBP1*, *PAB1*, and *LSM1* genes were deleted from the chromosomes of the *K. lactis* IFO1267 strain using a loxP-*G418*-loxP cassette (Guldener et al., 1996). Briefly, the cassette for deletion of the *PBP1* gene was amplified from the pUG6 plasmid by PCR with the primers KL_pbp1-del_Rev and KL_pbp1-del_For. The PCR product was resolved by agarose electrophoresis, purified, and transformed into *K. lactis* IFO1267. Deletion of the *PBP1* gene was verified by PCR. This modified strain was transformed with the pSH65 plasmid (Gueldener et al., 2002), incubated for 5 h in non-selective medium, and plated on solid medium supplemented with phleomycin (400 μg/ml). Monocolonies were grown on YPD plates with phleomycin for 2 days and subsequently tested for their ability to grow on G418 (250 μg/ml) selective plates. The excision of the *G418* cassette was verified by PCR for selected colonies that did not grow in the presence of G418. These colonies were cultivated for 5 days with daily dilution under non-selective conditions (YPD medium only), resulting in loss of the Cre-containing pSH65 plasmid. The resulting yeast strain, *K. lactis* IFO1267*pbp1*Δ, was used for subsequent *PAB1* deletion using the primers KL_pab1-del_For and KL_pab1-del_Rev in a similar fashion with the exception of the *G418* cassette excision. *K. lactis* IFO1267 was used for *LSM1* deletion using primers KL_lsm1-del_For and KL_lsm1-del_Rev in a similar fashion with the exception of *G418* cassette excision. The PCRs were carried out similarly (5 min at 95°C; 30 cycles of 30 s at 94°C, 30 s at 55°C, and 4 min at 68°C; and finally, 10 min at 68°C). The nucleotide sequences of the primers used for verification of the gene disruption cassettes are summarized in Supplementary Table S1.

### Killer Toxin Production in *K. lactis* Strains With Deletion of the *PBP1*, *PAB1*, and *LSM1* Genes

Test cultures (10 ml) were incubated overnight in YPD medium supplemented with G418 (250 μg/ml) at 28°C with shaking. The next day, the optical density of the tested strains was measured, and the strains were inoculated at an initial OD_600_ of 0.035 into 50 ml of fresh medium preheated to 28°C and then cultivated at 28°C for approximately 80 h. Aliquots (2 ml) were taken during the experiment. Cells were harvested using centrifugation, and the supernatant was sterilized using 0.2-μm filters. After serial twofold dilution, the supernatant samples were assayed for killer toxin activity by a standard agar well diffusion assay using *S. cerevisiae* S6/1 as a sensitive strain and YPD agar plates (Supplementary Figure S4A). The width of the inhibition zone was independently measured at >20 positions within a single zone using a calibrated digital microscope (Supplementary Figure S4C), and the mean width of the inhibition zone normalized to the concentration of production cells *(I)* was calculated (Supplementary Figure S4B).

### Strains, Plasmids, VLEs, Primers, and Statistical Analysis

Please see the Supplementary Material for additional information concerning the statistical analyses, strains, primers, plasmids, and VLEs used.

## Results and Discussion

### VLE Transcripts Are Uncapped and Contain a Non-templated 5′ Poly(A) Leader

We used a modified 5′ RACE method to analyze the 5′ ends of the pGKL transcripts. SuperScript III reverse transcriptase, which was used for cDNA synthesis for 5′ RACE mapping, can overcome the 5′–5′ triphosphate bond between the 5′ m^7^G cap and the first nucleotide of the nascent mRNA transcript and insert a cytosine nucleotide into the cDNA position, which is complementary to the 5′ guanosine cap. This feature of the reverse transcriptase enables determination of whether the mRNA is capped or not (Davison and Moss, 1989a; Schwer et al., 1998; Schmidt and Mueller, 1999). PCR fragments obtained by 5′ RACE were verified by digestion with restriction endonucleases and cloned into a pCR4-TOPO plasmid. Twenty to seventy-nine independent clones corresponding to the 5′ mRNA regions were sequenced and analyzed for each ORF. We used the *K. lactis ACT* transcript (EnsemblFungi Id: KLLA0_D05357g), which codes for actin and is transcribed by RNA polymerase II from a nuclear gene, as a control for 5′ m^7^G capped mRNA.

5′ RACE analysis of the pGKL transcripts revealed a unique composition of their 5′ ends. With the exception of some viral transcripts, eukaryotic mRNAs generally contain an m^7^G cap structure at their 5′ ends, which is a prerequisite for their stability and efficient translation initiation (Ross, 1995; Mukherjee et al., 2012; Ramanathan et al., 2016). Although pGKL VLEs encode their own capping enzyme (K2Orf3p), we found only three ORFs (*K2ORF2*, *K2ORF3*, and *K2ORF8*) of the 15 encoded by pGKL VLEs for which at least 86% of the transcripts were 5′ capped. mRNAs encoded by other pGKL1/2 ORFs were 5′ capped less frequently (Supplementary Table S4). We did not detect any capped transcripts for *K2ORF10* and found only 4–8% capped mRNAs among the *K1ORF2*, *K1ORF3*, *K2ORF7*, and *K2ORF11* transcripts.

Interestingly, *K2ORF10* and *K2ORF7* have been previously shown to be essential for pGKL1/2 VLE maintenance in yeast cells (Schaffrath et al., 1997, 1999). The indispensability of *K2ORF11* for replication of pGKL VLEs has not yet been assessed. *K2ORF10* belongs to the highly expressed pGKL genes (McNeel and Tamanoi, 1991; Schaffrath et al., 1999). *K1ORF2* and *K1ORF3* code for the relatively highly secreted *K. lactis* killer toxin and a corresponding immunity phenotype, respectively (Stark and Boyd, 1986; Tokunaga et al., 1989). The absence and/or low occurrence of the guanosine cap at the 5′ ends of some pGKL mRNAs thus apparently does not substantially affect the expression of the corresponding genes.

**FIGURE 2:**
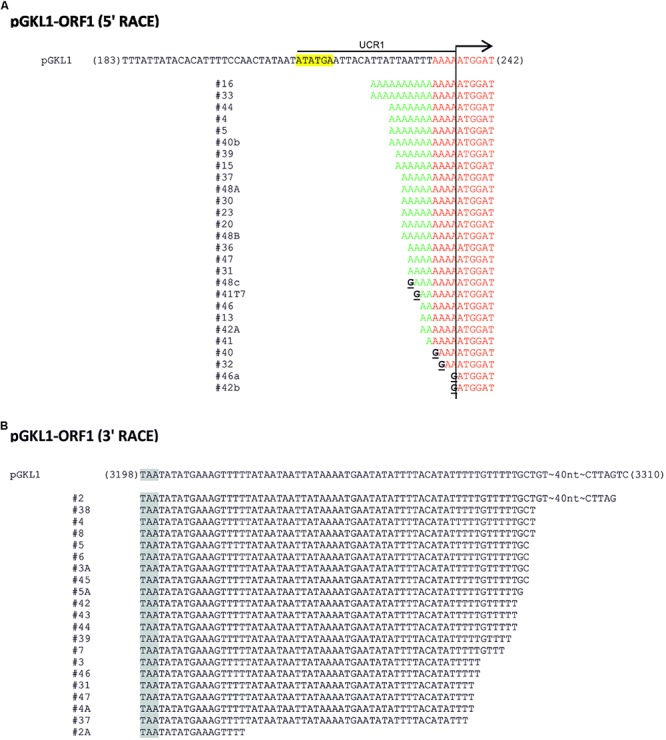
5′/3′ RACE analysis of pGKL transcripts. **(A,B)** Representative pictures of 5′ RACE **(A)** and 3′ RACE **(B)** analyses of individual mRNAs transcribed from *K1ORF1* encoded by the pGKL1 VLE. The top sequence corresponds to the template (VLE genomic) DNA; the cDNA sequences below represent individual clones obtained by the reverse transcription of individual mRNA molecules. In the case of 5′ RACE **(A)**, sequences homologous to the template (VLE genomic) DNA are highlighted in red, and non-templated 5′ leaders of the mRNA transcripts are in green. Guanosine nucleotides uncovered as cytosines during the first strand cDNA synthesis and corresponding to the 5′ mRNA caps are depicted in black and underlined. *K1UCR1*, which was also used as a promoter sequence in other experiments, is underlined. *K1UCS1* is highlighted in yellow. **(B)** Sequences obtained by 3′ RACE. Stop codons are highlighted in gray. For details concerning transcripts of other pGKL ORFs, please refer to Supplementary Tables S5A,C and the description therein.

The occurrence of 5′ capping in transcripts from other pGKL1/2 ORFs fell between these two extremes within the interval of 17–55% of capped mRNAs (Supplementary Tables S4, S5). Thereto, all pGKL1/2 genes gave rise to transcripts containing a short leader of non-templated adenosine nucleotides (1–21) at their 5′ ends that could not have been continuously transcribed from the VLE genomes (Figure 2A and Supplementary Tables S4, S5). In contrast, *K. lactis* actin mRNA revealed 5′ guanosine caps and no 5′ poly(A) leaders in 100% of the analyzed mRNA sequences (Supplementary Tables S4, S5B). The presence of a 5′ guanosine cap in *K2ORF8* mRNAs was also confirmed by the oligo-capping method (Maruyama and Sugano, 1994) (Supplementary Figure S1).

**FIGURE 3:**
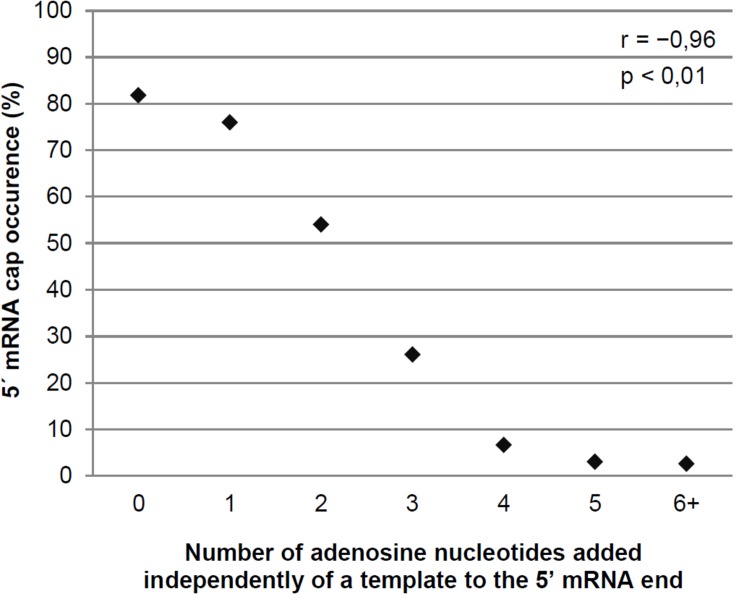
The degree of non-templated 5′ mRNA polyadenylation negatively correlates with the proportion of 5′ mRNA caps in pGKL transcripts. Transcripts were divided into individual groups (Supplementary Table S6) based on their number of adenosine nucleotides added independently of a template to the 5′ poly(A) leader. The plot represents the percentage of sequences containing the 5′ mRNA cap in each group. The number of sequences containing the 5′ mRNA cap in transcripts with six or more non-templated adenosine nucleotides was very low (Supplementary Table S6); therefore, these transcripts were combined into a single group. The Pearson correlation coefficient value (*r*) is shown. The results are statistically significant at the *p* < 0.01 level. In total, 350 sequences obtained by 5′ RACE-PCR were used for this analysis.

We calculated the number of non-templated adenosine nucleotides in the 5′ leaders of the mRNAs of each pGKL ORF as well as the minimal number of templated and non-templated adenosines found at the 5′ ends of their uncapped transcripts and the fractions of capped mRNAs and mRNAs containing non-templated 5′ adenosine nucleotides. We also calculated the medians and means of the numbers of non-templated adenosine nucleotides added per mRNA molecule from each pGKL ORF. These data are summarized in Supplementary Tables S4, S5A. Further analysis of all the sequenced 5′ mRNA ends showed that the occurrence of 5′ mRNA capping was inversely correlated with the number of 5′ adenosines added independently of a template (Figure 3 and Supplementary Table S6). In harmony with that, longer 5′ poly(A) leaders were found in mRNAs transcribed from ORFs that yielded less frequently capped or completely uncapped transcripts (Figure 3 and Supplementary Tables S4, S5A). Congruently, the 5′ cap was more frequently present on the transcripts containing few or zero non-templated adenosine nucleotides at their 5′ ends (Supplementary Tables S4, S5A: *K2ORF2, K2ORF3, K2ORF4, K2ORF8*). However, both non-templated 5′ poly(A) leaders and mRNA caps were often found within identical transcripts (Figure 2A and Supplementary Table S5A).

**FIGURE 4:**
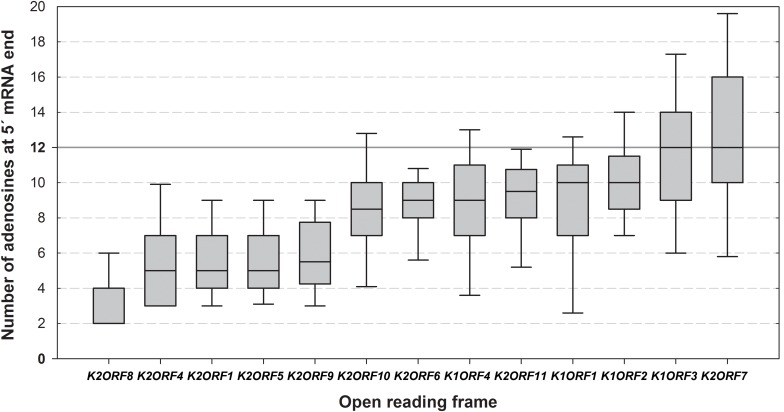
Differences in the lengths of the 5′ mRNA poly(A) leaders of individual pGKL ORFs. The box whisker plot represents the number of templated and non-templated consecutive adenosine nucleotides at the 5′ ends of pGKL mRNAs. The bottom and top of the box indicate the first and third quartiles, respectively. The whiskers indicate the 10th and 90th percentiles. The median is indicated as a solid black line. Outliers are not indicated. The open reading frames are ranked from left to right according to the prevalent lengths of the 5′ poly(A) leaders of their mRNAs. The value of 12 adenosine nucleotides represents an optimal length of the Pab1 binding site and is indicated as a solid gray line. The pGKL1 mRNAs belong to those with the longest 5′ poly(A) leaders. The data did not follow a normal distribution according to the Shapiro–Wilk test. The results were statistically analyzed using the non-parametric Kruskal–Wallis test, which supported rejection of the null hypothesis, *p*-value = 2.172940e-48. To discern gene pairs, the transcripts of which significantly differed in the lengths of their 5′ poly(A) leaders, we performed Dunn *post hoc* tests followed by adjustment of *p*-values according to the Benjamini–Hochberg FDR method. Adjusted *p*-values corresponding to all possible gene pairs are depicted in Supplementary Table S10. In total, 458 sequences obtained by 5′ RACE-PCR were used for this analysis (Supplementary Table S5A).

More than half of all pGKL ORFs (9) contained 5′ untranslated regions (UTRs) composed exclusively of adenosine nucleotides (Supplementary Tables S4, S5A) regardless of their templated or non-templated origin, thus resembling adenosine-rich yeast internal ribosome entry sites (IRESs) (Gilbert et al., 2007). The mRNAs transcribed from pGKL1 promoters tended to have longer and uncapped 5′ poly(A) leaders than pGKL2 mRNAs (Figure 4 and Supplementary Tables S4, S5A).

The 5′ ends of VLE mRNAs resemble the organization of intermediate and late vaccinia virus mRNAs (Schwer et al., 1987; Ahn and Moss, 1989), late cowpox virus mRNAs (Patel and Pickup, 1987), and even some plant mRNAs (Gowda et al., 2006) that also contain non-templated poly(A) leaders at their 5′ ends. However, all vaccinia mRNA transcripts are assumed to be fully capped (Kates and Beeson, 1970; Wei and Moss, 1974, 1975; Boone and Moss, 1977). Considering the aforementioned examples and combining our findings with the knowledge that the domain organization of the K2Orf3p putative capping enzyme resembles that of the poxviral capping enzyme (Larsen et al., 1998) allow speculation on the similarity between pGKL VLEs and poxviruses, at least at the levels of transcription and posttranscriptional modification. This hypothesis is further supported by our parallel work showing high similarity of pGKL1/2 RNA polymerase and promoters to poxviral RNA polymerases and promoters of early poxviral genes, respectively (Sýkora et al., 2018).

Our 5′ RACE analyses also identified three new functional UCS sites and the corresponding alternative transcription start sites (TSSs) of *K1ORF4*, *K2ORF3*, and *K2ORF4* (Supplementary Table S5A, marked as blue boxes).

### The pGKL Promoters Determine 5′ end mRNA Capping and Non-templated Polyadenylation

Mapping of the 5′ mRNA ends allowed us to determine putative UCS sites, UCR regions, and TSSs for all pGKL1 and pGKL2 ORFs. *K1ORF4*, *K2ORF3*, and *K2ORF4* contain two putative UCS sites each. We evaluated the nucleotide sequences of all these UCS sites and refined their previously reported consensus sequence, ATNTGA (Klassen and Meinhardt, 2007), by calculating the occurrences of all four nucleotides in the variable N position: A = 50%, G = 11%, C = 22%, and T = 17%. The TSS (+1 nucleotide) is usually located in a short window 17–22 nt downstream from the 3′ end of the UCS; the median distance is 19 nt, and the extreme values are 13 and 25 nt. Transcription initiates at the adenosine nucleotide. Two exceptions were present in the minor transcripts of *K2ORF2* and *K2ORF4*, which started with G and T, respectively. The minor upstream *K2ORF4* UCS ends with a guanosine nucleotide (ATATGG), an exception to the consensus sequence, and serves a non-canonical TSS, initiating with a T that is located only 13 nt downstream from the UCS and thus suggesting a slightly different mode of transcription initiation than that in other UCRs.

pGKL VLE UTRs are very short regardless of the presence or absence of 5′ non-templated leaders. Exceptions to this rule are the *K2ORF2* UTR (≈60 nt), *K2ORF3*_long_UTR (≈150 nt), *K2ORF3*_short_UTR (≈60 nt), and *K2ORF5* UTR (≈90 nt) (Supplementary Table S5A). Mapping the 5′ ends of pGKL transcripts allowed us to classify genes encoded by pGKL VLEs into two major groups: genes transcribed into frequently 5′ capped mRNAs with a low average number of 5′ non-templated adenosine nucleotides (*K2ORF2*, *K2ORF3*, *K2ORF4*, and *K2ORF8*; >50% of transcripts are 5′ capped and a median of added 5′ non-templated adenosines ≈ 0 per mRNA) and a larger group of genes producing transcripts less frequently 5′ capped but with a higher degree of 5′ non-templated polyadenylation (occurrence of 5′ capped transcripts varies between 0 and 50% and the median of added 5′ non-templated adenosines lies between 2 and 7) (summarized in Supplementary Table S4).

Our results are in very good agreement with three previous studies that used various setups of the classical primer extension method for 5′ UTR mapping. Collectively, those studies identified uninterrupted clusters of major and minor TSSs for the *K1ORF1*, *K1ORF2*, *K1ORF3*, *K1ORF4*, *K2ORF5*, and *K2ORF9* genes (Romanos and Boyd, 1988; Schaffrath and Meacock, 1995; Jeske et al., 2006). The lengths of 5′ UTRs detected by us and calculated as a sum of median lengths of 5′ poly(A) leaders and templated parts of the corresponding 5′ UTRs (Supplementary Table S4) were in agreement with the distances of previously suggested TSS clusters from the respective AUG codons of all these genes (Romanos and Boyd, 1988; Schaffrath and Meacock, 1995) except *K2ORF9*, for which the median length of the 5′ UTR detected by us was approximately 1 nt longer (Jeske et al., 2006). Our results show that a remarkable heterogeneity in the 5′ UTR lengths of pGKL transcripts is caused by the heterogeneity in the lengths of the non-templated 5′ poly(A) leaders (Figure 4 and Supplementary Tables S4, S5A) rather than by the presence of multiple TSSs. Good agreement of the 5′ UTR lengths determined by us with those mapped by different methods and in different laboratories also suggests that possible DNA polymerase slippage during PCR amplification and/or sequencing did not significantly alter determination of the 5′ poly(A) leader lengths in our experiments.

**FIGURE 5:**
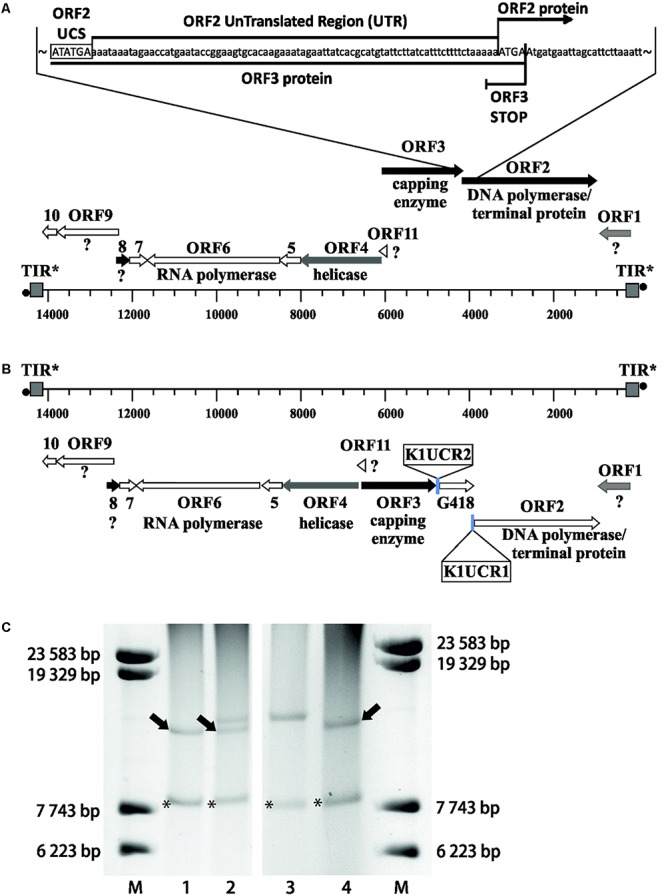
Precise manipulation of pGKL VLEs *in vivo* revealed an essential role of pGKL promoters in mRNA capping and non-template-based 5′ polyadenylation. **(A)** A closer view of the native pGKL2 region subjected to homologous recombination with a PCR cassette depicted in Supplementary Figure S5 shows a tightly packed VLE genome. The 3′ end of the *K2ORF3* coding region overlaps the *K2ORF2* promoter, 5′ UTR, and the first four nucleotides of the *K2ORF2* coding region. The pGKL2 VLEs displayed in **(B,C)** are in the reverse orientation of those in Figure 1. Shades of gray indicate the degree of transcript capping, as shown in Figure 1. **(B)** A PCR cassette containing an antibiotic resistance gene (G418) under the control of the ORF2 promoter from pGKL1 (*K1UCR2*) and the ORF1 promoter from pGKL1 (*K1UCR1*) (Supplementary Figure S5) was inserted into the *K2ORF2* promoter region by homologous recombination *in vivo*. The resulting VLE, pRKL2-1, contains two genes, aminoglycoside 3′-phosphotransferase (coding for G418 resistance) and *K2ORF2*, that are artificially controlled by the pGKL1 promoters *K1UCR2* and *K1UCR1*, respectively. Shades of gray indicate the degree of transcript capping, as shown in Figure 1. The 5′ RACE results of pRKL2-1-encoded mRNAs are summarized in the text and in Supplementary Table S7. **(C)** Electrophoretic analysis of pGKL VLEs in *K. lactis* clones. M, lambda DNA/*Eco*130I (*Sty*I) marker (Fermentas); lanes 1 and 4, native pGKL VLEs from *K. lactis* IFO1267 (pGKL1 [8874 bp] is labeled with an asterisk, and pGKL2 [13447 bp] is labeled with an arrow); lane 2, linear VLEs purified from *K. lactis* IFO1267 carrying both the recombinant (higher MW) and wild-type pGKL2 VLEs; lane 3, linear VLEs purified from *K. lactis* IFO1267 containing the recombinant pRKL2-1 VLE (14353 bp). The shorter wild-type pGKL2 was lost after cultivation for ≈60 generations in selective medium containing G418.

We wanted to investigate whether UCR sequences can contain signals that determine the degree of 5′ end polyadenylation and the frequency of 5′ capping of the pGKL transcripts. For this purpose, we prepared a yeast strain with altered pGKL VLEs. Genomes of pGKL VLEs are extremely tightly packed (Figures 1, 5A) (Stark et al., 1990) and can be manipulated only by homologous recombination *in vivo* (Kamper et al., 1989). For genetic engineering of pGKL VLEs, we developed a unique fusion PCR strategy followed by homologous recombination *in vivo* that allowed us to precisely modify selected pGKL regions, including the introduction of point mutations. Using this approach, we prepared a *K. lactis* IFO1267 strain bearing a new cytoplasmic linear VLE pRKL2-1 (for details of the method, refer to Figure 5 and Supplementary Figure S5, and the section Materials and Methods). The pRKL2-1 linear VLE is a pGKL2 derivative that contains a *G418* resistance marker (*G418*^*R*^) under the control of *K1UCR2* and *K2ORF2* artificially controlled by *K1UCR1* (Figure 5 and Supplementary Figure S5). The presence of a cap at the 5′ end of pRKL2-1 mRNAs coding for *K2ORF2* and *G418*^*R*^ as well as the presence and length of a 5′ poly(A) leader were both directly influenced by the UCR sequence used for expression of these genes. Replacement of the wild-type UCR sequence of the *K2ORF2* gene by the *K1ORF1* UCR sequence (*K1UCR1*, Figure 5) led to a remarkable switch from nearly fully capped and almost no 5′ polyadenylated transcripts (91.7% capped transcripts, average of added non-templated adenosine nucleotides per mRNA molecule ≈ 0) to largely uncapped and 5′ polyadenylated transcripts (7.7% capped transcripts, average of added non-templated adenosine nucleotides per mRNA molecule ≈ 5). These values correspond very well to the level of 5′ capping and non-template polyadenylation of the wild-type *K1ORF1* mRNAs expressed from pGKL1 VLE under the control of natural *K1UCR1* (Figure 5, Supplementary Figure S2, and Supplementary Table S7). The same trend was also observed for the *G418*^*R*^ transcripts, the transcription of which was controlled by *K1UCR2.* The majority of these transcripts were uncapped and 5′ polyadenylated (96.4%) in exactly the same manner as the natural *K1ORF2* transcripts. The average number of non-templated adenosine residues in the 5′ leader was comparable in both *G418*^*R*^ and *K1ORF2* genes, controlled by *K1UCR2.* These findings strongly support our recent experiments showing that point mutations within the TSS regions of pGKL genes can substantially influence the 5′ polyadenylation of the pGKL mRNAs (Sýkora et al., 2018) and further support the hypothesis that differences in the 5′ end formation, both 5′ capping and polyadenylation, of yeast linear VLE mRNAs are controlled by UCR sequences and are independent of the coding sequence of a gene and its location within the pGKL VLE system.

**FIGURE 6:**
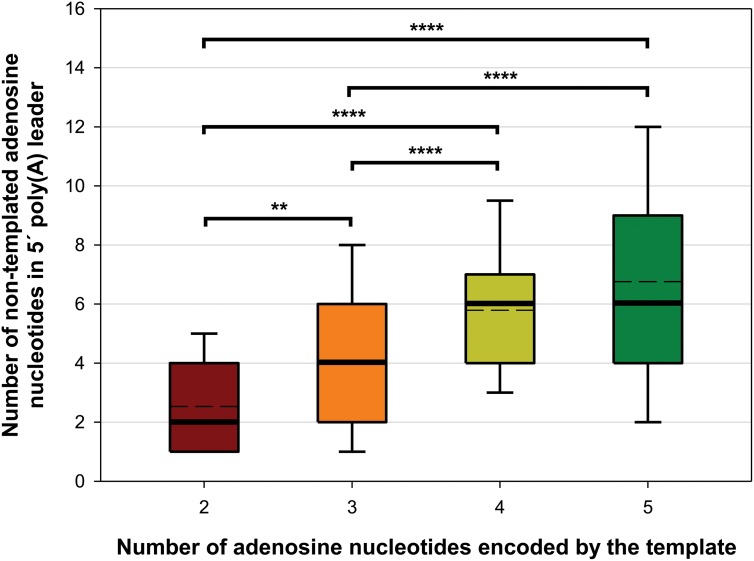
The number of 5′ non-templated adenosine nucleotides in the 5′ leaders of pGKL transcripts increases with the number of adjacent consecutive adenosines encoded by the template. The box whisker plot represents the number of non-templated adenosine nucleotides in the 5′ poly(A) leaders of pGKL mRNAs classified according to the length of the adjacent consecutive adenosines encoded by the DNA template at the TSS. The bottom and top of the box indicate the first and third quartiles, respectively. The whiskers indicate the 10th and 90th percentiles. The median and mean are indicated as bold and dashed lines, respectively. Outliers are not indicated. Two adenosines are encoded in the TSS of the *K2ORF8* promoter (red). Three adenosines are encoded in the TSS of the *K1ORF4*, *K2ORF1*, *K2ORF5*, *K2ORF9*, and *K2ORF11* promoters (orange). Four adenosines are encoded in the TSS of the *K1ORF2*, *K2ORF6*, and *K2ORF10* promoters (yellow). Five adenosines are encoded in the TSS of the *K1ORF1*, *K1ORF3*, *K2ORF4*, and *K2ORF7* promoters (green). The results suggest pGKL RNA polymerase slippage at the TSS as a mechanism of 5′ mRNA poly(A) leader synthesis. The data did not follow a normal distribution according to the Shapiro–Wilk test. The results were statistically analyzed using a non-parametric Kruskal–Wallis test followed by a *post hoc* Dunn test with *p*-value adjustment according to the Benjamini–Hochberg FDR method. Adjusted *p*-values corresponding to all possible pairs are depicted in Supplementary Table S11. ^∗∗^Significance level *p* < 0.01; ^∗∗∗∗^significance level *p* < 0.0001. In total, 373 sequences obtained by 5′ RACE-PCR containing non-templated adenosine nucleotides were used for this analysis (Supplementary Table S8).

Synthesis of 5′ poly(A) leaders of the poxviral late transcripts is caused by the RNA polymerase slipping on the three consecutive thymidines within the TSS site (Schwer et al., 1987; Schwer and Stunnenberg, 1988; Davison and Moss, 1989b). We recently showed that a similar sequence motif is present in the TSS of pGKL genes and that its alteration can affect 5′ polyadenylation of pGKL mRNAs (Sýkora et al., 2018). We analyzed the nucleotide composition directly adjacent to the 5′ non-templated adenosines of all pGKL1/2 mRNAs, revealing that the number of 5′ non-templated adenosines significantly increased with the number of directly contiguous and consecutive adenosine nucleotides coded by the DNA template (Figure 6 and Supplementary Table S8). All these results suggested pGKL-specific RNA polymerase slippage on consecutive thymidine residues of the coding DNA strand at the TSS, which appeared to be pronounced by an increased number of coded consecutive adenosines (Figure 6 and Supplementary Table S8). The latter is in contrast to poxviral transcripts, where significant differences in the lengths of the 5′ poly(A) leaders between intermediate and late mRNAs could not be attributed to differences in the number of coded consecutive adenosines at the TSS because only three consecutive adenosines are typically coded at their TSS (Yang et al., 2012).

**FIGURE 7:**
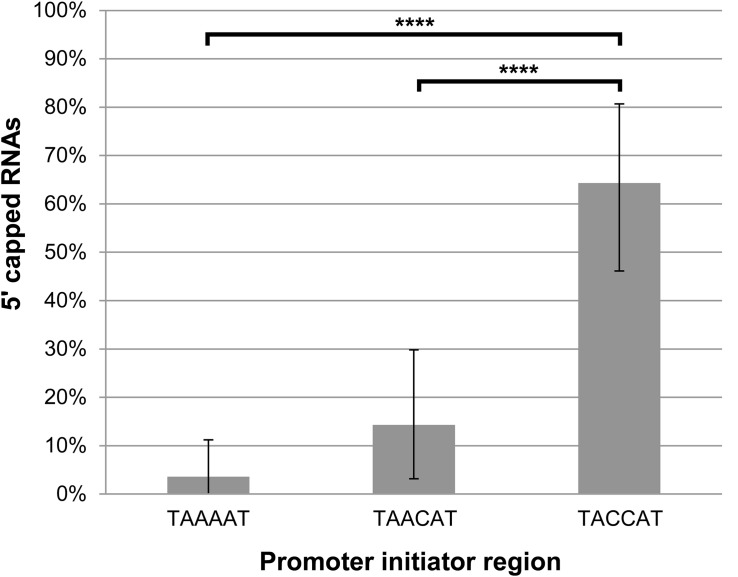
The lengths of the adenosine stretches within the promoter initiator regions of pGKL mRNAs inversely correlate with the proportion of their 5′ capping. The presence of 5′ RNA caps was monitored using 5′ RACE analysis of the *G418*^*R*^ gene from the IFO1267_pRKL1-1 strain (TAAAAT initiator region), IFO1267_pRKL1-2 strain (TAACAT initiator region), and IFO1267_pRKL1-3 strain (TACCAT initiator region), followed by cloning and sequencing of individual cDNA clones (26 clones for each strain). Bars represent the frequency (in %) of 5′ RNA capping, with the error bars depicting the 95% confidence intervals calculated using the adjusted Wald method. The results were statistically evaluated using two-tailed Fisher’s exact test with a 95% confidence interval. ^∗∗∗∗^Significance level *p* < 0.0001.

Next, we wanted to investigate our finding that the lengths of the 5′ poly(A) leaders of pGKL mRNAs inversely correlate with the proportion of their 5′ capping (Figure 3) in more detail. We analyzed 5′ cap occurrence in transcripts of the *G418*^*R*^ gene, which was inserted under the control of *K1UCR2* within the engineered pRKL1-1 linear VLE and its pRKL1-2 and pRKL1-3 derivatives. The TAAAAT sequence of the *K1UCR2* TSS was modified to TAACAT and TACCAT in pRKL1-2 and pRKL1-3, respectively. We showed in our parallel study that mutations in *K1UCR2* TSS led to shortening (in the case of TAACAT TSS) or even loss (in the case of TACCAT TSS) of the 5′ poly(A) leaders of the *G418*^*R*^ transcripts (Sýkora et al., 2018). In accordance with our global analysis of all pGKL transcripts (Figure 3), progressive shortening and loss of 5′ poly(A) mRNA leaders caused by incremental substitutions of A to C within *K1UCR2* TSS (Sýkora et al., 2018) led to the progressive increase in the 5′ cap occurrence from 3.6% of the 5′ capped mRNAs starting from the wild-type TSS to 64.3% of 5′ capped mRNAs starting from the two-point-mutated pRKL1-3 TSS (TACCAT, Figure 7). To our knowledge, this is the first known example of promoter sequence interference in 5′ mRNA capping.

We speculate that the pGKL transcription machinery core complex comprising RNA polymerase subunits (K2Orf6p and K2Orf7p) and an mRNA capping enzyme (K2Orf3p) (Sýkora et al., 2018) undergoes conformational changes influenced by RNA polymerase slippage at the TSS region that can lead to the decreased frequency of 5′ mRNA cap synthesis. Interestingly, nucleotide substitutions and deletions located just downstream of the essential TAAAT motif within the initiator region of the vaccinia virus late promoters led to the variation in lengths of the 5′ poly(A) leaders of the corresponding viral mRNAs (de Magistris and Stunnenberg, 1988). This result also suggests that other parts of the pGKL promoters rather than only the TSS might influence the lengths of the 5′ poly(A) leaders and the frequency of 5′ mRNA capping. In contrast to our results showing frequent occurrences of the pGKL transcripts lacking 5′ caps (Supplementary Table S4), both vaccinia virus early and postreplicative transcripts are assumed to be fully 5′ capped (Kates and Beeson, 1970; Wei and Moss, 1974, 1975; Boone and Moss, 1977).

Our analyses of the pGKL and pRKL2-1 mRNA ends showed the importance of the UCR region for 5′ mRNA end formation and may help to explain the surprising results of Schickel et al. (1996) who used a glucose dehydrogenase (*gdh*) reporter gene integrated into yeast cytoplasmic linear VLEs to measure the strengths of several pGKL promoters. They showed that *K2UCR6* controlling the *gdh* reporter resulted in almost twofold higher expression than that achieved when the reporter was controlled by *K2UCR10* even though these two UCRs have exactly the same length and UCS sequence (Schickel et al., 1996). We found that 20% of *K2ORF6* transcripts possessed a 5′ cap, whereas only uncapped mRNAs were found among *K2ORF10* transcripts (Supplementary Tables S4, S5A). A 5′ cap positively affected the translation of mRNAs carrying a 5′ poly(A) leader in a wheat germ extract (WGE) *in vitro* (Gudkov et al., 2005). If a 5′ cap substantially increases the translation efficiency of pGKL transcripts, differences in cap occurrence frequency can account for differences in the expression of pGKL genes.

### The Cap Moiety at the 5′ Ends of pGKL Transcripts Can Be Removed by hDcp2 but Not by Rai1 Decapping Enzymes

Recent advances in the understanding of mRNA quality control and the function of closely associated cellular mRNA decapping enzymes allowed us to use the *S. pombe* Rai1 and human Dcp2 proteins to indirectly investigate structural features of the 5′ cap at pGKL mRNAs, including its presumed guanosine N7-methylation. Both proteins were produced in the *E. coli* BL21(DE3) strain and purified to homogeneity exactly as described by Xiang et al. (2009). For the cap analysis, we purified total RNA from the killer *K. lactis* IFO1267 strain. Total RNA was DNase I-treated and subsequently incubated with either purified Rai1 decapping enzyme, which removes unmethylated caps only (Jiao et al., 2010), or purified hDcp2 decapping enzyme, which can remove both methylated and unmethylated guanosine caps from the 5′ ends of mRNAs (Wang et al., 2002; Song et al., 2013), despite the fact that hDcp2 activity is stimulated by the presence of the N7-methyl group and requires a long RNA molecule attached to the 5′ cap (van Dijk et al., 2002).

**FIGURE 8:**
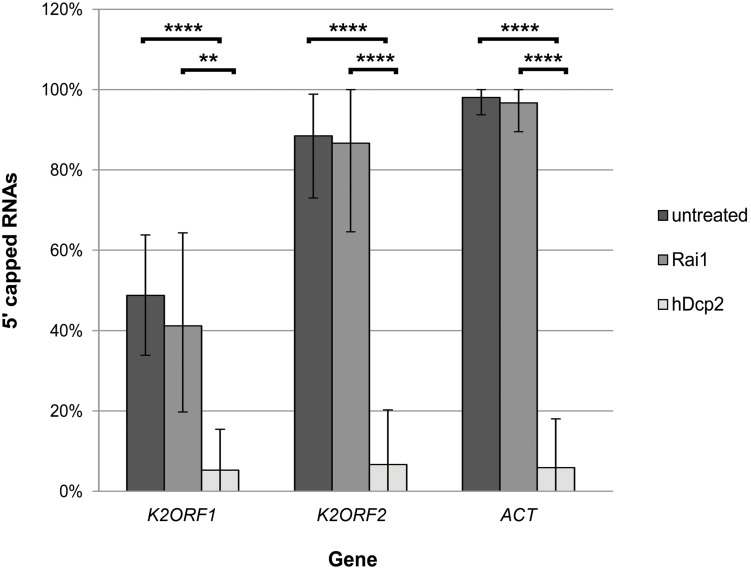
The 5′ ends of pGKL mRNAs are capped with the N7-methyl guanosine. Total RNA prepared from *K. lactis* IFO1267 was used for this experiment. The RNA was untreated (dark gray) or treated with either the Rai1 (middle gray; removes unmethylated 5′ mRNA caps only) or hDcp2 (light gray; removes both methylated and unmethylated 5′ mRNA caps) mRNA decapping enzymes. The presence of 5′ RNA caps after incubation was monitored using 5′ RACE followed by cloning and sequencing of individual cDNA clones. Bars represent the frequency (in %) of 5′ RNA capping, with the error bars depicting the 95% confidence intervals calculated using the adjusted Wald method. The results were statistically evaluated using two-tailed Fisher’s exact test with a 95% confidence interval. ^∗∗^Significance level *p* < 0.01; ^∗∗∗∗^significance level *p* < 0.0001. The 5′ RACE analysis of independent cDNA clones is summarized in Supplementary Table S12. The results clearly show that pGKL mRNAs were resistant to decapping by Rai1, similar to the actin control, whereas all tested mRNAs were significantly decapped by hDcp2.

Treatment of *K. lactis* total RNA with hDcp2 significantly decreased the number of capped pGKL mRNAs represented by the originally frequently capped transcripts from the *K2ORF1* and *K2ORF2* genes, whereas treatment with Rai1 had no significant effect. Identical results were obtained for the control actin-coding mRNA (Figure 8 and Supplementary Table S12). In agreement with the theoretical expectation, we observed exclusive C insertion at the very ends of cDNAs copied from 5′ cap-containing mRNAs during all 5′ RACE experiments described herein. All these results strongly suggest that capped pGKL transcripts contain typical guanine N7-methylated caps, just like the endogenous cellular transcripts represented by nuclear-coded actin mRNA in this experiment.

During evolution, yeast linear plasmids were apparently enforced to retain a sufficient repertoire of genes coding for DNA and RNA polymerases and essential accessory proteins to ensure autonomous maintenance of plasmids in the cell cytoplasm (Klassen and Meinhardt, 2007). The possible presence of the standard guanine N7-methylated cap on some pGKL mRNAs (Figure 2A and Supplementary Tables S4, S5) further indirectly supports the previous evidence that K2Orf3p functions as a canonical capping enzyme either alone or as a part of an unknown protein complex, exhibiting all three essential enzymatic activities necessary for complete conventional m7G cap synthesis (Larsen et al., 1998; Tiggemann et al., 2001).

### pGKL mRNAs Do Not Contain 3′ Poly(A) Tails

The unique 5′ ends of pGKL mRNAs immediately raised a question about the organization of their 3′ ends. It was reported previously that pGKL mRNAs could be readily purified by oligo(dT) columns, which might be an indication of polyadenylation at their 3′ ends or, alternatively, be a result of their A/T-rich nature (Sor and Fukuhara, 1985; Stark et al., 1990). Possible 3′ polyadenylation is reasonable, especially in the case of non-capped pGKL mRNAs, because 3′ poly(A) tails on eukaryotic mRNAs play an important role in their stability (Santiago et al., 1987; Bernstein et al., 1989) and in their efficient utilization by cellular translation apparatuses (Munroe and Jacobson, 1990; Gallie, 1991; Tanguay and Gallie, 1996). However, pGKL VLEs likely do not encode their own poly(A) polymerase (Figure 1), and it remains unknown whether the host cellular polyadenylation machinery participates in the processing of pGKL-specific 3′ mRNA ends.

To answer this question, we performed a modified 3′ RACE in which purified and DNase I-treated total yeast RNA was 3′ tailed by an *E. coli* poly(A) polymerase using CTP instead of ATP as the substrate. Following reverse transcription with an oligo(dG)-anchored primer, PCR amplifications were performed with a universal anchor primer and appropriate gene-specific primers. At least 20 randomly selected 3′ RACE fragments cloned into pCR4-TOPO plasmids were sequenced for each ORF. The 3′ RACE analyses of all 15 pGKL ORFs are exemplified by the results of *K1ORF1* (Figure 2B and Supplementary Table S5C), *K2ORF5*, and *K2ORF10* (Supplementary Table S5C).

We found that pGKL mRNAs do not contain a 3′ poly(A) tail. Transcripts produced from pGKL VLEs contain heterogeneous 3′ ends, suggesting the existence of more than one independent putative termination signal. Transcripts corresponding to only four ORFs are uniform at their 3′ ends. This group is exemplified by *K2ORF10* (Supplementary Table S5C). Four pGKL ORFs gave rise to transcripts that fell into two distinct groups with regard to their 3′ UTR lengths, suggesting two independent transcription terminators. These transcripts are exemplified by those of *K2ORF5* (Supplementary Table S5C). Transcripts of the remaining seven pGKL ORFs probably utilize three or more termination sites. Weak RNA stem loops at the 3′ ends of the pGKL mRNAs serve as transcription termination signals, which we recently analyzed in detail (Sýkora et al., 2018). Collectively, these results suggest that cellular 3′ pre-mRNA cleavage and polyadenylation machinery do not act on pGKL mRNAs. However, pGKL transcripts can be engineered to contain 3′ poly(A) tails, and it was recently shown that a genetically encoded 3′ poly(A) tail enhances the expression of a reporter gene located on the pGKL VLE (Zhong et al., 2018).

### Cellular mRNAs Outcompete pGKL mRNAs in Binding to the Yeast Cap-Binding Protein eIF4E *in vitr*o

The presence of a methylguanosine cap at their 5′ ends and 3′ polyadenylation are common hallmarks of eukaryotic mRNAs. The absence of these structures in the pGKL transcripts should lead to their decreased stability and low translatability (Gallie, 1991; Tarun and Sachs, 1995; Tanguay and Gallie, 1996). However, this is clearly not the case for pGKL VLEs, which are stably maintained in yeast cells, and their genes thus must be expressed to the level ensuring autonomous VLE replication in the host cytoplasm. Previous experiments showed that pGKL mRNAs can be easily detected by northern blot (Stark et al., 1990). Schickel et al. investigated the strength of pGKL UCRs (promoters) using the bacterial glucose dehydrogenase gene (*gdh*) as a reporter, revealing that *gdh* expression from the yeast linear cytoplasmic VLEs was weaker than that from bacterial systems and yeast nuclear promoters. However, they were able to detect glucose dehydrogenase activity for all eight pGKL UCRs tested (Schickel et al., 1996). It should also be noted that the toxin encoded by the pGKL1 VLE is secreted in high amounts into the culture medium (Stark and Boyd, 1986, and next paragraph) despite the fact that transcripts coding for the toxin subunits and immunity-related proteins have the lowest cap occurrence frequency among all pGKL transcripts (*K2ORF2*—7.5%, *K2ORF3*—5.3%, *K2ORF4*—17.4% of capped mRNAs; Supplementary Tables S4, S5A).

To investigate this phenomenon, we designed a series of *in vitro* and *in vivo* experiments in which we demonstrated that VLE mRNAs bearing 5′ poly(A) leaders could be expressed in an eIF4E-independent manner. Production of the killer toxin encoded by pGKL1 mRNAs, which are particularly less frequently capped and highly 5′ polyadenylated, was used as a sensitive natural reporter for direct investigation of the effect of eIF4E loss on pGKL gene expression.

The eIF4E protein plays a crucial role in eukaryotic translation initiation. It recognizes and binds m^7^G cap structures at the 5′ ends of eukaryotic mRNAs and brings them to the initiating small ribosomal subunits *via* its interaction with scaffold factor eIF4G. The eIF4E protein is essential in all eukaryotes, and its deletion is also lethal in yeast (Altmann et al., 1987; Brenner et al., 1988; Giaever et al., 2002). The structure and function of the cap-binding eIF4E protein from the yeast *S. cerevisiae* is very well understood. Because pGKL VLEs can be transferred and stably maintained in *S. cerevisiae* (Gunge and Sakaguchi, 1981), we decided to test the strength of the interaction between the *S. cerevisiae* eIF4E cap-binding protein (S.c.-eIF4E) and pGKL mRNAs in the presence of excess total RNA *in vitro.*

**FIGURE 9:**
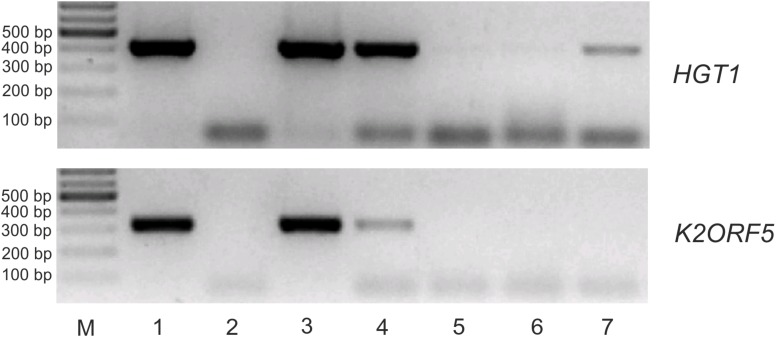
*K2ORF5* mRNA is outcompeted by cellular RNA in binding to the yeast cap-binding protein eIF4E *in vitro*. Electrophoretic analysis of semiquantitative RT-PCR detecting control *HGT1* mRNA **(upper panel)** and *K2ORF5* mRNA **(lower panel)** in samples as follows: Lane 1, glutathione-Sepharose with the bound GST-eIF4E fusion protein in the presence of excess *K. lactis* IFO1267 total RNA (input); lane 2, same as in line 1 but the reaction was performed without reverse transcriptase (negative control); lane 3, supernatant from the first wash step (unbound mRNA); lanes 4, 5, and 6, supernatants after the second, third, and sixth wash steps, respectively (unbound mRNA); lane 7, mRNA remaining bound on GST-S.c-eIF4E Sepharose after the sixth wash step. M, GeneRuler 100-bp DNA Ladder Plus (Thermo Scientific). PCR was performed using cDNA, Taq DNA polymerase, and the gene-specific primers listed in Supplementary Table S1. All washing steps were performed with 70 volumes of buffer I. The initial abundances of *HGT1* and *K2ORF5* mRNA in the *K. lactis* total RNA were comparable as determined by qRT-PCR (Supplementary Figure S3).

We produced S.c.-eIF4E as an N-terminal GST-fusion protein in *E. coli* and purified it by glutathione-Sepharose affinity chromatography. To test the interaction of pGKL-specific mRNAs with yeast eIF4E in the presence of excess cellular RNA, we incubated DNase I-treated total RNA purified from *K. lactis* IFO1267 with the purified GST-S.c.-eIF4E fusion protein bound to glutathione-Sepharose. After 3 h of incubation at room temperature, the slurry was extensively washed six times with a substantial excess of buffer I at room temperature and directly used in RT-PCRs with gene-specific primers. Figure 9 clearly shows that the *K2ORF5* mRNA was completely removed from the GST-S.c-eIF4E Sepharose in the first two wash steps (Figure 9, lanes 3 and 4), whereas cellular mRNAs, represented by mRNA coding for a high-affinity glucose transporter (*HGT1*), remained bound to the eIF4E even after six thorough wash steps (Figure 9, lanes 6 and 7). This result clearly indicates that, contrary to *HGT1* mRNA, *K2ORF5* mRNA does not bind to yeast eIF4E *in vitro* and/or binds to it with low affinity and can be therefore outcompeted by an excess of yeast cellular mRNAs. This result is further supported by qRT-PCR analyses revealing comparable abundances of *K2ORF5* and *HGT1* mRNAs in the *K. lactis* IFO1267 total RNA (Supplementary Figure S3).

Interestingly, *K2ORF5* does not belong to the group of pGKL mRNAs with the lowest frequency of 5′ m^7^G cap occurrences (Supplementary Tables S4, S5A), potentially because the strength of eIF4E binding to mRNA is highly influenced by the first proximal nucleotides following the terminal m^7^G. For the first such nucleotide, the association constant *K*_*a*_ is reportedly much higher for purines than for pyrimidines and slightly higher for guanosine than for adenosine (Wieczorek et al., 1999; Tamarkin-Ben-Harush et al., 2017). Many similar results have also been obtained by other researchers; however, cap analogs were used to determine the *K*_*a*_ and *K*_*d*_ values in most of these studies. The presence of more nucleotides after the first proximal nucleotide substantially increased the ability of eIF4E to bind the capped RNA molecule while still preserving the differential influence of the first proximal nucleotide on eIF4E binding (Tamarkin-Ben-Harush et al., 2017). A short homopolymeric stretch of 5′ adenosine nucleotides thus might destabilize the binding of eIF4E to the mRNA cap. However, decreased binding of pGKL mRNAs to eIF4E in competition with an excess of cellular RNA *in vitro* does not correspond to any possible substantial decrease in the pGKL mRNA performance during translation *in vivo*, as evidenced by the stable cellular maintenance of pGKL1/2 VLEs, which may suggest an unusual mechanism of translation initiation independent of the eIF4E cap-binding protein.

### The Killer Toxin Encoded by pGKL VLEs Is Produced by an eIF4E-Independent Pathway

The fact that pGKL mRNAs were outcompeted by cellular mRNAs in binding to the yeast cap-binding protein eIF4E *in vitro* prompted us to investigate whether these mRNAs require a functional eIF4E-dependent pathway for their translation. The reliable determination of whether a specific mRNA is translated 5′ cap-independently and/or eIF4E-independently *in vivo* is rather complicated (Mokrejs et al., 2010). We employed yeast strains bearing conditional mutations in the *CDC33* gene, which codes for eIF4E. The *S. cerevisiae* CWO4 strains carrying either a *cdc33-1* or *cdc33-42* allele as a sole source of the eIF4E translation initiation factor (kindly obtained from Prof. Michael Altman) show a strong drop in protein synthesis (Altmann et al., 1989; Altmann and Trachsel, 1989) and remarkably decreased polysomes (Feketova et al., 2010) at an elevated temperature. We decided to transfer pGKL VLEs into these strains and determine whether a reasonable amount of the functional pGKL toxin is produced at 37°C, where Cdc33-1 and Cdc33-42 proteins are not functional and eIF4E-dependent translation initiation is thus impaired (Altmann et al., 1989; Altmann and Trachsel, 1989). At least three genes (*K1ORF2*, *K1ORF3*, and *K1ORF4*) encoded by pGKL1 VLEs are required to be sufficiently expressed in yeast cells to impart killer and immunity phenotypes. The killer toxin itself is a trimeric protein comprising non-identical alpha, beta, and gamma subunits that are encoded by *K1ORF2* and *K1ORF4* (Figure 1) (Sugisaki et al., 1984; Stark and Boyd, 1986). Interestingly, transcripts of all three of these genes belong to the mRNA group with the lowest cap occurrence frequency and a high degree of 5′ polyadenylation (Supplementary Tables S4, S5A and Figure 4). The presence of the killer phenotype under restrictive conditions, when cellular eIF4E-dependent translation initiation is impaired, would thus indicate that at least three pGKL genes can be sufficiently and coordinately expressed in an eIF4E-independent manner.

**FIGURE 10:**
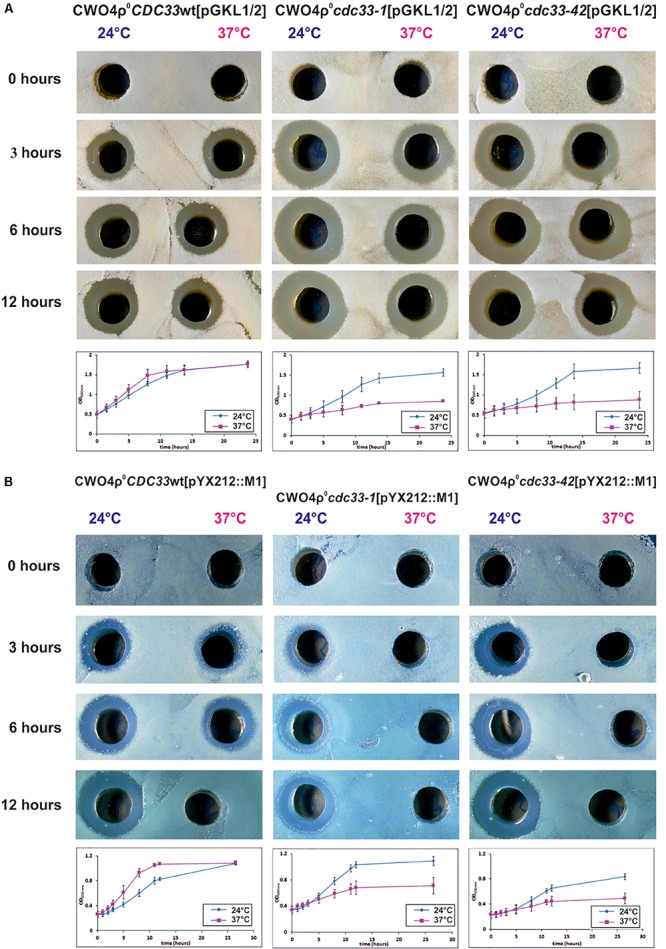
The killer toxin encoded by pGKL VLEs is synthesized by the eIF4E-independent pathway. **(A)** Production of pGKL killer toxin in *S. cerevisiae* CWO4ρ^0^ strains bearing cytoplasmic pGKL VLEs and different *CDC33* alleles. The pGKL toxin-coding mRNAs do not contain 3′ poly(A) tails and are mostly 5′ uncapped and 5′ polyadenylated (Supplementary Tables S4, S5A and Figure 6). **(B)** Production of K1 killer toxin in control *S. cerevisiae* CWO4ρ^0^ strains containing pYX212::M1 plasmid and different *CDC33* alleles. The control mRNA coding for K1 toxin is transcribed in the nucleus by RNA polymerase II. In both cases, the results are shown for *S. cerevisiae* CWO4ρ^0^ strains bearing either the wild-type *CDC33* (eIF4E) gene (*CDC33*wt) or temperature-sensitive mutations in the *CDC33* (eIF4E) gene (*cdc33-1* and *cdc33-42* alleles) grown under both permissive (24°C) and non-permissive (37°C) conditions. Aliquots were taken at 0, 3, 6, and 12 h, and the culture medium was filter sterilized, diluted 10×, and assayed for killer toxin activity by an agar well diffusion test using a lawn of *S. cerevisiae* S6/1 sensitive strain cells. Growth curves of all strains at the permissive (blue) and non-permissive (purple) temperatures are shown below each of the corresponding sets of killer toxin experiments. Each growth curve represents at least three independent experiments. Error bars correspond to standard deviation.

The naturally occurring artificial transfer of pGKL VLEs from *K. lactis* into *S. cerevisiae* is rare and difficult. In addition to other obstacles, the host mitochondria and pGKL VLEs in *S. cerevisiae* are incompatible, and the recipient strain must thus be devoid of mitochondrial DNA (ρ^0^) (Gunge and Yamane, 1984). We prepared CWO4 ρ^0^ strains bearing either wild-type *CDC33* or its temperature-sensitive *cdc33-1* or *cdc33-42* alleles by their long-term cultivation in the presence of ethidium bromide. To facilitate the transfer of pGKL VLEs to the new strains and to preserve their isogenic genetic background, we performed cytoduction by incomplete mating (Zakharov et al., 1969) the CWO4 ρ^0^ strains with a nuclear fusion-deficient (*kar1*) yeast strain (*S. cerevisiae* YAT547) carrying both pGKL1 and pGKL2 VLEs (Gunge and Yamane, 1984). We obtained isogenic ρ^0^ and [pGKL1/2] *S. cerevisiae* strains on the CWO4 genetic background that differed only in their *CDC33* alleles. All these strains contain both pGKL VLEs and display a killer phenotype (Figure 10A).

We required a gene coding for a yeast single-subunit protein toxin to serve as a control for eIF4E-dependent translation. For this purpose, we chose a thoroughly studied K1 killer toxin encoded by the M1 viral satellite dsRNA from the yeast *S. cerevisiae.* We prepared the M1 cDNA, cloned a gene coding for the prepro-K1 killer toxin into a yeast shuttle expression plasmid (Valis et al., 2006) under the control of the strong constitutive *TPI* promoter (plasmid pYX212::M1) and introduced this expression plasmid into all three *S. cerevisiae* CWO4 ρ^0^ strains carrying different *CDC33* alleles as described above. The resulting strains displayed the K1 killer and immunity phenotypes at the permissive temperature (24°C) (Figure 10B).

The experiment depicted in Figure 10 shows a comparison of the production of the *K. lactis* toxin expressed naturally from pGKL1 VLEs and the K1 toxin expressed artificially from the nuclear plasmid pYX212::M1. The K1 toxin gene was transcribed by RNA polymerase II in the nucleus and thus provided a 5′ m^7^G capped and 3′ polyadenylated mRNA control. We cultivated all the CWO4 ρ^0^ [pGKL1/2] and CWO4 ρ^0^ [pYX212::M1] strains in parallel to the exponential phase at the permissive temperature (24°C). All the strains were then harvested and briefly washed three times with an excess of fresh medium preheated to 24°C. Half of each culture was further cultivated at 24°C. The second half was rapidly transferred to fresh medium preheated to 37°C and further cultivated at this elevated temperature. At this point, initial samples marked as zero were taken for biomass measurements and determination of toxin activity in the culture medium. Toxin activities were undetectable in all samples at time zero and provided clear evidence that all cultures were washed sufficiently and that the toxin activities present in samples taken after 3, 6, and 12 h of cultivation corresponded to proteins produced exclusively during the experiment (Figure 10).

Importantly, production of the *K. lactis* killer toxin in *S. cerevisiae* strains bearing pGKL VLEs was almost comparable under permissive and restrictive conditions regardless of whether the wild-type or temperature-sensitive *CDC33* allele was present (Figure 10A; 37°C). Production of the K1 toxin in the *cdc33-1* and *cdc33-42* yeast strains containing the pYX212::M1 plasmid vanished rapidly at 37°C, whereas the wild-type Cdc33p cap-binding protein permitted K1 toxin production at both 24 and 37°C (Figure 10B). The presence of temperature-sensitive eIF4E proteins in *cdc33-1* and *cdc33-42* yeast strains was documented by their restricted growth at 37°C. Both strains bearing the wild-type allele of the *CDC33* gene grew well at both 24 and 37°C (Figure 10). The growth of CWO4 *CDC33*, ρ^0^ [pYX212::M1] was slightly slower at 24°C than at 37°C, most likely because the K1 killer toxin is more active at 24°C than at 37°C, even though we cloned the coding sequence of the K1 superkiller variant that shows enhanced thermal stability (Palfree and Bussey, 1979). We previously showed that K1 preprotoxin produced artificially from the nuclear plasmid does not fully support the immunity phenotype of the host cells (Valis et al., 2006). Higher K1 toxin stability at 24°C thus probably led to slightly slower growth. However, both cultures eventually reached comparable biomasses, in contrast to the strains bearing conditional *cdc33*^ts^ alleles cultured at a restrictive temperature (37°C), which completed the initiated cell division and ceased growth (Figure 10B). The activity of the *TPI* promoter used to produce the K1 toxin from the pYX212::M1 plasmid declines at the end of exponential growth (Partow et al., 2010), and this phenomenon is even more profound in the version of the *TPI* promoter used in the pYX212 plasmid (Novák, 2012). The growth of the CWO4 *CDC33*, ρ^0^ [pYX212::M1] strain was slightly faster at 37°C than at 24°C, causing the culture to reach early stationary phase in 8 h (instead of 12 at 24°C), and this difference explains why K1 toxin production ceased in the *CDC33* wild-type strain grown for 12 h at 37°C (Figure 10B).

The most straightforward explanation of the results obtained in this experiment is that whereas K1 toxin mRNA was transcribed from the shuttle plasmid under the control of the *TPI* promoter by the nuclear RNA polymerase II and was thus 5′ capped, 3′ polyadenylated, and translated apparently only by the canonical eIF4E-dependent pathway, pGKL1 toxin mRNAs retained their ability to be translated even when 5′ mRNA cap recognition by the cellular translation initiation machinery was severely impaired. pGKL1 mRNAs are transcribed by the pGKL VLE-specific RNA polymerase encoded by *K2ORF6/7* and contain unusual 5′ stretches of non-templated adenosine nucleotides rarely covalently linked to the terminal m^7^G caps (Supplementary Tables S4, S5A and Figures 1, 2A, 3, 4). Our experiments, both *in vitro* (Figure 9) and *in vivo* (Figure 10), strongly suggest that these 5′ terminal structures of the pGKL mRNAs support a novel manner of translation initiation independent of the active eIF4E protein.

### 5′ Polyadenylated and Uncapped VLE mRNAs Associate With High Polysomes

**FIGURE 11:**
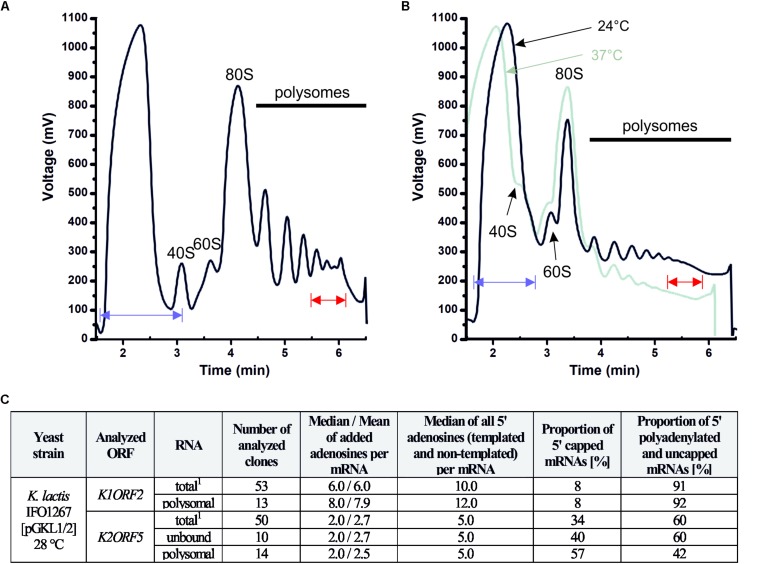
The 5′ polyadenylated and uncapped pGKL mRNAs are loaded onto yeast high polysomes. **(A)** Analysis of polysomes from *K. lactis* IFO1267 grown exponentially in YPD medium at 28°C. The fraction of high polysomes (depicted in red) and the non-polysomal fraction (unbound, depicted in blue) were used for the mRNA purification and 5′ RACE analysis of pGKL mRNAs. **(B)** Analysis of polysomes from the *S. cerevisiae* CWO4 *cdc33-42*ρ^0^ [pGKL1/2] strain grown in YPD medium in parallel at permissive (24°C, black curve) and restrictive (37°C, green curve) temperatures. Overall cellular translation was substantially decreased in the absence of functional eIF4E (green curve). The fractions of high polysomes (depicted in red) and non-polysomal fractions (depicted in blue) from cultures grown at the permissive (24°C) and non-permissive (37°C) temperatures were used for the total RNA purification and 5′ RACE analysis of pGKL mRNAs (Figures 12, 13). **(C)** Tabular summary of the 5′ RACE results from mRNAs purified from unbound and high polysome fractions in the experiment “A”. Messenger RNAs of each ORF subjected to 5′ RACE analysis are characterized by the following data: (1) ORF name; (2) source of purified mRNA; (3) total number of analyzed clones; (4) median and mean of 5′ added adenosines per mRNA molecule; (5) median of all 5′ adenosines (templated and non-templated) per mRNA molecule; (6) proportion of 5′ capped mRNAs [in %]; (7) proportion of 5′ polyadenylated and uncapped mRNAs [in %]. 40S, eukaryotic small ribosomal subunit; 60S, eukaryotic large ribosomal subunit; 80S, eukaryotic ribosome (monosome).

**FIGURE 12:**
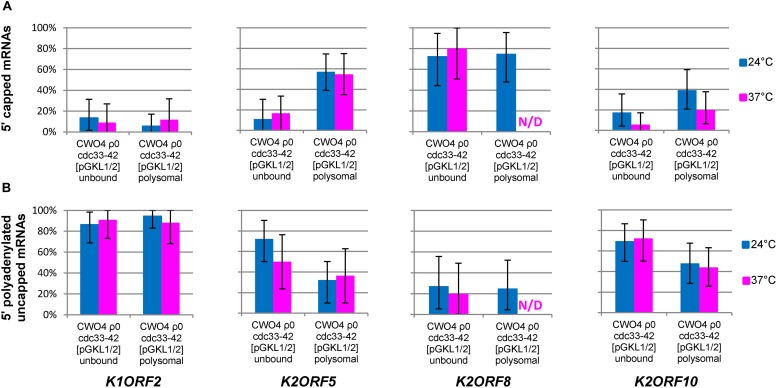
5′ polyadenylated and uncapped mRNAs associate with high polysomal fractions independently of cap-binding eIF4E. Bar plots depict the results of the 5′ RACE analyses of *K1ORF2*, *K2ORF5*, *K2ORF8*, and *K2ORF10* mRNAs from non-polysomal (unbound) and polysomal fractions of the *S. cerevisiae* CWO4 *cdc33-42*ρ^0^ [pGKL1/2] strain cultured at the permissive (24°C, blue bars) and non-permissive (37°C, magenta bars) temperatures. The cap-binding translation initiation factor eIF4E was not functional at the non-permissive temperature (Figure 11). **(A)** Proportions of 5′ capped mRNAs [in %] outside the polysomes (unbound) and in the high polysomal fraction (polysomal); **(B)** proportion of 5′ polyadenylated (and uncapped) mRNAs [in %] outside the polysomes (unbound) and in the high polysomal fraction (polysomal). Error bars depict the 95% confidence intervals calculated using the adjusted Wald method.

**FIGURE 13:**
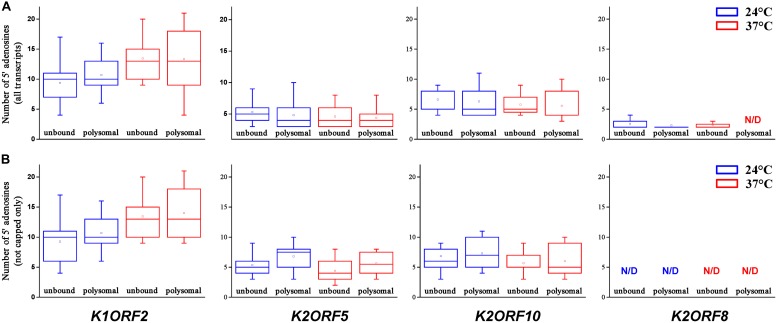
Analysis of the 5′ poly(A) leaders of selected pGKL mRNAs in non-polysomal (unbound) and polysomal fractions of the *S. cerevisiae* CWO4 *cdc33-42*ρ^0^ [pGKL1/2] strain cultured at the permissive (24°C, blue bars) and non-permissive (37°C, red bars) temperatures (Figure 11). The box whisker plot represents the number of templated and non-templated consecutive adenosine nucleotides at the 5′ ends of the indicated pGKL mRNAs. The bottom and top of the box indicate the first and third quartiles, respectively. The whiskers indicate the 10th and 90th percentiles. The median is depicted as a solid line, and the mean is depicted as a small square within the box. Outliers are not shown. **(A)** Lengths of the 5′ poly(A) leaders, including their templated and non-templated consecutive adenosine nucleotides calculated for all 5′ polyadenylated transcripts. **(B)** Lengths of the 5′ poly(A) leaders, including their templated and non-templated consecutive adenosine nucleotides calculated for the group of the 5′ polyadenylated and uncapped transcripts only. In total, 159 sequences obtained by 5′ RACE PCR were used for this analysis (Supplementary Table S13). ND, not detected.

To investigate whether 5′ polyadenylated mRNAs, which do not bear 5′ caps or 3′ poly(A) tails, are actively translated, we analyzed their possible presence with actively translating polysomes. Polysome profile analysis was performed as described previously (Masek et al., 2011; Pospisek and Valasek, 2013). We found that these mRNAs were present in polysomes in both the yeast *K. lactis* IFO1267 naturally bearing pGKL1/2 VLEs and the engineered *S. cerevisiae* CWO4 *cdc33-42*ρ^0^ [pGKL1/2] strain (Figures 11–13). The latter allowed us to study the impact of the length of 5′ poly(A) leaders on the mRNA load onto polysomes at both the permissive and elevated temperatures when eIF4E-dependent translation initiation was impaired.

Figures 11A,C clearly shows that uncapped and 5′ polyadenylated mRNAs are readily present in *K. lactis* polysomes under normal growth conditions. The proportion of 5′ m^7^G capped *K1ORF2* mRNAs coding for α/β toxin subunits in *K. lactis* polysomes mirrored their proportion in the total RNA preparation and corresponded to ∼8% of the sequenced *K1ORF2* mRNA UTRs in both experiments. Likewise, the proportions of 5′ polyadenylated and uncapped *K1ORF2* mRNAs were almost the same in the total RNA and high-polysome fractions and corresponded to 91 and 92%, respectively.

In the case of *K2ORF5* mRNA, the occurrence of capped mRNAs was slightly higher in *K. lactis* polysomes than in the total mRNA and represented 57 and 34% of the sequenced *K2ORF5* mRNA 5′ UTRs, respectively (Figure 11C and Supplementary Tables S4, S13). The proportion of capped *K2ORF5* mRNAs in the non-polysomal fraction (unbound) was 40%, which was similar to their proportion in the total RNA preparation. Although a slight enrichment of 5′ capped mRNAs was visible, this difference was not statistically significant. Similarly, proportions of 5′ polyadenylated and uncapped *K2ORF5* mRNAs were identical in the total RNA and non-polysomal fractions. A slight but not statistically significant decrease in such mRNAs was recognizable in polysomes (Figure 11C).

The *S. cerevisiae* CWO4 *cdc33-42*ρ^0^ [pGKL1/2] strain cultured at 37°C for 6 h typically exhibited severely decreased polysomes (Feketova et al., 2010) that nevertheless contained both capped and uncapped pGKL VLE mRNAs (Figures 11B, 12, 13). Regardless of the low mRNA content in the affected polysomes, we successfully performed 5′ RACE analyses of mRNAs corresponding to four pGKL1/2 genes. The overall ratio of mRNAs containing 5′ poly(A) leaders only and m^7^G caps exhibited the same trend as that in the *K. lactis* total RNA preparation regardless of whether the cultivation was performed at permissive or non-permissive temperatures. That means, *K1ORF2* mRNAs contained long 5′ poly(A) leaders and were predominantly not capped, *K2ORF5* mRNAs were 5′ capped at a medium level and polyadenylated, *K2ORF10* mRNAs exhibited frequently occurring medium-length 5′ poly(A) leaders and a low occurrence of 5′ capping, and, finally, *K2ORF8* mRNAs showed a high occurrence of 5′ capping and a low occurrence of short 5′ poly(A) leaders. Some differences did exist between the results of the 5′ RACE analyses of pGKL VLE mRNAs from the *K. lactis* and *S. cerevisiae* hosts, and these differences may have been either host specific or attributed to experimental variations in yeast cultures. Namely, we could not find any 5′ capped *K2ORF10* mRNAs in *K. lactis* IFO1267, whereas we found some in the *S. cerevisiae* CWO4 *cdc33-42*ρ^0^ [pGKL1/2] strain. Additionally, the occurrence of 5′ polyadenylation of *K2ORF8* mRNA was much lower in *S. cerevisiae* than in *K. lactis.* However, the average length of non-templated *K2ORF8* 5′ poly(A) leaders remained very low in both hosts (≤1 adenosine residue) (Supplementary Tables S4, S13). Altogether, 5′ RACE analysis revealed similar organization of pGKL-specific 5′ mRNA ends in both species.

The main purpose of the polysome profiling/5′ RACE experiment with the *S. cerevisiae* CWO4 *cdc33-42*ρ^0^ [pGKL1/2] strain was to investigate the possible impact of eIF4E conditional knockdown on the load of pGKL mRNAs onto polysomes. At the permissive 24°C temperature, we observed identical proportions of 5′ capped mRNAs in polysomal and non-polysomal fractions of toxin-encoding *K1ORF2* (highly 5′ polyadenylated) and *K2ORF8* [highly 5′ capped, short 5′ poly(A) leaders] transcripts (Figures 11, 12 and Supplementary Table S13). However, *K2ORF5* and, to some extent, *K2ORF10* exhibited higher incidences of 5′ capped mRNAs in high polysomes than in the unbound fractions. Both *K2ORF5* and *K2ORF10* mRNAs belong to the group with medium length poly(A) leaders (Figure 13 and Supplementary Table S13). Interestingly, the proportions of 5′ capped and 5′ polyadenylated transcripts between high polysomes and unbound fractions did not change significantly at the restrictive temperature, i.e., in the absence of the functional cap-binding protein eIF4E. The only exception was *K2ORF8* mRNAs, which were predominantly 5′ capped and contained a very short 5′ poly(A) leader. We could not detect any *K2ORF8* mRNAs in high polysomes at the restrictive temperature of 37°C. Differences in *K2ORF8* transcript occurrence should not be attributed to a low level of transcription because they were readily present in the unbound fraction at both the permissive and restrictive temperatures (Figures 11–13).

Moreover, the strengths of several pGKL1/2 promoters were determined previously by Schickel et al. (1996) who showed that the strengths of the *K2ORF8* and *K2ORF5* promoters are almost equal, whereas the *K1ORF2* promoter is even ≈2.5 times weaker than both the *K2ORF8* and *K2ORF5* promoters.

Next, we wanted to determine whether differences existed between unbound fractions and high polysome fractions in the lengths of 5′ poly(A) leaders belonging to transcripts of the same VLE gene. The analysis showed slight but recognizable enrichment of transcripts with longer poly(A) leaders in high polysomes within a group of 5′ uncapped and 5′ polyadenylated transcripts. This phenomenon was most profound among *K2ORF5* mRNAs (Figure 13B). Interestingly, cultivation of *S. cerevisiae* CWO4 *cdc33-42*ρ^0^ [pGKL1/2] at the non-permissive 37°C temperature led to the lengthening of *K1ORF2* 5′ poly(A) leaders (Figure 13).

Collectively, our results strongly suggest (i) that loading of pGKL1/2 mRNAs onto polysomes is mediated by both 5′ poly(A) leaders and m^7^G caps depending on the length of the 5′ consecutive adenosine nucleotide stretch and (ii) that pGKL1/2 mRNAs bearing longer 5′ poly(A) leaders, exemplified herein by *K1ORF2*, *K2ORF5*, and *K2ORF10* transcripts, can be loaded onto high polysomes and thus probably translated in an eIF4E-independent manner regardless of whether they contain a 5′ m^7^G cap. Furthermore, our results (iii) indicate that pGKL mRNAs bearing very short 5′ poly(A) leaders of 2–3 nt (*K2ORF8*) require functional eIF4E to be utilized by the translational machinery and (iv) explain the production of the killer toxin coded by *K1ORF2* and *K1ORF4* in the absence of functional eIF4E.

We cannot exclude the possibility that the enrichment of 5′ cap-containing *K2ORF5* and *K2ORF10* mRNAs in high-polysome fractions at the permissive and restrictive temperatures was caused by higher stability of these transcripts in comparison to those of the uncapped ones or even by another unknown mechanism of 5′ cap recognition by yeast translation initiation machinery. However, the latter is less probable because the *K2ORF8* transcripts were eliminated from polysomes when the cap-binding eukaryotic translation initiation factor 4E was impaired. Therefore, it is possible that the length of the 5′ poly(A) leader adjacent to the 5′ m^7^G cap might be important for translation initiation and polysome formation, even in the case of 5′ capped mRNAs.

Short uninterrupted 5′ poly(A) leaders have been recently reported to increase the translation efficiency of reporter mRNAs in cells infected with the vaccinia virus, including cells exhibiting decreased eIF4E levels mediated by siRNA knockdown (Dhungel et al., 2017). The average length of 5′ poly(A) leaders of intermediate and late mRNAs of the vaccinia virus is between 8 and 12 adenosine nucleotides (Yang et al., 2012). The 5′ poly(A) leaders of this length also appear to be most efficient in translation of the reporter mRNAs transfected into HeLa cells infected with the vaccinia virus, whereas 5′ leaders comprising only four consecutive adenosines appear to be translationally inefficient (Dhungel et al., 2017).

### Pab1 and Lsm1 Deletions Support the Expression of Toxin-Encoding pGKL Genes

Poly(A) tracts within the 5′ UTRs and 5′ poly(A) leaders of eukaryotic and viral mRNAs have been identified as enhancers of mRNA translation in various examples (Gudkov et al., 2005; Gilbert et al., 2007; Shirokikh and Spirin, 2008; Tahiri-Alaoui et al., 2014) as well as translational repressors in other cases (Melo et al., 2003; Patel et al., 2005). Some studies even show that poly(A) stretches within 5′ UTRs may serve as translational enhancers until they reach a certain threshold length. A further increase in the number of consecutive adenosine nucleotides beyond the threshold length leads to translational repression (Xia et al., 2011). Poly(A)-binding protein 1 (PABP1) plays an important role in many of these studies and biological examples. PABP1 is an abundant general translation initiation factor that binds to 3′ poly(A) tails, a common mark of most eukaryotic mRNAs (Sachs et al., 1986). In addition, PABP1 serves as a specific regulatory protein and has an important role in mRNA stability and turnover. The importance of PABP1 in cellular translation and other vital processes is underlined by increasing evidence that it is targeted by a wide range of viruses aiming to overtake host protein synthesis or escape the cellular antiviral defense (Kahvejian et al., 2005; Smith and Gray, 2010; Kobayashi et al., 2012; Costello et al., 2015).

To investigate the possible effects of yeast PABP1 protein (Pab1) on the expression of pGKL genes, we deleted the *PAB1* gene from *K. lactis*. Because no experimental data describing *PAB1* deletion in the yeast *K. lactis* were available, we followed a strategy used for the deletion of *PAB1* in the yeast *S. cerevisiae* (Mangus et al., 1998). Deletion of the *PAB1* gene is lethal in *S. cerevisiae* (Sachs et al., 1987; Giaever et al., 2002), but this lethality can be suppressed by simultaneous deletion of the gene coding for Pab1-binding protein (Pbp1) (Mangus et al., 1998). We disrupted the *PBP1* gene in the chromosomal DNA of the *K. lactis* IFO1267 strain using a loxP-*G418*-loxP system (Guldener et al., 1996) and obtained the *K. lactis* IFO1267 *pbp1*::*G418* strain. The *G418* cassette was removed from the genome using an artificially produced Cre-recombinase, providing the strain *K. lactis* IFO1267 *pbp1*Δ. We used the same strategy to introduce a *PAB1* deletion and to obtain the required strain, *K. lactis* IFO1267 *pbp1*Δ *pab1*Δ.

**FIGURE 14:**
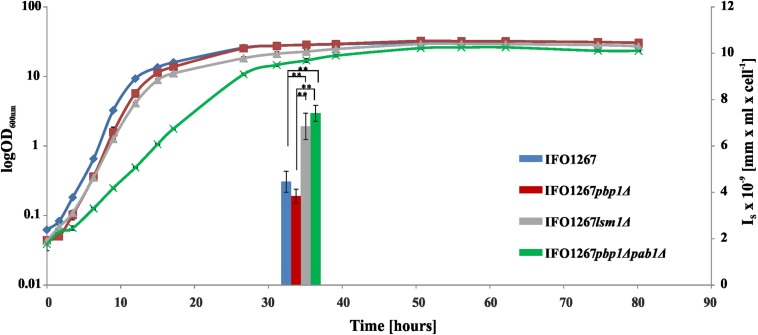
Production of the *K. lactis* killer toxin is enhanced in *LSM1* and *PAB1* deletion strains. Growth curves and production of the pGKL1 killer toxin from the *K. lactis* IFO1267 (blue), IFO1267 *pbp*1Δ (red), IFO1267 *lsm1*Δ (gray), and IFO1267 *pbp1*Δ *pab1*Δ (green) strains. All strains were grown in YPD medium at 28°C for approximately 80 h, and their OD_600 nm_ and toxin production were monitored. Production of the pGKL1 killer toxin in culture medium was assayed by a well diffusion test on YPD agar plates with a lawn of the *S. cerevisiae* S6/1 sensitive strain (for details of the method, refer to section Materials and Methods and Supplementary Figure S4). The toxin levels in culture media in the early stationary phase (∼35 h), when all the cultures reached a comparable OD_600_, are depicted. The amount of active toxin produced by each strain is presented as the calculated normalized inhibition zone at killer toxin saturation (*I*_s_). Growth curves and *I*_s_ determination correspond to at least three independent replicates. The results were statistically evaluated using one-way ANOVA followed by *post hoc* Tukey’s HSD test and further confirmed with Scheffé multiple comparison (^∗∗^*p* < 0.01). Normal distribution of the data was confirmed by the Shapiro–Wilk test. Error bars at each point on the growth curves and in the bar graph represent standard deviation.

Another protein that stabilizes some viral 5′ poly(A) tracts and may thus also affect translation of the respective mRNAs is Lsm1 (Bergman et al., 2007). We disrupted the *LSM1* gene in the *K. lactis* IFO1267 genome using a strategy similar to that described for *PBP1* deletion. The *K. lactis pbp1*Δ *pab1*Δ double-mutant strain is viable but exhibits a slow-growth phenotype, similar to the behavior reported for *S. cerevisiae pbp1*Δ *pab1*Δ (Mangus et al., 1998). Deletions of *LSM1* and *PBP1* from *S. cerevisiae* decrease growth rates (Mayes et al., 1999; Yoshikawa et al., 2011). In contrast, we detected only a minor decrease in the growth rate of the *K. lactis lsm1*Δ strain and no difference in the growth rate of the *K. lactis pbp1*Δ strain from the growth rate of their parental wild-type strain *K. lactis* IFO1267 (Figure 14).

To detect possible effects of the deleted proteins on the expression of pGKL mRNAs, we measured *K. lactis* killer toxin production. Production of the toxin in the culture medium was quantified by a well test on the sensitive yeast strain lawn. Using a serial dilution of the toxin-containing culture medium and a calibrated digital microscope to measure the width of the inhibition zone, we substantially improved the range, accuracy, and reproducibility of the killer toxin well test. The relationship between the killer toxin concentration and its activity, calculated as the width of the inhibition zone normalized to the concentration of production cells *(I)*, yields a hyperbolic function, the limit of which corresponds to the calculated theoretical width of the normalized inhibition zone at killer toxin saturation (*I*_s_) (see section Materials and Methods and Supplementary Figure S4).

We calculated *I*_s_ for the toxin activity present in the culture media of all the strains after 35 h of cultivation. Disruption of *LSM1* and *PAB1*, but not *PBP1*, enhanced the production of the toxin encoded by the linear cytoplasmic pGKL1 VLE (Figure 14 and Supplementary Figure S4).

The Lsm1 protein is part of the complex of cellular mRNA decapping activators employed by positive-strand RNA viruses to promote their translation and replication (reviewed in Jungfleisch et al., 2016) and might stabilize orthopoxviral mRNAs containing 5′ poly(A) leaders (Bergman et al., 2007). Despite many similarities between poxviral and pGKL-encoded transcription and RNA-modification apparatuses (Jeske et al., 2007), the simplest explanation for the increased pGKL toxin production in the *K. lactis lsm1*Δ strain might be the stabilization of pGKL mRNAs in the absence of Lsm1p due to prominent involvement of the Lsm1 protein in a cytoplasmic deadenylation-dependent mRNA decay pathway (reviewed in Parker, 2012).

We calculated the lengths of the continuous 5′ poly(A) leaders of pGKL mRNAs while considering both non-templated and template-coded adenosine nucleotides. Although the maximal length of the 5′ mRNA poly(A) leaders can reach 26 nucleotides, their average and median lengths are much lower. The median lengths of 5′ poly(A) leaders in transcripts from only two genes, *K1ORF3* and *K2ORF7*, are 12 adenosine nucleotides. Transcripts from *K1ORF1*, *K1ORF2*, and *K2ORF11* have 5′ poly(A) leaders with median lengths of 10 adenosine nucleotides. All other pGKL transcripts have shorter 5′ poly(A) leaders (Figure 4 and Supplementary Tables S4, S5A).

Our results agree very well with the genome-wide study of Xia et al. (2011) who showed that the presence of poly(A) stretches in the 5′ UTRs of *S. cerevisiae* transcripts positively correlates with enhanced protein synthesis until the length of the 5′ poly(A) regions reaches at least 12 consecutive adenosine nucleotides, which is also the optimal length for the binding site of yeast Pab1 (Sachs et al., 1987). Similarly, Dhungel et al. (2017) demonstrated that reporter mRNAs transfected into HeLa cells infected with vaccinia virus yielded the highest translation rate with 5′ leaders consisting of 8, 10, and 12 consecutive adenosine residues, where the 12(A) mRNA 5′ leader was the most efficient (Dhungel et al., 2017).

The Pab1 protein might function as a negative regulator that can, due to the documented sharp increase in its binding affinity to poly(A) stretches ranging from 8 to 12 adenosine residues in length (Sachs et al., 1987), further modulate the expression of pGKL genes. Such Pab1 activity was suggested for a larger set of yeast genes and can be demonstrated for cyclin *PCL5* coding mRNA, which contains the longest yeast 5′ UTR poly(A) region and shows inefficient translation and extremely low ribosomal occupancy (Xia et al., 2011). In mammalian cells, PABP1 binds to the adenosine-rich elements within the 5′ UTR of its own mRNA as a part of the negative autoregulatory complex (Patel et al., 2005). Messenger RNAs containing long uncapped poly(A) leaders (25 nt long) are efficiently translated into WGE lysates, and in contrast to other mRNAs, their translation rate does not decrease at high mRNA concentrations (Gudkov et al., 2005). This behavior suggests a low dependence of such mRNAs on translation initiation factors. Indeed, mRNAs containing long 5′ poly(A) leaders can efficiently enter translation, even in the absence of the otherwise essential translation factors eIF4F, eIF3, and PABP1 (Shirokikh and Spirin, 2008).

Late poxviral mRNAs containing long 5′ poly(A) leaders have a low requirement for eIF4F and perhaps also have a low requirement for PABP1 (Mulder et al., 1998; Xia et al., 2011; Dhungel et al., 2017). Interestingly, pGKL1 mRNAs coding for toxin and immunity phenotypes contain a higher fraction of mRNAs with 5′ poly(A) leaders that are at least 12 residues long (Figure 4 and Supplementary Tables S4, S5A). These mRNAs, which perhaps normally have Pab1 bound to their 5′ UTRs, may become unblocked and available for translation in the *K. lactis pbp1*Δ *pab1*Δ strain, thus increasing overall pGKL toxin production, as we observed. The control strain, *K. lactis pbp1*Δ, showed no detectable growth defects and/or enhancement of pGKL toxin production (Figure 14). To exclude the possibility of changes in the 5′ poly(A) leader length and m^7^G capping frequency of pGKL transcripts in the *pbp1*Δ *pab1*Δ double-mutant strain, we analyzed the 5′ ends of *K1ORF2* and *K1ORF3* mRNAs coding for the toxin α/β-subunit and immunity, respectively, by the 5′ RACE. As clearly seen in Supplementary Table S9, deletion of the *PAB1* gene does not remarkably affect the structure of the 5′ ends of the pGKL mRNAs in *K. lactis* cells.

We detected increased length of *K1ORF2* poly(A) leaders (median of 13 adenosines) in experiments with CWO4 *cdc33-42*ρ^0^ [pGKL1/2] cultivated at the non-permissive temperature of 37°C (Figure 13 and Supplementary Table S13). Pab1 is a physiological stress sensor that rapidly localizes to stress granules upon stress insult (Riback et al., 2017). Others have also shown that deficiencies in mitochondrial DNA decrease yeast tolerance to heat stress (Zubko and Zubko, 2014). We can speculate that the elevated temperature and stress induced by non-functional eIF4E could lead to the sequestration and decreased availability of Pab1 and thus to higher translatability and, consequently, stability of the toxin-coding uncapped and 5′ polyadenylated *K1ORF2* mRNAs.

## Conclusion

We employed pGKL VLEs from the yeast *K. lactis* as a model to investigate and present herein a complex view of the largely unexplored transcriptome of yeast cytoplasmic linear VLEs. Although previous experiments reported the binding of pGKL mRNAs to an oligo(dT) column (Stark et al., 1990), we showed that the pGKL transcripts are not polyadenylated at their 3′ ends; however, they frequently contain uncapped 5′ poly(A) leaders that are not complementary to the VLE genomic DNA and are probably synthesized by RNA polymerase slippage. The degree of 5′ capping and/or 5′ polyadenylation is specific to each gene and is controlled by the short UCR sequences preceding the corresponding AUG start codon. To our knowledge, this is the first example that the occurrence of 5′ mRNA capping is controlled by the promoter sequence. We also showed that the lengths of the 5′ poly(A) leaders of pGKL mRNAs inversely correlate with the proportion of 5′ mRNA capping. pGKL transcriptome analysis allowed us to refine the description of pGKL promoters and revealed new alternative promoters and corresponding TSSs for the *K1ORF4*, *K2ORF3*, and *K2ORF4* genes.

The presented data support the hypothesis that the translation of pGKL transcripts is independent of the eIF4E and Pab1 translation factors. With regard to our results and published data on yeast (Xia et al., 2011) and mammalian (Melo et al., 2003; Patel et al., 2005) mRNAs containing long poly(A) stretches in their 5′ UTRs, we suggest that Pab1 and Lsm1 serve as negative regulators of pGKL mRNAs containing 5′ poly(A) leaders. Further work will be necessary to determine whether differences in the median lengths of the 5′ mRNA poly(A) leaders of each gene (Supplementary Tables S4, S5A and Figure 4) reflect the degree of the corresponding gene expression inhibition mediated by Pab1 and whether this contributes as an evolutionary force driving the nucleotide sequence composition of individual UCRs. Our data are in good agreement with the average length of 8–12 adenosines in the 5′ poly(A) leaders of poxviral postreplicative mRNAs (Yang et al., 2012). 5′ poly(A) leaders of such lengths also maximally enhanced the translation of reporter mRNAs transfected into the HeLa cells infected with the vaccinia virus (Dhungel et al., 2017).

We hypothesize that the small and extremely compact genomes of pGKL VLEs use different levels of 5′ capping and 5′ polyadenylation of their transcripts to finely tune the expression of each gene to proper levels to facilitate the formation of functional protein complexes and to keep pGKL gene expression in harmony with the host cell. Initial evidence supporting this hypothesis came from a comparison of our data with that of Schickel et al. (1996) as discussed earlier, and from our analyses of the loading of pGKL mRNAs onto polysomes in normal growth conditions and in cells lacking the functional cap-binding translation initiation factor 4E. Our data further support a hypothesis about the evolutionary relationship between the family of cytoplasmic yeast linear VLEs and poxviruses (Jeske et al., 2007; Sýkora et al., 2018). This connection is fascinating because members of the *Poxviridae* family have been found in only vertebrates and arthropods, and some cause highly contagious and serious diseases in humans and animals. Parallel studies of yeast cytoplasmic linear VLEs and poxviruses may elucidate the origin of eukaryotic DNA viruses. pGKL VLEs could thus help us to better understand the basic processes and host cell–virus interactions that cannot be easily studied in mammalian systems.

## Data Availability Statement

All datasets generated for this study are included in the manuscript/Supplementary Files.

## Author Contributions

VV and MP devised the project and the main conceptual ideas. VV, MS, MP, and TM designed and performed the experiments. VV and MS created the figures. MP and TM received the funding. All authors analyzed the data, discussed the results, and contributed to the final manuscript.

## Conflict of Interest

The authors declare that the research was conducted in the absence of any commercial or financial relationships that could be construed as a potential conflict of interest.
