# Supplementary material for: Messenger RNAs of Yeast Virus-Like Elements Contain Non-templated 5′ Poly(A) Leaders, and Their Expression Is Independent of eIF4E and Pab1
Source: Front Microbiol. 2019 Oct 30;10:2366. doi: 10.3389/fmicb.2019.02366 (PMC6831550; doi:10.3389/fmicb.2019.02366)
Supplement: Supplementary file 1 [file Data_Sheet_1.pdf]

Messenger RNAs of Yeast Virus-Like Elements Contain Non-templated 5' Poly(A) Leaders, and Their Expression is Independent of eIF4E and Pab1

Václav Vopálenský, Michal Sýkora, Tomáš Mašek, Martin Pospíšek

SUPPLEMENTARY MATERIAL AND METHODS

Oligo-capping of pGKL mRNAs

Total yeast RNA was purified by the hot acidic phenol procedure (Lin et al., 1996). Remaining DNA was removed by a DNA-free Kit (Ambion) according to the manufacturer's protocol. The quality of RNA was assessed by electrophoresis according to the protocol by Masek et al. (Masek et al., 2005). Oligo-capping was performed using FirstChoice® RLM-RACE Kit (Invitrogen) according to the manufacturer's protocol. In brief, 5 µg of total yeast RNA was incubated with calf intestine alkaline phosphatase for 1 hour at 37°C; purified using acidic phenol/chloroform extraction and precipitated using ammonium acetate and isopropanol. Precipitated RNA was washed with 70% ethanol, resuspended in 1x tobacco acid pyrophosphatase buffer, and incubated with tobacco acid pyrophosphatase (TAP) for 1 hour at 37°C. A control reaction without the presence of TAP was also carried. After incubation 0.5 µl of 5' RACE Adapter, T4 RNA ligase buffer, and 2.5 U of T4 RNA ligase were added to the mixture, which was then incubated for 1 hour at 37°C. Reverse transcription was performed using 0.15 µg of random primers (Invitrogen) and 100 U of SSC III Reverse Transcriptase (Invitrogen) in a 20 µl reaction (25°C for 10 min, 50°C for 99 min, 70°C for 15 min). After reverse transcription, the cDNA was purified using the High Pure PCR Product Purification Kit (Roche). For amplification of cDNA, 2.5 µl of the reaction mixture was used for the following PCR with the 5' RACE Outer Primer and 5RACE_O8_K2 primer (5 min at 95°C; then 35 cycles of 30 sec at 94°C, 30 sec at 55°C, and 30 sec at 72°C; and finally, 10 min at 72°C). After amplification, 1 µl of the PCR mixture was used as a template for semi-nested PCR using 5' RACE Inner Primer and 5RACE_O8_K2 primer (5 min at 95°C; then 35 cycles of 30 sec at 94°C, 30 sec at 55°C, and 30 sec at 72°C; and finally, 10 min at 72°C). The corresponding fragment was purified from gel using a FastBack DNA minispin kit (Renogen Biolab), cloned into a pCR4-TOPO plasmid using the TOPO strategy and sequenced using the universal T7 promoter primer. All primers used in this study are listed in Table S1.

Quantification of mRNA abundances using qRT-PCR

Total yeast RNA was isolated from *K. lactis* IFO1267 from the late exponential growth phase (OD_{600} app. 2.8) as described previously. After DNA removal using DNase I (Ambion), cDNA was synthesized using SuperScript III Reverse Transcriptase (Invitrogen; RT+ reaction) and diluted 50 times. Control reaction, containing all components of the reverse transcription reaction except for the SSCIII enzyme (RT-), was also done. Two and half microliters of diluted RT reaction were subjected to Real-Time PCR amplification. qRT-PCR experiment was performed using LightCycler®480 instrument (Roche) and LightCycler®480 SYBR Green I Master (Roche). The 10 µl reactions were prepared in triplicates; each individual reaction contained 2.5 µl of 50 times diluted cDNA; 500 nM primers (see Table S1) and 5 µl of LightCycler®480 SYBR Green I Master. After amplification (5 min at 95°C;

then 45 cycles of 15 sec at 95°C, 20 sec at 55°C, and 30 sec at 72°C) relative quantification was applied to calculate Cp value for each gene analyzed. For evaluation of obtained results, semi-quantitave PCR reaction (5 min at 95°C; then 32 cycles of 30 sec at 95°C, 30 sec at 55°C, and 30 sec at 68°C; and finally, 10 min at 68°C) was performed with 2.5 µl of 50 times diluted cDNA (both RT+ and RT-) and the same primer combination as mentioned above. After amplification, 5 µl of the sample were analyzed using agarose gel electrophoresis (3% agarose in TAE buffer; 7 V/cm).

Statistical analyses and sample size estimation

Correlation of non-templated 5' mRNA poly(A) leader length and 5' mRNA capping frequency was analyzed using Pearson correlation coefficient. Variance of 5' mRNA poly(A) leader length of individual ORFs was analyzed using nonparametric Kruskal-Wallis test followed by *post hoc* Dunn test with p-value adjustment according to the Benjamini-Hochberg FDR method. This data did not follow normal distribution according to the Shapiro-Wilk test. Variance of non-templated 5' mRNA poly(A) leader length of ORFs with different number of template-coded consecutive 5' adenosines was analyzed using nonparametric Kruskal-Wallis test followed by *post hoc* Dunn test with p-value adjustment according to the Benjamini-Hochberg FDR method. This data did not follow normal distribution according to the Shapiro-Wilk test. Categorical binary data of 5' mRNA cap a 5'‘ poly(A) presence in pGKL transcripts were evaluated using two-tailed Fisher's exact test with 95% confidence interval. Variance of killer toxin activities of individual strains from at least three independent measurements was evaluated using one-way ANOVA followed by *post-hoc* Tukey’s HSD test with Scheffé multiple comparison. Normal distribution of the data was confirmed by Shapiro-Wilk test.

For results containing measurement variables and "*a priori*" calculation of the sample size we assumed normal distribution of data and one-way ANOVA tests. "*A priori*" samples size was computed with defined number of data groups and effect size (f), α error probability and statistical power (1-β) adjusted conventionally to 0.25, 0.05 and 0.8, respectively. This refers to experiments depicted in Figures 4, 6 and 14.

Data with binomial distribution (yes/no answer) were analyzed using a conservative Fisher's exact test. Pilot experiments were performed to obtain estimates of the P1 and P2 proportions. Values of α error probability and statistical power (1-β) were set conventionally to 0.05 and 0.8, respectively, for all the "*a priori*" calculations of sample size of groups 1 and 2 and total sample size in 2x2 contingency tables. Estimates of P1 and P2 were set to 0.2 and 0.6, respectively, for experiments depicted in Figure 7, and 0.2 and 0.8, respectively, for experiments depicted in Figure S2. Point estimates in bar plots were calculated using LaPlace method.

Actual sample sizes of the experiments can be found in the legends of the graphical interpretations of the experimental results or in the associated tables containing raw data or both. Actual sample sizes and statistical power were always reasonably higher than their computed "*a priori*" estimates.

All the sample size calculations were performed with G*Power 3 software (Faul et al., 2007).

SUPPLEMENTARY FIGURES AND TABLES

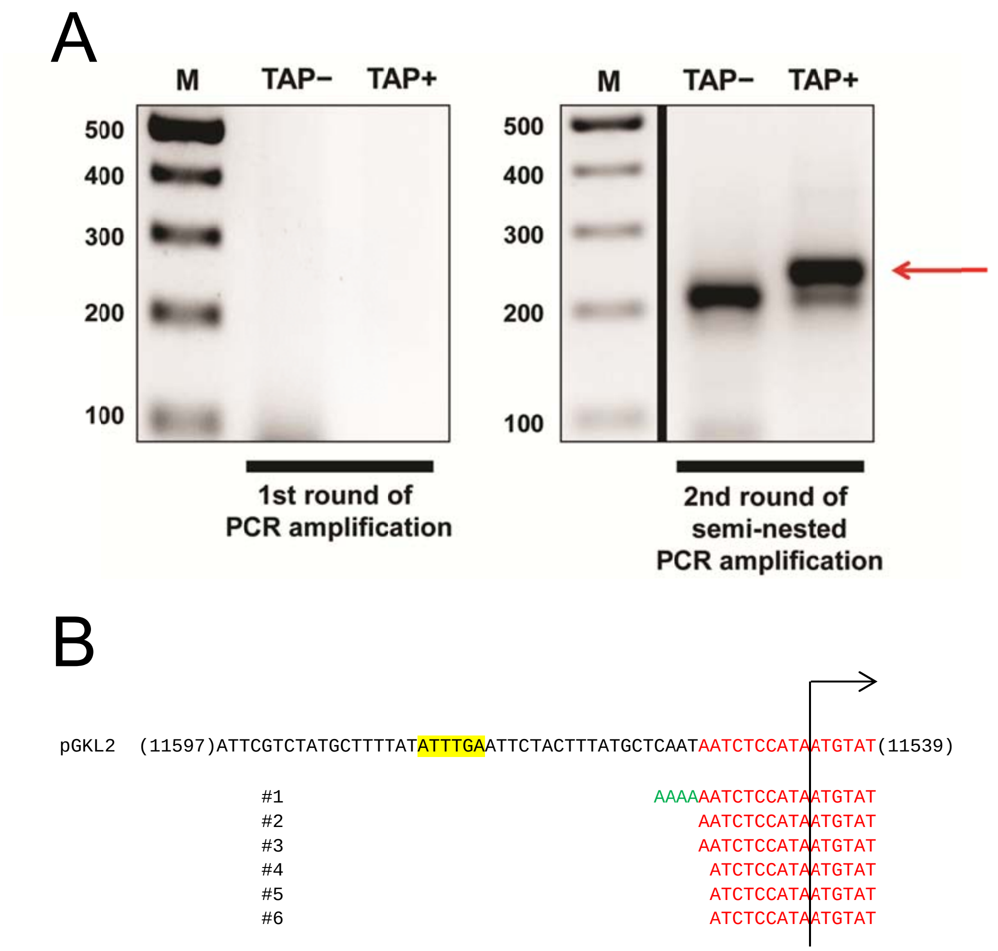

Figure S1. Transcripts of pGKL plasmids contain a 5' cap structure. (A) Electrophoretogram of *K2ORF8* mRNA oligo-capping analysis from *K. lactis* IFO1267 strain. Panel on the left displays electrophoretic analysis of the products obtained after first round of cDNA amplification using PCR. Panel on the right displays electrophoretic analysis of the products obtained after second round of cDNA amplification using semi-nested PCR and product of the first round PCR amplification as a template. The reactions in which the 5' dephosphorylated RNA was treated and not treated with tobacco acid pyrophosphatase prior RNA oligo ligation are labelled as TAP+ and TAP-, respectively. Specific product (~246 bp) corresponding to cDNA of *K2ORF8* 5' UTR is marked with red arrow. M: GeneRuler 100 bp Plus DNA Ladder (Thermo Scientific). (B) Oligo-capping analysis of the *K2ORF8* gene from *K. lactis* IFO1267 strain. In this panel, the upper template (plasmid) DNA sequence correspond to pGKL2 plasmid (GI: 2868) with the UCS sequence highlighted in yellow; sequences located below represent individual sequenced cDNA clones (the 5' untranslated region is displayed to the full extent until the translation start codon, ATG). The graphical representation of the oligo-capping results is similar to that in the Table S5A.

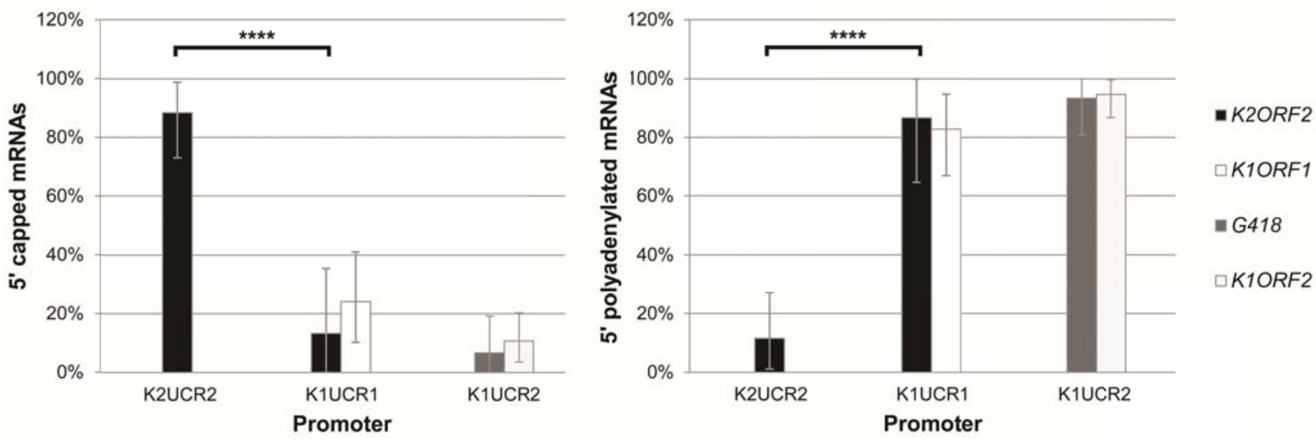

Figure S2. Promoters determine the degree of 5' capping and 5' polyadenylation of pGKL mRNAs. Analysis of 5' ends of pRKL2-1 plasmids revealed that presence of both non-templated adenosine addition and guanosine cap in pGKL-encoded mRNAs is directed by the linear plasmid promoters. Bars represent the frequency (in %) of 5' mRNA capping and polyadenylation with the error bars depicting the 95% confidence intervals calculated using the adjusted Wald method. Results were statistically evaluated using two-sided Fisher's exact test with 95% confidence interval. ****: significance level $p < 0.0001$. *K2ORF2* controlled by the *K1UCR1* promoter produces less frequently capped transcripts with a higher level of non-template 5' polyadenylation, similar to wild-type *K1ORF1* transcripts, and in contrast to *K2ORF2* transcripts controlled by the natural *K2UCR2* promoter, where the occurrence of 5' cap and 5' polyadenylation is significantly higher and lower, respectively. When *K1UCR2* promoter was used for expression of either its own gene (*K1ORF2*), or a heterologous bacterial gene coding for aminoglycoside 3'-phosphotransferase (*G418*), similar degree of both 5' mRNA capping and non-template polyadenylation occurred on the corresponding transcripts.

A

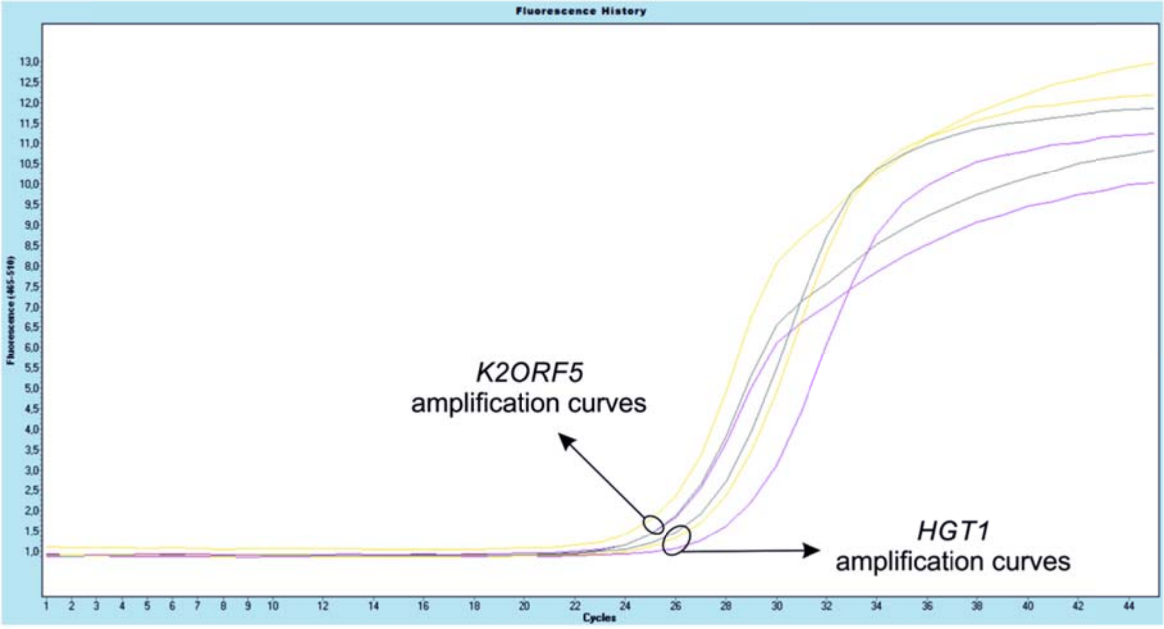

B

gene	replicate	Cp	Mean Cp	STD Cp
K2ORF5	1	25,22	25,20	0,09
	2	25,28		
	3	25,09		
HGT1	1	28,51	27,65	0,77
	2	27		
	3	26,15		

C

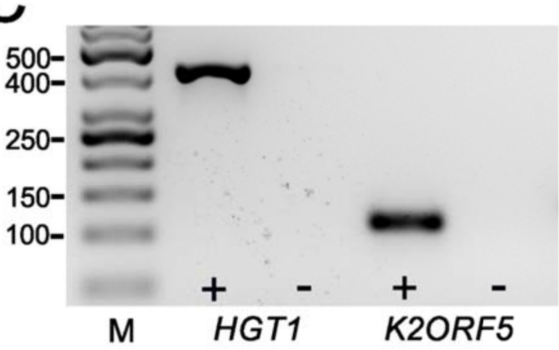

Figure S3. Validation of *K2ORF5* and *HGT1* mRNA abundances in the *K. lactis* IFO1267 total RNA. (A) Total RNA was isolated from *K. lactis* IFO1267 strain, DNase I-treated, reverse transcribed and subjected to the qRT-PCR analysis. The amplification curves of the *K2ORF5* and *HGT1* fragments demonstrate comparable amounts of *K2ORF5* and *HGT1* mRNAs in *K. lactis* cells in the late exponential growth phase. Amplification curves of the corresponding gene fragments are circled. The number of cycles is plotted on the x axis; relative fluorescence is depicted on the y axis. (B) Cp values calculated from Real-Time PCR experiment using LightCycler® 480 Software. Values are shown for each individual replicate; mean Cp value was also calculated from the individual measurements. (C) Semi-quantitative PCR analysis using agarose gel electrophoresis. 5µl of PCR reaction were analyzed using agarose gel electrophoresis; for each gene RT+ and RT- template cDNA was used. M - GeneRuler 50 bp DNA Ladder (Thermo Scientific); *HGT1+*/*K2ORF5+* - cDNA used as a template; *HGT1-* / *K2ORF5-* correspond to - RT reaction without reverse transcriptase that was used as a template (negative) control.

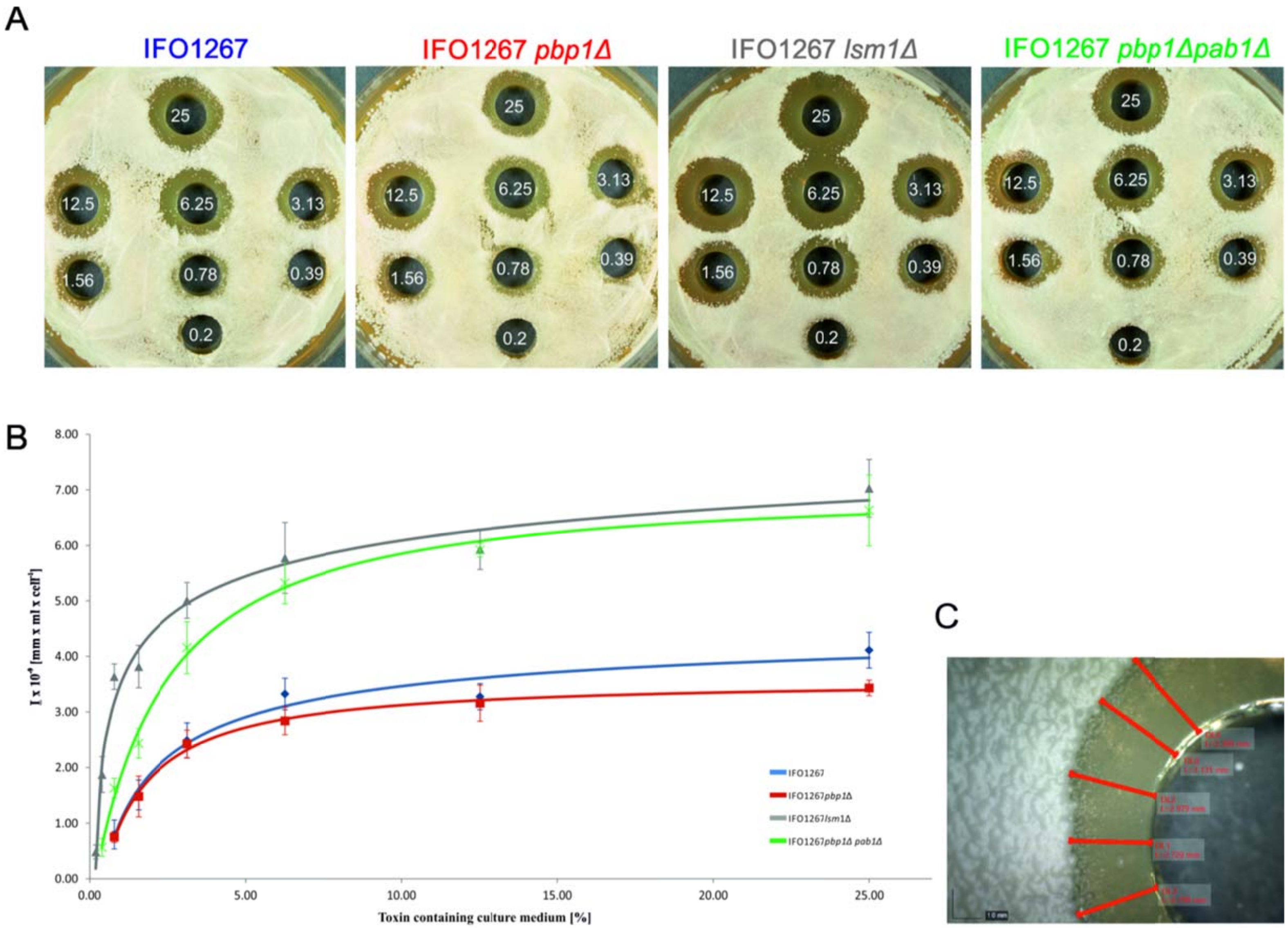

A
IFO1267
IFO1267 pbp1Δ
IFO1267 lsm1Δ
IFO1267 pbp1Δpab1Δ
25
12.5
6.25
3.13
1.56
0.78
0.39
0.2
B
I x 10⁻⁶ [mm x ml x cell⁻¹]
Toxin containing culture medium [%]
IFO1267
IFO1267pbp1Δ
IFO1267lsm1Δ
IFO1267pbp1Δ pab1Δ
C

Figure S4. Quantification of killer toxin production in *K. lactis* IFO1267, IFO1267 *pbp1Δ,* IFO1267 *lsm1Δ* and IFO1267 *pbp1Δ pab1Δ* strains. Toxin levels in a culture medium in late exponential phase (~35 hours) when all the cultures reached comparable OD_{600} (for the corresponding growth curve refer Figure 14), are depicted. (A) Production of the pGKL killer toxin into culture medium was assayed by a well diffusion test on YPD agar plates with a lawn of the *S. cerevisiae* S6/1 sensitive strain. Filter-sterilized samples were serial two-fold diluted; 100 μl of serially diluted toxin-containing culture medium were loaded into wells. The numbers in wells depict volume (in %) of the filter-sterilized culture medium diluted in YPD medium to reach the total volume of 100 μl loaded into the well. Wider inhibition zones corresponding to the higher toxin concentration are clearly visible in wells containing culture medium from the *lsm1Δ* strain and partly also from *pbp1Δpab1Δ* double-deletion strain. The *pbp1Δpab1Δ* strain displays slow growth in comparison to other strains tested (Figure 14) and its increased toxin production is better visible after normalization of data to the density of production cells. (B) Plot represents relationship between the width of the inhibition zone normalized to the concentration of the production cell (I; y axis) and a corresponding concentration of the diluted toxin-containing culture medium (%; x axis) used for the well inhibition assay as depicted on panel A. The curves can be described by hyperbolic functions, limits of which correspond to the calculated theoretical width of the normalized inhibition zone at killer toxin saturation (I_s). All killer tests and subsequent analyses were performed in triplicates. Error bars represent standard deviations. (C) Widths of the inhibition zones were measured using calibrated digital microscope after incubation of the YPD agar plates at 24°C for 48 hours. At least 20 independent measurements of the single inhibition zone were performed, the mean of these values was further normalised to the concentration of production cells and obtained values (I) were plotted (panel B).

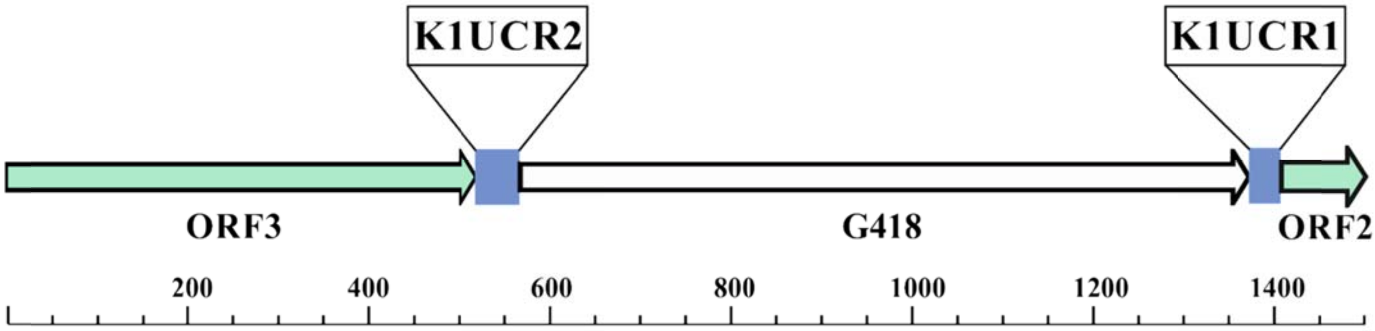

Figure S5. Precise manipulation of pGKL VLEs *in vivo* - general description of PCR cassette. PCR cassette used for the manipulation of pGKL2 by homologous recombination *in vivo* consists of regions homologous to the native pGKL2 VLE (in light green), an antibiotic resistance gene (*G418*) under the control of the *ORF2* promoter from pGKL1 (*K1UCR2*) and the *ORF1* promoter from pGKL1 (*K1UCR1*), which will artificially control the expression of *K2ORF2* (in light green). Promoters (UCRs) are in blue.

Table S1. Primers used in this study.

ORF /gene	primer name	Sequence (5'-3')	use in
K1ORF1	5RACE_O1_K1	CATGAAAGAAACTGATTGTCTAGAAAC	5' RACE
K1ORF2	5_RACE_02_K1	CCTTGACTCCATAATTTTGCAGCT	5' RACE
K1ORF3	5RACE_O3_K1_3	TAGGATACCAAATTCCTGAAGGC	5' RACE
K1ORF4	5_RACE_O4_K1	TCCATTAAATCCAGAGTTATTCTTTC	5' RACE
K2ORF1	5RACE_O1_K2	GTTGCATTATTGCAGCTTTAGC	5' RACE
K2ORF2	5RACE_O2_K2	TTCGTATGTAAATGTTTCCGCA	5' RACE
K2ORF3	vORF3-k2-rev_2	GTTCTTTTGTTAGCCGGTATT	5' RACE
K2ORF4	5RACE_O4_K2	ATCTAGAATCAAGAACAACTTTCTCA	5' RACE
K2ORF5	in ORF5 rev	GAGTAGTCTTTTCCGTATCCT	5' RACE / eIF4E binding assay / qPCR
K2ORF5	K2ORF5_qPCR_F1	TCTGACGGTTCTTTCAGAGC	qPCR
	K2ORF5_qPCR_R1	AGAGTAGTCTTTTCCGTATCCT	
K2ORF6	5RACE_O6_K2	CTGACCAATTTAATGGTAAATTCC	5' RACE
K2ORF7	5RACE_O7_K2	CAAATAGCTCATTTTTGTCATAAGC	5' RACE
K2ORF8	5_RACE_O8_K2	TCTTTTCAAAACTATCTAGCCACC	5' RACE / oligo-capping
K2ORF9	in_ORF9_rev	TGGAAATCTATTCATGTAAC	5' RACE
K2ORF10	5-RACEORF10k2	CTCATTTCTCTGTGTTTTGTT	5' RACE
K2ORF11	5RACE_O11_k2	ATAATCAGATAGTAACATCTCACCCTT	5' RACE
actin	actin_KL-rev	AACACCGTCACCAGAATCCAA	5' RACE
G418	in_Kan_rev1	GCAGTGGTGAGTAACCATGCA	5' RACE / *LSM1*/*PAB1*/*PBP1* deletion
universal	olig2(dC)anchor	GACCACGCGTATCGATGTCGACCCCCCCCCCCC	5' RACE
K1ORF1	ORF1-K1_tail_3	AGGATCAGAAGTAGGACAATTAGAAT	3' RACE
K2ORF10	K27	AATGGCTAATAAACAGGCAG	3' RACE
universal	oligo(dG)anch2	GATTGAGGTGTATCTGATGTCGAGGGGGGGGGGGG	3' RACE
universal	anch2	GATTGAGGTGTATCTGATGTCGA	3' RACE
universal	5' RACE Adapter	GCUGAUGGCGAUGAAUGAACACUGCGUUUGCUGG CUUUGAUGAAA	oligo-capping
universal	5' RACE Outer Primer	GCTGATGGCGATGAATGAACACTG	oligo-capping
universal	5' RACE Inner Primer	CGCGGATCCGAACACTGCGTTTGCTGGCTTTGATG	oligo-capping
HGT1	HGT1_KL-rev	TGACAACCGTAACCGATGTAG	eIF4E binding assay / qPCR
HGT1	HGT1_KL-forw	GTTCGGTTTTGATATCGCATC	eIF4E binding assay / qPCR
K2ORF5	in ORF5 forw	AGTGGTGAAGAGGAAAAATC	3' RACE / eIF4E binding assay / qPCR
eIF4E (*CDC33*)	eIF4Ef	CCACCATGGCCGTTGAAGAAGTTAGC	pGEX4T2::eIF4E construction
eIF4E (*CDC33*)	IF4Er	TGTGAAGCTTTTACAAGGTGATTGATGGTTG	pGEX4T2::eIF4E construction
KL_lsm1	KL_lsm1-del_For	TTTTTTTCACTTGCTCATTGAAAGAATCAGAGTCTCAA ATTACAACAGCTGAAGCTTCGTACGC	*LSM1* deletion
KL_lsm1	KL_lsm1-del_Rev	ACGATTTTTTTTTGCTTGTCTTTTAAACTAATTATAATC TTATACCGCATAGGCCACTAGTGGATCTG	*LSM1* deletion
KL_lsm1	lsm1_test_F	GTGGATCCTCAGCAGCTTTTTT	*LSM1* deletion
KL_lsm1	lsm1_test_R	GGTGAAAGAGGTGAAGGTTATCA	*LSM1* deletion
KL_pab1	KL_pab1-del_For	GCCATCTTTTTCGGTTAAATTTATCTCATCTCATCTCAT CTCATACAGCTGAAGCTTCGTACGC	*PAB1* deletion
KL_pab1	KL_pab1-del_Rev	AGATATAGAAAGGACAAGATGAAGATCAAGGTCAAA	*PAB1* deletion

		AAATCAAGTCGCATAGGCCACTAGTGGATCTG	
KL_pab1	pab1_test_F	TTACTGGCCAAGAGATGTCCC	*PAB1* deletion
KL_pab1	pab1_test_R	AGTTGGAAGACCCCATTTTCA	*PAB1* deletion
KL_pbp1	KL_pbp1-del_Rev	ACAAGTGGATACAATTGAATCCTTTAATGGTAGCCCT AATTCGAACGCATAGGCCACTAGTGGATCTG	*PBP1* deletion
KL_pbp1	KL_pbp1-del_For	AATTAGTAGCGACTCAGGTTTCTCTATACCAGGCTTC AGCAACCGCAGCTGAAGCTTCGTACGC	*PBP1 deletion*
KL_pbp1	pbp1_test_F_2	GATGGGCCTCTACAATGCAGAT	*PBP1 deletion*
KL_pbp1	pbp1_test_R_2	GATCGTTGTTATTGTCCGCAAA	PBP1 deletion
G418	KanV2F	GTTGTATTGATGTTGGACGAGTCGG	*LSM1*/*PAB1*/*PBP1* deletion
K2ORF3	ORF3_SAM_del_F1	ATTAAGTATGCTTCCTAAAACCACTTTATTGGCTATTG GGTCTGCAAAAGCTGGAGCTATAACTAAATGGACTA ATCTAA	promoter exchange
ORF3-pGKL2 (3' end) + ORF2-K1_5' UTR	ORF3_SAMdel_R1	AAAAACTTTCATATATTAAGTAGCTTTCACGGTCTCAT TTTTTAGAAAAGAAATGA	promoter exchange
ORF3-pGKL2 (3' end) + ORF2-K1_5' UTR	KL_orf6C_Flag2F	GACCGTGAAAGCTACTTAATATATGAAAGTTTTT	promoter exchange
G418 (3' end) + ORF1-pGKL1_5' UTR + ORF2-pGKL2 (5' end)	ORF3_SAMdel_R2	TTAAGAATGCTAATTCATCATTCATTTTTAAATTAATA ATGTAATTCATATATTATAGTTTAGAAAAACTCATCGA GCATCAAA	promoter exchange
K2ORF3	in_ORF3_forw	CTCTTGAAATAGAGTTTG	verification of plasmid modification
K2ORF3	K2_ORF3_for_seq	ATGAAGAATTTTCAGAAAGAAGTCC	verification of plasmid modification / sequencing

Restriction sites of the primers used for cloning with the restriction endonucleases are underlined.

Table S2. Yeast strains used in this study.

Yeast strain	Genotype	Reference / Source
K. lactis strains		
IFO1267	wild-type *MAT*a pGKL1^{+} pGKL2^{+}	Institute for Fermentation, Osaka
IFO1267 *pbp1*::*G418*	*MAT*a *pbp1*::*G418* pGKL1^{+} pGKL2^{+}	This study
IFO1267 *pbp1Δ*	*MAT*a *pbp1Δ* pGKL1^{+} pGKL2^{+}	This study
IFO1267 *pbp1Δ pab1Δ*	*MAT*a *pbp1Δ pab1*::*G418* pGKL1^{+} pGKL2^{+}	This study
IFO1267 *lsm1Δ*	*MAT*a *lsm1*::*G418* pGKL1^{+} pGKL2^{+}	This study
IFO1267_pRKL2-1	*MAT*a pGKL1^{+} pRKL2-1^{+} (K1UCR2-G418, K1UCR1-K2ORF2)	This study
IFO1267_pRKL1-1	pRKL1-1^{+} (K1UCR2-G418, orf2Δ) pGKL2^{+}	(Sýkora et al., 2018)
IFO1267_pRKL1-2	pRKL1-2^{+} (K1UCR2*-G418, orf2Δ) pGKL2^{+}	(Sýkora et al., 2018)
IFO1267_pRKL1-3	pRKL1-3^{+} (K1UCR2**-G418, orf2Δ) pGKL2^{+}	(Sýkora et al., 2018)
S. cerevisiae strains		
S6/1	*MAT*α	(Woods and Bevan, 1968)
YAT547	*MAT*α *leu2 trp1 cyh2 gal^{-} can1 kar1* ρ^{0} [dsRNA-L pGKL1^{+} pGKL2^{+}]	(Gunge and Yamane, 1984)
CWO4*CDC33*wt	*MAT*a *his3-11,15 ade2-1 ura3-1 trp1-1a leu2-3,112 can100 cdc33::LEU2* [CDC33wt TRP1 ARS CEN]	(Altmann et al., 1989)
CWO4*cdc33-1*	*MAT*a *his3-11,15 ade2-1 ura3-1 trp1-1a leu2-3,112 can100 cdc33::LEU2* [cdc33-1 TRP1 ARS CEN]	(Altmann et al., 1989)
CWO4*cdc33-42*	*MAT*a *his3-11,15 ade2-1 ura3-1 trp1-1a leu2-3,112 can100 cdc33::LEU2* [cdc33-42 TRP1 ARS CEN]	(Altmann et al., 1989)
CWO4ρ^{0}*CDC33*wt	*MAT*a *his3-11,15 ade2-1 ura3-1 trp1-1a leu2-3,112 can100 cdc33::LEU2* ρ^{0} [CDC33wt TRP1 ARS CEN]	This study
CWO4ρ^{0}*cdc*33-1	*MAT*a *his3-11,15 ade2-1 ura3-1 trp1-1a leu2-3,112 can100 cdc33::LEU2* ρ^{0} [cdc33-1 TRP1 ARS CEN]	This study
CWO4ρ^{0}*cdc*33-42	*MAT*a *his3-11,15 ade2-1 ura3-1 trp1-1a leu2-3,112 can100 cdc33::LEU2* ρ^{0} [cdc33-42 TRP1 ARS CEN]	This study
CWO4ρ^{0}*CDC33*wt[pGKL1/2]	*MAT*a *his3-11,15 ade2-1 ura3-1 trp1-1a leu2-3,112 can100 cdc33::LEU2* ρ^{0} [pGKL1^{+} pGKL2^{+}] [CDC33wt TRP1 ARS CEN]	This study
CWO4ρ^{0}*cdc33-1*[pGKL1/2]	*MAT*a *his3-11,15 ade2-1 ura3-1 trp1-1a leu2-3,112 can100 cdc33::LEU2* ρ^{0} [pGKL1^{+} pGKL2^{+}] [cdc33-1 TRP1 ARS CEN]	This study
CWO4ρ^{0}*cdc33-42*[pGKL1/2]	*MAT*a *his3-11,15 ade2-1 ura3-1 trp1-1a leu2-3,112 can100 cdc33::LEU2* ρ^{0} [pGKL1^{+} pGKL2^{+}] [cdc33-42 TRP1 ARS CEN]	This study
CWO4ρ^{0}*CDC33*wt[pYX212::M1]	*MAT*a *his3-11,15 ade2-1 ura3-1 trp1-1a leu2-3,112 can100 cdc33::LEU2* ρ^{0} [CDC33wt TRP1 ARS CEN] [M1 URA3 2μ ori]	This study

CWO4ρ^0*cdc33-1*[pYX212::M1]	*MAT*a *his3-11,15 ade2-1 ura3-1 trp1-1a leu2-3,112 can100 cdc33::LEU2* ρ^0 [pGKL1$^+$ pGKL2$^+$] [cdc33-1 TRP1 ARS CEN] [M1 URA3 2μ ori]	This study
CWO4ρ^0*cdc33-42*[pYX212::M1]	*MAT*a *his3-11,15 ade2-1 ura3-1 trp1-1a leu2-3,112 can100 cdc33::LEU2* ρ^0 [pGKL1$^+$ pGKL2$^+$] [cdc33-42 TRP1 ARS CEN] [M1 URA3 2μ ori]	This study

CEN-ARS, centromere-autonomous replicating sequence

*UCR sequence bearing one point mutation in putative initiator region (INR)

**UCR sequence bearing two point mutations in putative initiator region (INR)

Table S3. Plasmids used in this study.

Plasmid	Description	Reference
pGKL1	native, kill$^+$	(Gunge et al., 1981)
pGKL2	native	(Gunge et al., 1981)
pRKL1-1	modified pGKL1 containing G418 resistance marker under control of K1UCR2, deleted ORF2 gene	(Sýkora et al., 2018)
pRKL1-2	modified pGKL1 containing G418 resistance marker under control of K1UCR2 bearing one point mutation in putative initiator region, deleted ORF2 gene	(Sýkora et al., 2018)
pRKL1-3	modified pGKL1 containing G418 resistance marker under control of K1UCR2 bearing two point mutations in putative initiator region, deleted ORF2 gene	(Sýkora et al., 2018)
pRKL2-1	modified pGKL2 containing its own ORF2 under control of K1UCR1 and G418 resistance marker under control of K1UCR2	This study
pUG6	loxP-pAgTEF1-kanMX-tAgTEF1-loxP, G418 resistance, β-lactamase	(Guldener et al., 1996)
pSH65	pGAL1-cre, ARS CEN, pAgTEF1-ble-tScCYC1, Phleo resistance, URA3, β-lactamase	(Gueldener et al., 2002)
pYX212	2µ ori, URA3, β-lactamase, TPI promoter	Ingenius Inc.
pYX212::M1	pYX212 containing cDNA copy of killer virus M1	This study
pYX213::M1	pYX213 containing cDNA copy of killer virus M1	(Valis et al., 2006)
pCR4-TOPO	pLac, β-lactamase, aph(3')-II, ColE1, f1 ori	Invitrogen
pGEX-4T-2	N-GST; pTac; lacI, thrombin; β-lactamase	GE Healthcare
pGEX4T2::CDC33	pGEX-4T-2 containing yeast *S. cerevisiae* CDC33 gene produced as N-terminal GST fusion	This study

UCR - sequence located between AUG initiation codon and UCS (including) of the selected ORF

Table S4. Summary of 5' RACE experiments.

plasmid	ORF number	No. of 5' non-templated adenosines	Minimal number of adenosines[1] at 5' ends of uncapped transcripts	% of mRNAs with 5' non-templated poly(A) leaders	Median / mean of added adenosines per mRNA	No. of analyzed clones[2]	% of 5' capped mRNAs	5' UTR template complementarity[3]
pGKL1	**1**	1-10	5*	85.1	5.0 / 5.3	27 (6G+23A)	22.2	AAAAATG
	**2**	2-14	5*	96.2	6.0 / 6.0	53 (4G+51A)	7.5	AAAATG
	**3**	0-20	3*	98.2	7.0 / 6.7	55 (3G+54A)	5.3	AAAAATG
	**4**	0-16	6	87.0	6.0 / 5.8	23 (4G+20A)	17.4	AAAC-15nt**-ATG
pGKL2	**1**	0-10	2*	92.3	2.0 / 2.6	39 (19G+36A)	*48.7*	AAATG
	**2**	1	6*	8.3	0.0 / 0.8	24 (22G+2A)	91.7	AAAAAATG
	**3** **short**	1-3	5	40.0	0.0 / 0.7	10 (9G+4A)	90.0	AAC-57nt**-ATG
	**3** **long**	3	7	7.2	0.0 / 0.2	14 (13G+1A)	92.9	AAAAAG-144nt**-ATG
	**4**	0-21	4*	36.4	0.0 / 2.0	22 (12G+8A)	*54.5*	AAAAATG
	**5**	0-8	3	90.0	2.0 / 2.7	50 (17G+45A)	34.0	AAAG-81nt**-ATG
	**6**	1-10	7*	100.0	5.0 / 5.0	25 (5G+25A)	20.0	AAAATG
	**7**	0-19	4*	91.3	7.0 / 7.6	23 (1G+22A)	4.4	AAAAATG
	**8**	1-7	8	45.6	0.0 / 1.0	79 (68G+36A)	86.0	AAT-7nt**-ATG
	**9**	1-6	5	83.3	2.5 / 2.9	24 (8G+20A)	33.3	AAAC-19nt**-ATG
	**10**	0-16	3*	100.0	4.5 / 4.8	20 (0G+20A)	0.0	AAAATG
	**11**	2-10	5	100.0	6.5 / 6.1	20 (1G+20A)	5.0	AAACATG
**actin**	**N/A**	0	N/A	0.0	0.0	49	100.0	N/A

Each ORF is characterized by the following data: 1/ ORF number; 2/ number of 5' non-templated adenosine nucleotides found in the corresponding mRNAs; 3/ minimal number of adenosine nucleotides (calculated for both templated and non-templated adenosines) found at 5' ends of uncapped transcripts; 4/ percentage of all transcripts containing 5' non-templated adenosine nucleotides; 5/ the average number (shown as the mean and median) of non-templated adenosine nucleotides per mRNA molecule; 6/ number of sequenced clones used for analyses; the number of capped and 5' polyadenylated mRNAs are depicted below the total number; 7/ percentage of all transcripts containing 5' mRNA cap structure; 8/ part of the 5' UTR complementary to the relevant plasmid genome. Please note that columns 4 and 7 may not sum to one hundred percent because some transcripts contain both 5' non-templated adenosines and a cap structure. [1] templated and non-templated; [2] G = capped transcripts; A - 5' polyadenylated transcripts; [3] the first templated nucleotides transcribed to the mRNA, non-templated adenosines were removed; * these adenosines immediately precede the ATG sequence; ** for exact sequences, see Table S5A; transcripts from *K. lactis ACT* gene, coding for actin, were used as an internal control.

Table S5. Molecular analyses of plasmid specific mRNAs at the level of individual mRNA molecules.

A - The 5' untranslated regions of ORFs encoded by pGKL plasmids

pGKL1-ORF1

```
pGKL1   (183)TTTATTATACACATTTTCCAACTATAATATATGAATTACATTATTAATTTAAAAATGGAT(242)

                            #16                      AAAAAAAAAAAAAAATGGAT
                            #33                      AAAAAAAAAAAAAAATGGAT
                            #44                         AAAAAAAAAAAATGGAT
                            #4                          AAAAAAAAAAAATGGAT
                            #5                          AAAAAAAAAAAATGGAT
                            #40b                        AAAAAAAAAAAATGGAT
                            #39                          AAAAAAAAAAATGGAT
                            #15                          AAAAAAAAAAATGGAT
                            #37                           AAAAAAAAAATGGAT
                            #48A                          AAAAAAAAAATGGAT
                            #30                           AAAAAAAAAATGGAT
                            #23                           AAAAAAAAAATGGAT
                            #20                           AAAAAAAAAATGGAT
                            #48B                          AAAAAAAAAATGGAT
                            #36                            AAAAAAAAATGGAT
                            #47                            AAAAAAAAATGGAT
                            #31                            AAAAAAAAATGGAT
                            #48c                           GAAAAAAAATGGAT
                            #41T7                           GAAAAAATGGAT
                            #46                               AAAAAAATGGAT
                            #13                               AAAAAAATGGAT
                            #42A                              AAAAAAATGGAT
                            #41                                AAAAAATGGAT
                            #40                                 GAAAATGGAT
                            #32                                  GAAATGGAT
                            #46a                                  GATGGAT
                            #42b                                  GATGGAT
```

pGKL1-ORF2

Sequence	
pGKL1	(3181) AGACCGTGAAAGCTACTTAAT**ATATGA**AAGTTTTTATAATAATTATAAAATGAAT(3235)
A3b	AAAAAAAAAAAAAAAAAATGAAT
#40	AAAAAAAAAAAAAAAAATGAAT
A13	AAAAAAAAAAAAAAATGAAT
A6a	AAAAAAAAAAAAAAATGAAT
#7	AAAAAAAAAAAAAATGAAT
#12	AAAAAAAAAAAAAATGAAT
P3	AAAAAAAAAAAAAATGAAT
#11	AAAAAAAAAAAAATGAAT
#45	**G**AAAAAAAAAAAATGAAT
#13	AAAAAAAAAAAATGAAT
#N40	AAAAAAAAAAAATGAAT
#N41	AAAAAAAAAAAATGAAT
P4	AAAAAAAAAAAATGAAT
#15	AAAAAAAAAAATGAAT
A10	AAAAAAAAAAATGAAT
A5b	AAAAAAAAAAATGAAT
#39	AAAAAAAAAAATGAAT
#N38	AAAAAAAAAAATGAAT
A4	AAAAAAAAAAATGAAT
P8a	AAAAAAAAAAATGAAT
A1	AAAAAAAAAAATGAAT
A4b	**G**AAAAAAAAAATGAAT
#8	AAAAAAAAAATGAAT
A7b	AAAAAAAAAATGAAT
P7	AAAAAAAAAATGAAT
#34	AAAAAAAAAATGAAT
#35A	AAAAAAAAAATGAAT
A5	AAAAAAAAAATGAAT
A3a	AAAAAAAAAATGAAT
#N44	AAAAAAAAAATGAAT
#N39	AAAAAAAAAATGAAT
#38A	AAAAAAAAATGAAT
#46	AAAAAAAAATGAAT
A14	AAAAAAAAATGAAT
A8	AAAAAAAAATGAAT
A6b	AAAAAAAAATGAAT
A1a	AAAAAAAAATGAAT
A6	AAAAAAAAATGAAT
#41	AAAAAAAAATGAAT
P8	AAAAAAAAATGAAT
A2	AAAAAAAATGAAT
A17	AAAAAAAATGAAT
#6	AAAAAAATGAAT
A2b	AAAAAAATGAAT
#35	AAAAAAATGAAT
#36	AAAAAAATGAAT
#42	AAAAAAATGAAT
#38	AAAAAAATGAAT
A1b	AAAAAAATGAAT
A12	AAAAAATGAAT
A11	**G**AAAAATGAAT
#4	**G**AAAATGAAT
#44	**G**AAAATGAAT

pGKL1-ORF3

```
pGKL1 (7922)TAATGTTATTAGATAACAAACACTAAATATATGATATATCTTCATTTTAATTAAAAATGTGT(7983)

                     #17                    AAAAAAAAAAAAAAAAAAAAAAAAATGTGT
                     A21                       AAAAAAAAAAAAAAAAAAAAAATGTGT
                     A8p                       AAAAAAAAAAAAAAAAAAAAAATGTGT
                     #25b                         AAAAAAAAAAAAAAAAAATGTGT
                     A11                           AAAAAAAAAAAAAAAAAATGTGT
                     #32                            AAAAAAAAAAAAAAAAATGTGT
                     #26b                           AAAAAAAAAAAAAAAAATGTGT
                     #2                              AAAAAAAAAAAAAAAATGTGT
                     P18                             AAAAAAAAAAAAAAAATGTGT
                     #26                               AAAAAAAAAAAAAATGTGT
                     A23                               AAAAAAAAAAAAAATGTGT
                     P26                               AAAAAAAAAAAAAATGTGT
                     A12                               AAAAAAAAAAAAAATGTGT
                     A27                               AAAAAAAAAAAAAATGTGT
                     A34                               AAAAAAAAAAAAAATGTGT
                     A32                               AAAAAAAAAAAAAATGTGT
                     A38                                AAAAAAAAAAAAATGTGT
                     P15                                AAAAAAAAAAAAATGTGT
                     #12                                AAAAAAAAAAAAATGTGT
                     #14                                AAAAAAAAAAAAATGTGT
                     #16                                AAAAAAAAAAAAATGTGT
                     #30                                 AAAAAAAAAAAATGTGT
                     A29                                 AAAAAAAAAAAATGTGT
                     #18                                 AAAAAAAAAAAATGTGT
                     A19                                 AAAAAAAAAAAATGTGT
                     #20                                 AAAAAAAAAAAATGTGT
                     A15                                 AAAAAAAAAAAATGTGT
                     A14                                 AAAAAAAAAAAATGTGT
                     A10                                 AAAAAAAAAAAATGTGT
                     #25                                  AAAAAAAAAAATGTGT
                     A25                                  AAAAAAAAAAATGTGT
                     A35                                  AAAAAAAAAAATGTGT
                     #21                                  AAAAAAAAAAATGTGT
                     #29                                   AAAAAAAAAATGTGT
                     A22                                   AAAAAAAAAATGTGT
                     #22                                   GAAAAAAAAATGTGT
                     P25                                   GAAAAAAAAATGTGT
                     A13                                    AAAAAAAAATGTGT
                     #27                                    AAAAAAAAATGTGT
                     A37                                    AAAAAAAAATGTGT
                     A24                                    AAAAAAAAATGTGT
                     A30                                    AAAAAAAAATGTGT
                     A9                                     AAAAAAAAATGTGT
                     #33                                    AAAAAAAAATGTGT
                     P27                                    AAAAAAAAATGTGT
                     #31                                     AAAAAAAATGTGT
                     A31                                     AAAAAAAATGTGT
                     P28                                      AAAAAAATGTGT
                     #6                                       AAAAAAATGTGT
                     #28                                      AAAAAAATGTGT
                     P17                                       AAAAAATGTGT
                     #15                                       AAAAAATGTGT
                     #5                                        GAAAAATGTGT
                     #28A                                       AAAAATGTGT
                     #35                                        AAAAATGTGT
                     A36                                         AAAATGTGT
```

pGKL1-ORF4

```
pGKL1  (7854)TCATAATCTGAATAATAAGCATAAGTACATGCTTTAAAATAATCTGAAAGATTATTATCTAATTCTAAACACATTTTTAATTAAAATGAAG(7944)

#14    GATGCTTTAAAATAATCTGAAAGATTATTATCTAATTCTAAACACATTTTTAATTAAAATGAAG
#24    AAAAAAAAAAAAAAAAAAACACATTTTTAATTAAAATGAAG
#17A   AAAAAAAAAAAAACACATTTTTAATTAAAATGAAG
#18    AAAAAAAAAAAAACACATTTTTAATTAAAATGAAG
#12    AAAAAAAAAAACACATTTTTAATTAAAATGAAG
#20    AAAAAAAAAAACACATTTTTAATTAAAATGAAG
#21    AAAAAAAAAAACACATTTTTAATTAAAATGAAG
#27    AAAAAAAAAAACACATTTTTAATTAAAATGAAG
#13    AAAAAAAAAACACATTTTTAATTAAAATGAAG
#22    AAAAAAAAAACACATTTTTAATTAAAATGAAG
#7     AAAAAAAAACACATTTTTAATTAAAATGAAG
#N27   AAAAAAAAACACATTTTTAATTAAAATGAAG
#7A    AAAAAAAAACACATTTTTAATTAAAATGAAG
#10    AAAAAAAAACACATTTTTAATTAAAATGAAG
#N37   AAAAAAAAACACATTTTTAATTAAAATGAAG
#36    AAAAAAAAACACATTTTTAATTAAAATGAAG
#23A   AAAAAAACACATTTTTAATTAAAATGAAG
#29    AAAAAAACACATTTTTAATTAAAATGAAG
#N25   AAAAAAACACATTTTTAATTAAAATGAAG
#19    AAAAAACACATTTTTAATTAAAATGAAG
#15    GAAAAACACATTTTTAATTAAAATGAAG
#16    GAAACACATTTTTAATTAAAATGAAG
#23B   GACACATTTTTAATTAAAATGAAG
```

pGKL2-ORF1

```
pGKL2 (181)AAAATCAATTGAATGATTCTTAATATGATTTAATAGTTTATGATTATAAATGTCT(235)

#10    AAAAAAAAAAAAATGTCT
#13    AAAAAAAAATGTCT
#20    AAAAAAAAATGTCT
#25    AAAAAAAAATGTCT
#21    AAAAAAAATGTCT
#R16   GAAAAAAATGTCT
#14    AAAAAAATGTCT
#R21   AAAAAAATGTCT
#R27   AAAAAAATGTCT
#R23   AAAAAAATGTCT
#R26   AAAAAAATGTCT
#R17   AAAAAAATGTCT
#24    AAAAAAATGTCT
#9     GATAAAATGTCT
#R20   GATAAAATGTCT
#R29   AAAAAATGTCT
#7     GAAAAATGTCT
#8     GAAAAATGTCT
#R14   GAAAAATGTCT
#R13   GAAAAATGTCT
#18    GAAAAATGTCT
#19    GAAAAATGTCT
#22    GAAAAATGTCT
#15    GATAAATGTCT
#17    GATAAATGTCT
#4     AAAAATGTCT
#R22   AAAAATGTCT
#5     GAAAATGTCT
#12    GAAAATGTCT
#23    GAAAATGTCT
#R25   GAAAATGTCT
#11    AAAATGTCT
#27    AAAATGTCT
#R18   AAAATGTCT
#1     GAAATGTCT
#6     GAAATGTCT
#R15   GAAATGTCT
#R24   AAATGTCT
#2     AAATGTCT
```

pGKL2-ORF2

```
pGKL2  (2973)ATATGAAAATAAATAGAACCATGAATACCGGAAGTGCACAAGAAATAGAATTATCACGCATGTATTCTTATCATTTCTTTTCTAAAAAATGAAT(3880)

          #30                       GATACCGGAAGTGCACAAGAAATAGAATTATCACGCATGTATTCTTATCATTTCTTTTCTAAAAAATGAAT
          #R19                      GATACCGGAAGTGCACAAGAAATAGAATTATCACGCATGTATTCTTATCATTTCTTTTCTAAAAAATGAAT
          #11                         GACCGGAAGTGCACAAGAAATAGAATTATCACGCATGTATTCTTATCATTTCTTTTCTAAAAAATGAAT
          #14                         GACCGGAAGTGCACAAGAAATAGAATTATCACGCATGTATTCTTATCATTTCTTTTCTAAAAAATGAAT
          #47                         GACCGGAAGTGCACAAGAAATAGAATTATCACGCATGTATTCTTATCATTTCTTTTCTAAAAAATGAAT
          #37                         GACCGGAAGTGCACAAGAAATAGAATTATCACGCATGTATTCTTATCATTTCTTTTCTAAAAAATGAAT
          #36                         GACCGGAAGTGCACAAGAAATAGAATTATCACGCATGTATTCTTATCATTTCTTTTCTAAAAAATGAAT
          #15                         GACCGGAAGTGCACAAGAAATAGAATTATCACGCATGTATTCTTATCATTTCTTTTCTAAAAAATGAAT
          #33                         GACCGGAAGTGCACAAGAAATAGAATTATCACGCATGTATTCTTATCATTTCTTTTCTAAAAAATGAAT
          #17                         GACCGGAAGTGCACAAGAAATAGAATTATCACGCATGTATTCTTATCATTTCTTTTCTAAAAAATGAAT
          #18                         GACCGGAAGTGCACAAGAAATAGAATTATCACGCATGTATTCTTATCATTTCTTTTCTAAAAAATGAAT
          #22                         GACCGGAAGTGCACAAGAAATAGAATTATCACGCATGTATTCTTATCATTTCTTTTCTAAAAAATGAAT
          #24                         GACCGGAAGTGCACAAGAAATAGAATTATCACGCATGTATTCTTATCATTTCTTTTCTAAAAAATGAAT
          #26                         GACCGGAAGTGCACAAGAAATAGAATTATCACGCATGTATTCTTATCATTTCTTTTCTAAAAAATGAAT
          #29                         GACCGGAAGTGCACAAGAAATAGAATTATCACGCATGTATTCTTATCATTTCTTTTCTAAAAAATGAAT
          #48                         GACCGGAAGTGCACAAGAAATAGAATTATCACGCATGTATTCTTATCATTTCTTTTCTAAAAAATGAAT
          #34                         GACCGGAAGTGCACAAGAAATAGAATTATCACGCATGTATTCTTATCATTTCTTTTCTAAAAAATGAAT
          #13                         GACCGGAAGTGCACAAGAAATAGAATTATCACGCATGTATTCTTATCATTTCTTTTCTAAAAAATGAAT
          #6                              GGAAGTGCACAAGAAATAGAATTATCACGCATGTATTCTTATCATTTCTTTTCTAAAAAATGAAT
          #9                              GGAAGTGCACAAGAAATAGAATTATCACGCATGTATTCTTATCATTTCTTTTCTAAAAAATGAAT
          #21                             GGAAGTGCACAAGAAATAGAATTATCACGCATGTATTCTTATCATTTCTTTTCTAAAAAATGAAT
          #32                             GGAAGTGCACAAGAAATAGAATTATCACGCATGTATTCTTATCATTTCTTTTCTAAAAAATGAAT
          #4                                                                                    AAAAAAATGAAT
          #03                                                                                   AAAAAAATGAAT
```

pGKL2-ORF3

```
pGKL2

(5857)ATTATATGGATGTAGATATGATAAAATGTAATTCTGATTAGGAAAAGTATTG-50nts-TTTTAATCTGATTGTTATAATCAGATAGTAACATCTCACCCTTTTTAATACCATATTTAGAAATTATATATCTAAGGTAAACCTTTTGCATG(5664)

#26    GAGGAAAAAGTATTG-50nts-TTTTAATCTGATTGTTATAATCAGATAGTAACATCTCACCCTTTTTAATACCATATTTAGAAATTATATATCTAAGGTAAACCTTTTGCATG
#16    GAGGAAAAAGTATTG-50nts-TTTTAATCTGATTGTTATAATCAGATAGTAACATCTCACCCTTTTTAATACCATATTTAGAAATTATATATCTAAGGTAAACCTTTTGCATG
#4     GAGGAAAAAGTATTG-50nts-TTTTAATCTGATTGTTATAATCAGATAGTAACATCTCACCCTTTTTAATACCATATTTAGAAATTATATATCTAAGGTAAACCTTTTGCATG
#7     GAGGAAAAAGTATTG-50nts-TTTTAATCTGATTGTTATAATCAGATAGTAACATCTCACCCTTTTTAATACCATATTTAGAAATTATATATCTAAGGTAAACCTTTTGCATG
#25A    GGGAAAAAGTATTG-50nts-TTTTAATCTGATTGTTATAATCAGATAGTAACATCTCACCCTTTTTAATACCATATTTAGAAATTATATATCTAAGGTAAACCTTTTGCATG
#38     GAGGAAAAGTATTG-50nts-TTTTAATCTGATTGTTATAATCAGATAGTAACATCTCACCCTTTTTAATACCATATTTAGAAATTATATATCTAAGGTAAACCTTTTGCATG
#15     GAGGAAAAGTATTG-50nts-TTTTAATCTGATTGTTATAATCAGATAGTAACATCTCACCCTTTTTAATACCATATTTAGAAATTATATATCTAAGGTAAACCTTTTGCATG
#31A    GAGGAAAAGTATTG-50nts-TTTTAATCTGATTGTTATAATCAGATAGTAACATCTCACCCTTTTTAATACCATATTTAGAAATTATATATCTAAGGTAAACCTTTTGCATG
#35     GAGGAAAAGTATTG-50nts-TTTTAATCTGATTGTTATAATCAGATAGTAACATCTCACCCTTTTTAATACCATATTTAGAAATTATATATCTAAGGTAAACCTTTTGCATG
#44     GAGGAAAAGTATTG-50nts-TTTTAATCTGATTGTTATAATCAGATAGTAACATCTCACCCTTTTTAATACCATATTTAGAAATTATATATCTAAGGTAAACCTTTTGCATG
#27     GAGGAAAAGTATTG-50nts-TTTTAATCTGATTGTTATAATCAGATAGTAACATCTCACCCTTTTTAATACCATATTTAGAAATTATATATCTAAGGTAAACCTTTTGCATG
#10     GAAAAAAAGTATTG-50nts-TTTTAATCTGATTGTTATAATCAGATAGTAACATCTCACCCTTTTTAATACCATATTTAGAAATTATATATCTAAGGTAAACCTTTTGCATG
#39      AAAAAAAGTATTG-50nts-TTTTAATCTGATTGTTATAATCAGATAGTAACATCTCACCCTTTTTAATACCATATTTAGAAATTATATATCTAAGGTAAACCTTTTGCATG
#25      GGAAAAAGTATTG-50nts-TTTTAATCTGATTGTTATAATCAGATAGTAACATCTCACCCTTTTTAATACCATATTTAGAAATTATATATCTAAGGTAAACCTTTTGCATG
#32                                                     GAAAACATCTCACCCTTTTTAATACCATATTTAGAAATTATATATCTAAGGTAAACCTTTTGCATG
#39A                                                    AAAAACATCTCACCCTTTTTAATACCATATTTAGAAATTATATATCTAAGGTAAACCTTTTGCATG
#31                                                      GAAACATCTCACCCTTTTTAATACCATATTTAGAAATTATATATCTAAGGTAAACCTTTTGCATG
#37                                                      GAAACATCTCACCCTTTTTAATACCATATTTAGAAATTATATATCTAAGGTAAACCTTTTGCATG
#18                                                       GAACATCTCACCCTTTTTAATACCATATTTAGAAATTATATATCTAAGGTAAACCTTTTGCATG
#8                                                        GAACATCTCACCCTTTTTAATACCATATTTAGAAATTATATATCTAAGGTAAACCTTTTGCATG
#29                                                       GAACATCTCACCCTTTTTAATACCATATTTAGAAATTATATATCTAAGGTAAACCTTTTGCATG
#4a                                                       GAACATCTCACCCTTTTTAATACCATATTTAGAAATTATATATCTAAGGTAAACCTTTTGCATG
#34                                                       GAACATCTCACCCTTTTTAATACCATATTTAGAAATTATATATCTAAGGTAAACCTTTTGCATG
#3                                                        GAACATCTCACCCTTTTTAATACCATATTTAGAAATTATATATCTAAGGTAAACCTTTTGCATG

-50nts- = ATAAAACCGTATTGTTCTATACTTAAATTTTTATTAGTGAATACTTTCAT
```

pGKL2-ORF4

```
pGKL2 (5673)GGTTTACCTTAGATATATAATTTCTAAATATGGTATTAAAAAGGGTGAGATGTTACTATCTGATTATAACAATCAGATTAAAAATGAAA(5761)
                             #7                         GTGAGATGTTACTATCTGATTATAACAATCAGATTAAAAATGAAA
                             #34                        GTGAGATGTTACTATCTGATTATAACAATCAGATTAAAAATGAAA
                             #19                                      AAAAAAAAAAAAAAAAAAAAAAAAAATGAAA
                             #34A                                                    AAAAAAAAAATGAAA
                             #2                                                       AAAAAAAAATGAAA
                             #39                                                      GAAAAAAAATGAAA
                             #12                                                        AAAAAAATGAAA
                             #44a                                                       AAAAAAATGAAA
                             #28                                                        AAAAAAATGAAA
                             #41                                                        AAAAAAATGAAA
                             #35                                                          AAAAATGAAA
                             #29                                                          AAAAATGAAA
                             #24                                                          AAAAATGAAA
                             #19A                                                         GAAAATGAAA
                             #15                                                           GAAATGAAA
                             #M51                                                          GAAATGAAA
                             #25                                                           GAAATGAAA
                             #31                                                           GAAATGAAA
                             #13                                                           GAAATGAAA
                             #22                                                           GAAATGAAA
                             #21                                                           GAAATGAAA
                             #44                                                           GAAATGAAA
```

pGKL2-ORF5

```
pGKL2    (7361)GGAAAATTAATTTCTAATGTGAACACTAGTGAGATAGCTAAAGATCTTAATTGTGAAAACAATATTGAAAGTATAATAAATACATTAAAAGAACAAAATAGATATTTTGACAAACAAATTGAATATGATT(7490)

#46G     AAAAAAAAAAAGATCTTAATTGTGAAAACAATATTGAAAGTATAATAAATACATTAAAAGAACAAAATAGATATTTTGACAAACAAATTGAATATGATT
#45G      AAAAAAAAAAGATCTTAATTGTGAAAACAATATTGAAAGTATAATAAATACATTAAAAGAACAAAATAGATATTTTGACAAACAAATTGAATATGATT
#23D2     AAAAAAAAAAGATCTTAATTGTGAAAACAATATTGAAAGTATAATAAATACATTAAAAGAACAAAATAGATATTTTGACAAACAAATTGAATATGATT
A10       AAAAAAAAAAGATCTTAATTGTGAAAACAATATTGAAAGTATAATAAATACATTAAAAGAACAAAATAGATATTTTGACAAACAAATTGAATATGATT
#41G       AAAAAAAAAGATCTTAATTGTGAAAACAATATTGAAAGTATAATAAATACATTAAAAGAACAAAATAGATATTTTGACAAACAAATTGAATATGATT
#43G       AAAAAAAAAGATCTTAATTGTGAAAACAATATTGAAAGTATAATAAATACATTAAAAGAACAAAATAGATATTTTGACAAACAAATTGAATATGATT
A22b       AAAAAAAAAGATCTTAATTGTGAAAACAATATTGAAAGTATAATAAATACATTAAAAGAACAAAATAGATATTTTGACAAACAAATTGAATATGATT
#N6        GAAAAAAAAGATCTTAATTGTGAAAACAATATTGAAAGTATAATAAATACATTAAAAGAACAAAATAGATATTTTGACAAACAAATTGAATATGATT
#N10        AAAAAAAAGATCTTAATTGTGAAAACAATATTGAAAGTATAATAAATACATTAAAAGAACAAAATAGATATTTTGACAAACAAATTGAATATGATT
#35G         AAAAAAAGATCTTAATTGTGAAAACAATATTGAAAGTATAATAAATACATTAAAAGAACAAAATAGATATTTTGACAAACAAATTGAATATGATT
#36G         AAAAAAAGATCTTAATTGTGAAAACAATATTGAAAGTATAATAAATACATTAAAAGAACAAAATAGATATTTTGACAAACAAATTGAATATGATT
#37G         AAAAAAAGATCTTAATTGTGAAAACAATATTGAAAGTATAATAAATACATTAAAAGAACAAAATAGATATTTTGACAAACAAATTGAATATGATT
#N4          AAAAAAAGATCTTAATTGTGAAAACAATATTGAAAGTATAATAAATACATTAAAAGAACAAAATAGATATTTTGACAAACAAATTGAATATGATT
A6           AAAAAAAGATCTTAATTGTGAAAACAATATTGAAAGTATAATAAATACATTAAAAGAACAAAATAGATATTTTGACAAACAAATTGAATATGATT
A21          AAAAAAAGATCTTAATTGTGAAAACAATATTGAAAGTATAATAAATACATTAAAAGAACAAAATAGATATTTTGACAAACAAATTGAATATGATT
A24          AAAAAAAGATCTTAATTGTGAAAACAATATTGAAAGTATAATAAATACATTAAAAGAACAAAATAGATATTTTGACAAACAAATTGAATATGATT
A23b         AAAAAAAGATCTTAATTGTGAAAACAATATTGAAAGTATAATAAATACATTAAAAGAACAAAATAGATATTTTGACAAACAAATTGAATATGATT
#18A         GAAAAAAGATCTTAATTGTGAAAACAATATTGAAAGTATAATAAATACATTAAAAGAACAAAATAGATATTTTGACAAACAAATTGAATATGATT
A8           GAAAAAAGATCTTAATTGTGAAAACAATATTGAAAGTATAATAAATACATTAAAAGAACAAAATAGATATTTTGACAAACAAATTGAATATGATT
#20A          GAAAAAGATCTTAATTGTGAAAACAATATTGAAAGTATAATAAATACATTAAAAGAACAAAATAGATATTTTGACAAACAAATTGAATATGATT
#23A          GAAAAAGATCTTAATTGTGAAAACAATATTGAAAGTATAATAAATACATTAAAAGAACAAAATAGATATTTTGACAAACAAATTGAATATGATT
#26A          GAAAAAGATCTTAATTGTGAAAACAATATTGAAAGTATAATAAATACATTAAAAGAACAAAATAGATATTTTGACAAACAAATTGAATATGATT
#28A          GAAAAAGATCTTAATTGTGAAAACAATATTGAAAGTATAATAAATACATTAAAAGAACAAAATAGATATTTTGACAAACAAATTGAATATGATT
#22A          AAAAAAGATCTTAATTGTGAAAACAATATTGAAAGTATAATAAATACATTAAAAGAACAAAATAGATATTTTGACAAACAAATTGAATATGATT
A41           AAAAAAGATCTTAATTGTGAAAACAATATTGAAAGTATAATAAATACATTAAAAGAACAAAATAGATATTTTGACAAACAAATTGAATATGATT
A18            AAAAAGATCTTAATTGTGAAAACAATATTGAAAGTATAATAAATACATTAAAAGAACAAAATAGATATTTTGACAAACAAATTGAATATGATT
A45            AAAAAGATCTTAATTGTGAAAACAATATTGAAAGTATAATAAATACATTAAAAGAACAAAATAGATATTTTGACAAACAAATTGAATATGATT
A1             AAAAAGATCTTAATTGTGAAAACAATATTGAAAGTATAATAAATACATTAAAAGAACAAAATAGATATTTTGACAAACAAATTGAATATGATT
A44            AAAAAGATCTTAATTGTGAAAACAATATTGAAAGTATAATAAATACATTAAAAGAACAAAATAGATATTTTGACAAACAAATTGAATATGATT
A3             AAAAAGATCTTAATTGTGAAAACAATATTGAAAGTATAATAAATACATTAAAAGAACAAAATAGATATTTTGACAAACAAATTGAATATGATT
A11            AAAAAGATCTTAATTGTGAAAACAATATTGAAAGTATAATAAATACATTAAAAGAACAAAATAGATATTTTGACAAACAAATTGAATATGATT
A4             AAAAAGATCTTAATTGTGAAAACAATATTGAAAGTATAATAAATACATTAAAAGAACAAAATAGATATTTTGACAAACAAATTGAATATGATT
#21A           GAAAAGATCTTAATTGTGAAAACAATATTGAAAGTATAATAAATACATTAAAAGAACAAAATAGATATTTTGACAAACAAATTGAATATGATT
A2             GAAAAGATCTTAATTGTGAAAACAATATTGAAAGTATAATAAATACATTAAAAGAACAAAATAGATATTTTGACAAACAAATTGAATATGATT
#34G           GAAAAGATCTTAATTGTGAAAACAATATTGAAAGTATAATAAATACATTAAAAGAACAAAATAGATATTTTGACAAACAAATTGAATATGATT
#24A           GAAAAGATCTTAATTGTGAAAACAATATTGAAAGTATAATAAATACATTAAAAGAACAAAATAGATATTTTGACAAACAAATTGAATATGATT
A38            GAAAAGATCTTAATTGTGAAAACAATATTGAAAGTATAATAAATACATTAAAAGAACAAAATAGATATTTTGACAAACAAATTGAATATGATT
#38G           GAAAAGATCTTAATTGTGAAAACAATATTGAAAGTATAATAAATACATTAAAAGAACAAAATAGATATTTTGACAAACAAATTGAATATGATT
#N1            GAAAAGATCTTAATTGTGAAAACAATATTGAAAGTATAATAAATACATTAAAAGAACAAAATAGATATTTTGACAAACAAATTGAATATGATT
#40G           GAAAAGATCTTAATTGTGAAAACAATATTGAAAGTATAATAAATACATTAAAAGAACAAAATAGATATTTTGACAAACAAATTGAATATGATT
A9              AAAAGATCTTAATTGTGAAAACAATATTGAAAGTATAATAAATACATTAAAAGAACAAAATAGATATTTTGACAAACAAATTGAATATGATT
A4E1            AAAAGATCTTAATTGTGAAAACAATATTGAAAGTATAATAAATACATTAAAAGAACAAAATAGATATTTTGACAAACAAATTGAATATGATT
#N5             AAAAGATCTTAATTGTGAAAACAATATTGAAAGTATAATAAATACATTAAAAGAACAAAATAGATATTTTGACAAACAAATTGAATATGATT
A22a            AAAAGATCTTAATTGTGAAAACAATATTGAAAGTATAATAAATACATTAAAAGAACAAAATAGATATTTTGACAAACAAATTGAATATGATT
A20             AAAAGATCTTAATTGTGAAAACAATATTGAAAGTATAATAAATACATTAAAAGAACAAAATAGATATTTTGACAAACAAATTGAATATGATT
#25A             AAAGATCTTAATTGTGAAAACAATATTGAAAGTATAATAAATACATTAAAAGAACAAAATAGATATTTTGACAAACAAATTGAATATGATT
A46              AAAGATCTTAATTGTGAAAACAATATTGAAAGTATAATAAATACATTAAAAGAACAAAATAGATATTTTGACAAACAAATTGAATATGATT
A12              AAAGATCTTAATTGTGAAAACAATATTGAAAGTATAATAAATACATTAAAAGAACAAAATAGATATTTTGACAAACAAATTGAATATGATT
#44G             GAAGATCTTAATTGTGAAAACAATATTGAAAGTATAATAAATACATTAAAAGAACAAAATAGATATTTTGACAAACAAATTGAATATGATT
A7               GAAGATCTTAATTGTGAAAACAATATTGAAAGTATAATAAATACATTAAAAGAACAAAATAGATATTTTGACAAACAAATTGAATATGATT
```

pGKL2-ORF6

```
pGKL2   (7932)GAATTTGACAAACTATCATTAGCTATATGATCGTATTAGATATACCTAAAATGGAT(7987)

#46     AAAAAAAAAAAAAATGGAT
#27       TGAAAAAAAAAATGGAT
#48        GAAAAAAAAAATGGAT
#34         AAAAAAAAAATGGAT
#35         AAAAAAAAAATGGAT
#45         AAAAAAAAAATGGAT
#44         AAAAAAAAAATGGAT
#13         AAAAAAAAAATGGAT
#28a        AAAAAAAAAATGGAT
#33         AAAAAAAAAATGGAT
#37         AAAAAAAAAATGGAT
#6           AAAAAAAAATGGAT
#36          AAAAAAAAATGGAT
#48A         AAAAAAAAATGGAT
#43          AAAAAAAAATGGAT
#8           AAAAAAAAATGGAT
#12          AAAAAAAAATGGAT
#22          AAAAAAAAATGGAT
#2            AAAAAAAATGGAT
#31A          AAAAAAAATGGAT
#38A          AAAAAAAATGGAT
#39           AAAAAAAATGGAT
#26A           GAAAAAATGGAT
#32A            GAAAAATGGAT
#38             GAAAAATGGAT
```

pGKL2-ORF7

```
pGKL2   (11294)AAAGAATGCTGAATTTACAATTTTATGTGAAGTTGATGATATAAAGTAAAAATGAAT(11350)

#40     AAAAAAAAAAAAAAAAAAAAAAAATGAAT
#41         AAAAAAAAAAAAAAAAAAAATGAAT
#4T7         AAAAAAAAAAAAAAAAAAATGAAT
#18            AAAAAAAAAAAAAAAAATGAAT
#6              AAAAAAAAAAAAAAAATGAAT
#7              AAAAAAAAAAAAAAAATGAAT
#39b             AAAAAAAAAAAAAAATGAAT
#33b              AAAAAAAAAAAAAATGAAT
#32b               AAAAAAAAAAAAATGAAT
#45                AAAAAAAAAAAAATGAAT
#20                 AAAAAAAAAAAATGAAT
#35                 AAAAAAAAAAAATGAAT
#33c                AAAAAAAAAAAATGAAT
#39                  AAAAAAAAAAATGAAT
#23                  AAAAAAAAAAATGAAT
#17                  AAAAAAAAAAATGAAT
#16                   AAAAAAAAAATGAAT
#32                   AAAAAAAAAATGAAT
#36                     AAAAAAAATGAAT
#10                     AAAAAAAATGAAT
#15                      AAAAAAATGAAT
#25                       GAAAAATGAAT
#47                        AAAAATGAAT
```

pGKL2-ORF8

```
pGKL2   (11597)ATTCGTCTATGCTTTTATATTTGAATTCTACTTTATGCTCAATAATCTCCATAATGTAT(11539)

#46       AAAAAAAAATCTCCATAATGTAT
#9D        AAAAAAAATCTCCATAATGTAT
#9EC       GAAAATAATCTCCATAATGTAT
#1D        GAAAATAATCTCCATAATGTAT
#7D        GAAAATAATCTCCATAATGTAT
#5          GAAATAATCTCCATAATGTAT
#28R2       GAAATAATCTCCATAATGTAT
#4D         GAAATAATCTCCATAATGTAT
#11D        GAAATAATCTCCATAATGTAT
#42         AAATAAATCTCCATAATGTAT
#6           GAATAATCTCCATAATGTAT
#6D          AAAAAATCTCCATAATGTAT
#48          GAAAAATCTCCATAATGTAT
#1            GAAAATCTCCATAATGTAT
#20D          GAAAATCTCCATAATGTAT
#3            GAAAATCTCCATAATGTAT
#33BT         GAAAATCTCCATAATGTAT
#38BT         GAAAATCTCCATAATGTAT
#V18AT        GGGAATCTCCATAATGTAT
#3             AAAATCTCCATAATGTAT
#23D           AAAATCTCCATAATGTAT
#39BT          AAAATCTCCATAATGTAT
#7             GAAATCTCCATAATGTAT
#19D           GAAATCTCCATAATGTAT
#14            GAAATCTCCATAATGTAT
#31A           GAAATCTCCATAATGTAT
#47R2          GAAATCTCCATAATGTAT
#8EC           GAAATCTCCATAATGTAT
#28D           GAAATCTCCATAATGTAT
#25D           GAAATCTCCATAATGTAT
#22AT          GAAATCTCCATAATGTAT
#1EC            AAATCTCCATAATGTAT
#20AT           AAATCTCCATAATGTAT
#27D            AAATCTCCATAATGTAT
#3D             AAATCTCCATAATGTAT
#31            GATCTCTCCATAATGTAT
#2              GAATCTCCATAATGTAT
#22D            GAATCTCCATAATGTAT
#23AT           GAATCTCCATAATGTAT
#32BT           GAATCTCCATAATGTAT
#21AT           GAATCTCCATAATGTAT
#26D            GAATCTCCATAATGTAT
#7AT            GAATCTCCATAATGTAT
#18FC           GAATCTCCATAATGTAT
#45FC           GAATCTCCATAATGTAT
#44FC           GAATCTCCATAATGTAT
#43FC           GAATCTCCATAATGTAT
#29BT           GAATCTCCATAATGTAT
#12EC           GAATCTCCATAATGTAT
#10EC           GAATCTCCATAATGTAT
#43BT           GAATCTCCATAATGTAT
#2AT            GAATCTCCATAATGTAT
#7EC            GAATCTCCATAATGTAT
#6EC            GAATCTCCATAATGTAT
#4EC            GAATCTCCATAATGTAT
#3EC            GAATCTCCATAATGTAT
#2EC            GAATCTCCATAATGTAT
#2D             GAATCTCCATAATGTAT
#24D            GAATCTCCATAATGTAT
#9A             GAATCTCCATAATGTAT
#13D            GAATCTCCATAATGTAT
#43             GAATCTCCATAATGTAT
#37             GAATCTCCATAATGTAT
#35             GAATCTCCATAATGTAT
#22             GAATCTCCATAATGTAT
#19             GAATCTCCATAATGTAT
#6A             GAATCTCCATAATGTAT
#10D            GAATCTCCATAATGTAT
#20             GAATCTCCATAATGTAT
#8D             GAATCTCCATAATGTAT
#21D            GAATCTCCATAATGTAT
#14D            GAATCTCCATAATGTAT
#17D            GAATCTCCATAATGTAT
#8              GAATCTCCATAATGTAT
#13             GAATCTCCATAATGTAT
#12             GAATCTCCATAATGTAT
#10             GAATCTCCATAATGTAT
#9              GAATCTCCATAATGTAT
#24AT           GAATCTCCATAATGTAT
```

pGKL2-ORF9

```
pGKL2 (11490)TGGAAACATTATTTGAATAGTATTCGACAAATGAAACATTGGTTATCTTATACATTATGGAG(11551)

       #45                     AAAAAAAAACATTGGTTATCTTATACATTATGGAG
       #10                     AAAAAAAAACATTGGTTATCTTATACATTATGGAG
       #36                     AAAAAAAAACATTGGTTATCTTATACATTATGGAG
       #9                       AAAAAAAACATTGGTTATCTTATACATTATGGAG
       #32                      AAAAAAAACATTGGTTATCTTATACATTATGGAG
       #47                      AAAAAAAACATTGGTTATCTTATACATTATGGAG
       #4                        AAAAAAACATTGGTTATCTTATACATTATGGAG
       #22                       AAAAAAACATTGGTTATCTTATACATTATGGAG
       #8A                       AAAAAAACATTGGTTATCTTATACATTATGGAG
       #31A                      AAAAAAACATTGGTTATCTTATACATTATGGAG
       #19A                      AAAAAAACATTGGTTATCTTATACATTATGGAG
       #41                        AAAAAACATTGGTTATCTTATACATTATGGAG
       #19                        GAAAAACATTGGTTATCTTATACATTATGGAG
       #44                        GAAAAACATTGGTTATCTTATACATTATGGAG
       #18                         AAAAACATTGGTTATCTTATACATTATGGAG
       #31                         AAAAACATTGGTTATCTTATACATTATGGAG
       #17                         AAAAACATTGGTTATCTTATACATTATGGAG
       #40                         AAAAACATTGGTTATCTTATACATTATGGAG
       #26                         GAAAACATTGGTTATCTTATACATTATGGAG
       #47A                        GAAAACATTGGTTATCTTATACATTATGGAG
       #16                          GAAACATTGGTTATCTTATACATTATGGAG
       #29                          GAAACATTGGTTATCTTATACATTATGGAG
       #30                          GAAACATTGGTTATCTTATACATTATGGAG
       #43                          GAAACATTGGTTATCTTATACATTATGGAG
```

pGKL2-ORF10

```
pGKL2          (12861)AATATCAGAAAAATGTAGAAATATATGATAAGCTCATAGACATGTAAAATGGCT(12914)

                 #14                      AAAAAAAAAAAAAAAAAAAATGGCT
                 #5                               AAAAAAAAAAAAATGGCT
                 #3                                 AAAAAAAAAAATGGCT
                 #8                                  AAAAAAAAAATGGCT
                 #12                                 AAAAAAAAAATGGCT
                 #18                                 AAAAAAAAAATGGCT
                 #19                                 AAAAAAAAAATGGCT
                 #16                                 AAAAAAAAAATGGCT
                 #11                                  AAAAAAAAATGGCT
                 #13                                  AAAAAAAAATGGCT
                 #7                                    AAAAAAAATGGCT
                 #24                                   AAAAAAAATGGCT
                 #4                                     AAAAAAATGGCT
                 #23                                    AAAAAAATGGCT
                 #6                                     AAAAAAATGGCT
                 #15                                    AAAAAAATGGCT
                 #17                                    AAAAAAATGGCT
                 #22                                      AAAAATGGCT
                 #1                                        AAAATGGCT
                 #9                                        AAAATGGCT
```

pGKL2-ORF11

```
pGKL2 (5505)TACATCTACCATAGCAGATTCCAGATTTGATTTATATTTAGCGAATCTTTTAAACATGCCT(5565)

                 #5                         AAAAAAAAAAAAACATGCCT
                 #29                         AAAAAAAAAAAACATGCCT
                 #8                           AAAAAAAAAAACATGCCT
                 #2                           AAAAAAAAAAACATGCCT
                 #38                          AAAAAAAAAAACATGCCT
                 #43                           AAAAAAAAAACATGCCT
                 #22                           AAAAAAAAAACATGCCT
                 #21                           AAAAAAAAAACATGCCT
                 #6                            AAAAAAAAAACATGCCT
                 #1a                           AAAAAAAAAACATGCCT
                 #1                             AAAAAAAAACATGCCT
                 #30                            AAAAAAAAACATGCCT
                 #14                             AAAAAAAACATGCCT
                 #7a                             AAAAAAAACATGCCT
                 #7                              AAAAAAAACATGCCT
                 #32                             AAAAAAAACATGCCT
                 #10                              AAAAAAACATGCCT
                 #2a                              AAAAAAACATGCCT
                 #12                               GAAAAACATGCCT
                 #3                                 AAAAACATGCCT
```

B - The 5' untranslated regions of actin encoded by *K. lactis* IFO1267 strain

Kluyveromyces lactis **actin**

```
Kluyveromyces lactis strain NRRL Y-1140 chromosome D

(461643)AGAAAGGTTGTTTCAGAATAACGGTTTTTTTGTTGATTGCAAAAGGAATGTACAACATCTTCACGCGCTATAGTATAACAATATGGAT(461730)

                        #12s          GGTACAACATCTTCACGCGCTATAGTATAACAATATGGAT
                        #16s          GGTACAACATCTTCACGCGCTATAGTATAACAATATGGAT
                        #28C          GGTACAACATCTTCACGCGCTATAGTATAACAATATGGAT
                        #27C          GGTACAACATCTTCACGCGCTATAGTATAACAATATGGAT
                        #26C          GGTACAACATCTTCACGCGCTATAGTATAACAATATGGAT
                        #29C          GGTACAACATCTTCACGCGCTATAGTATAACAATATGGAT
                        #14G          GGTACAACATCTTCACGCGCTATAGTATAACAATATGGAT
                        #16G          GGTACAACATCTTCACGCGCTATAGTATAACAATATGGAT
                        #30C          GGTACAACATCTTCACGCGCTATAGTATAACAATATGGAT
                        #5A           GGTACAACATCTTCACGCGCTATAGTATAACAATATGGAT
                        #6A           GGTACAACATCTTCACGCGCTATAGTATAACAATATGGAT
                        #7A           GGTACAACATCTTCACGCGCTATAGTATAACAATATGGAT
                        #8A           GGTACAACATCTTCACGCGCTATAGTATAACAATATGGAT
                        #2A           GGTACAACATCTTCACGCGCTATAGTATAACAATATGGAT
                        #20G          GGTACAACATCTTCACGCGCTATAGTATAACAATATGGAT
                        #23G          GGTACAACATCTTCACGCGCTATAGTATAACAATATGGAT
                        #24G          GGTACAACATCTTCACGCGCTATAGTATAACAATATGGAT
                        #25G          GGTACAACATCTTCACGCGCTATAGTATAACAATATGGAT
                        #11A          GGTACAACATCTTCACGCGCTATAGTATAACAATATGGAT
                        #12A          GGTACAACATCTTCACGCGCTATAGTATAACAATATGGAT
                        #4C3          GGTACAACATCTTCACGCGCTATAGTATAACAATATGGAT
                        #5C3          GGTACAACATCTTCACGCGCTATAGTATAACAATATGGAT
                        #6C3          GGTACAACATCTTCACGCGCTATAGTATAACAATATGGAT
                        #7C3          GGTACAACATCTTCACGCGCTATAGTATAACAATATGGAT
                        #9C3          GGTACAACATCTTCACGCGCTATAGTATAACAATATGGAT
                        #10C3         GGTACAACATCTTCACGCGCTATAGTATAACAATATGGAT
                        #32D10        GGTACAACATCTTCACGCGCTATAGTATAACAATATGGAT
                        #1A           GGTACAACATCTTCACGCGCTATAGTATAACAATATGGAT
                        #39D10        GGTACAACATCTTCACGCGCTATAGTATAACAATATGGAT
                        #36D10        GGTACAACATCTTCACGCGCTATAGTATAACAATATGGAT
                        #37D10        GGTACAACATCTTCACGCGCTATAGTATAACAATATGGAT
                        #2C3          GGTACAACATCTTCACGCGCTATAGTATAACAATATGGAT
                        #3C3          GGTACAACATCTTCACGCGCTATAGTATAACAATATGGAT
                        #47s          GGTACAACATCTTCACGCGCTATAGTATAACAATATGGAT
                        #42s          GGTACAACATCTTCACGCGCTATAGTATAACAATATGGAT
                        #18s          GGTACAACATCTTCACGCGCTATAGTATAACAATATGGAT
                        #4C           GGTACAACATCTTCACGCGCTATAGTATAACAATATGGAT
                        #5C           GGTACAACATCTTCACGCGCTATAGTATAACAATATGGAT
                        #7C           GGTACAACATCTTCACGCGCTATAGTATAACAATATGGAT
                        #8C           GGTACAACATCTTCACGCGCTATAGTATAACAATATGGAT
                        #11C          GGTACAACATCTTCACGCGCTATAGTATAACAATATGGAT
                        #17C          GGTACAACATCTTCACGCGCTATAGTATAACAATATGGAT
                        #9A                  GATCTTCACGCGCTATAGTATAACAATATGGAT
                        #33D10                     GACGCGCTATAGTATAACAATATGGAT
                        #1C                        GACGCGCTATAGTATAACAATATGGAT
                        #3C                        GACGCGCTATAGTATAACAATATGGAT
                        #2C                                       GAACAATATGGAT
```

C - The 3' untranslated regions of selected ORFs encoded by pGKL plasmids

pGKL1-ORF1 (3' RACE)

```
pGKL1        (3198)TAATATATGAAAGTTTTTATAATAATTATAAAATGAATATATTTTACATATTTTTGTTTTTGCTGTCATTCGTTCAAGGTTTGGAGCATACTCATCGAAGAGGCTCCTTAGTC(3310)

          #2       TAATATATGAAAGTTTTTATAATAATTATAAAATGAATATATTTTACATATTTTTGTTTTTGCTGTCATTCGTTCAAGGTTTGGAGCATACTCATCGAAGAGGCTCCTTAG
          #38      TAATATATGAAAGTTTTTATAATAATTATAAAATGAATATATTTTACATATTTTTGTTTTTGCT
          #4       TAATATATGAAAGTTTTTATAATAATTATAAAATGAATATATTTTACATATTTTTGTTTTTGCT
          #8       TAATATATGAAAGTTTTTATAATAATTATAAAATGAATATATTTTACATATTTTTGTTTTTGCT
          #5       TAATATATGAAAGTTTTTATAATAATTATAAAATGAATATATTTTACATATTTTTGTTTTTGC
          #6       TAATATATGAAAGTTTTTATAATAATTATAAAATGAATATATTTTACATATTTTTGTTTTTGC
          #3A      TAATATATGAAAGTTTTTATAATAATTATAAAATGAATATATTTTACATATTTTTGTTTTTGC
          #45      TAATATATGAAAGTTTTTATAATAATTATAAAATGAATATATTTTACATATTTTTGTTTTTGC
          #5A      TAATATATGAAAGTTTTTATAATAATTATAAAATGAATATATTTTACATATTTTTGTTTTTG
          #42      TAATATATGAAAGTTTTTATAATAATTATAAAATGAATATATTTTACATATTTTTGTTTTT
          #43      TAATATATGAAAGTTTTTATAATAATTATAAAATGAATATATTTTACATATTTTTGTTTTT
          #44      TAATATATGAAAGTTTTTATAATAATTATAAAATGAATATATTTTACATATTTTTGTTTTT
          #39      TAATATATGAAAGTTTTTATAATAATTATAAAATGAATATATTTTACATATTTTTGTTTT
          #7       TAATATATGAAAGTTTTTATAATAATTATAAAATGAATATATTTTACATATTTTTGTTT
          #3       TAATATATGAAAGTTTTTATAATAATTATAAAATGAATATATTTTACATATTTTT
          #46      TAATATATGAAAGTTTTTATAATAATTATAAAATGAATATATTTTACATATTTTT
          #31      TAATATATGAAAGTTTTTATAATAATTATAAAATGAATATATTTTACATATTTT
          #47      TAATATATGAAAGTTTTTATAATAATTATAAAATGAATATATTTTACATATTTT
          #4A      TAATATATGAAAGTTTTTATAATAATTATAAAATGAATATATTTTACATATTTT
          #37      TAATATATGAAAGTTTTTATAATAATTATAAAATGAATATATTTTACATATTT
          #2A      TAATATATGAAAGTTTT
```

pGKL2-ORF5 (3' RACE)

```
pGKL2   (7959)TGA-114nt-AACTAAGTTACTGTGCTATATACAATGACAGAGAACTTCCTATAATGATTGGTAGTTCACTGGATATTAGCAAAGAAAAT(8155)

    #9        TGA-114nts-AACTAAGTTACTGTGCTATATACAATGACAGAGAACTTCCTATAATGATTGGTAGTTCACTGGATATTAGCAAA
    #14       TGA-114nts-AACTAAGTTACTGTGCTATATACAATGACAGAGAACTTCCTATAATGATTGGTAGTTCACTGGATATTAGCAAA
    #18       TGA-114nts-AACTAAGTTACTGTGCTATATACAATGACAGAGAACTTCCTATAATGATTGGTAGTTCACTGGATATTAGCAAA
    #N15      TGA-114nts-AACTAAGTTACTGTGCTATATACAATGACAGAGAACTTCCTATAATGATTGGTAGTTCACTGGATATTAGCAAA
    #4b       TGA-114nts-AACTAAGTTACTGTGCTATATACAATGACAGAGAACTTCCTATAATGATTGGTAGTTCACTGGATATTAGCAA
    #13       TGA-114nts-AACTAAGTTACTGTGCTATATACAATGACAGAGAACTTCCTATAATGATTGGTAGTTCACTGGATATTAGCAA
    #24       TGA-114nts-AACTAAGTTACTGTGCTATATACAATGACAGAGAACTTCCTATAATGATTGGTAGTTCACTGGATATTAGCAA
    #23       TGA-114nts-AACTAAGTTACTGTGCTATATACAATGACAGAGAACTTCCTATAATGATTGGTAGTTCACTGGATATTAGCAA
    #8        TGA-114nts-AACTAAGTTACTGTGCTATATACAATGACAGAGAACTTCCTATAATGATTGGTAGTTCACTGGATATTAGCAA
    #11       TGA-114nts-AACTAAGTTACTGTGCTATATACAATGACAGAGAACTTCCTATAATGATTGGTAGTTCACTGGATATTAGCAA
    #20       TGA-114nts-AACTAAGTTACTGTGCTATATACAATGACAGAGAACTTCCTATAATGATTGGTAGTTCACTGGATATTAGCAA
    #19       TGA-114nts-AACTAAGTTACTGTGCTATATACAATGACAGAGAACTTCCTATAATGATTGGTAGTTCACTGGATATTAGCAA
    #15       TGA-114nts-AACTAAGTTACTGTGCTATATACAATGACAGAGAACTTCCTATAATGATTGGTAGTTCACTGGATATTAGCAA
    #21       TGA-114nts-AACTAAGTTACTGTGCTATATACAATGACAGAGAACTTCCTATAATGATTGGTAGTTCACTGGATATTAGCA
    #12       TGA-114nts-AACTAAGTTACTGTGCTATATACAATGACAGAGAACTTCCTATAATGATTGGTAGTTCACTGGATATTAG
    #17       TGA-114nts-AACTAAGTTACTGTGCTATATACAATGACAGAGAACTTCCTATAATGATTGGTAGTTCACTGGATATTAG
    #6        TGA-114nts-AACTAAGTTACTGTGCTATATACAATGACAGAGAACTTCCTATAATGATTGGTA
    #5b       TGA-114nts-AACTAAGTTACTGTGCTATATACAATGACAGAGAACTTCCTATAATGA
    #21       TGA-114nts-AACTAAGTTACTGTGCTATATACAATGACAGAGAACTTCCTATAATG
    #4a       TGA-114nts-AACTAAGTTACTGTGCTATATACAATGACAGAGAACTTCCTATAAT
    #16       TGA-114nts-AACTAAGTTACTGTGCTATATACAATGACAGAGAACTTCCTATAAT
    #5a       TGA-114nts-AACTAAGTTACTGTGCTATATACAATGACAGAGAACTTCCTATAAT
    #13       TGA-114nts-AACTAAGTTACTGTGCTATATACAATGACAGAGAACTTCCTATAAT
    #3        TGA-114nts-AACTAAGTTACTGTGCTATATACAATGACAGAGAACTTCCTATAAT
    #22       TGA-114nts-AACTAAGTTACTGTGCTATATACAATGACAGAGAACTTCCTATAAT
    #N14      TGA-114nts-AACTAAGTTACTGTGCTATATACAATGACAGAGAACTTCCTATAA
    #17       TGA-114nts-AACTAAGTTACTGTGCTATATACA
```

```
-114nts-
    =
TCGTATTAGATATACCTAAAATGGATTATGGACAAATAGAAATTTATAACGATTATTTTAGAAATGTTGATAAGAAAACTATAAAATACACTCCCATGGAGTGCATAGAATATA
```

pGKL2-ORF10 (3' RACE)

```
 pGKL2        (13218)TAAATTATTATGTAACATTAAATATACATTTAACACTGTATGCTACTATAACTGATTTTT(13277)

         #3          TAAATTATTATGTAACATTAAATATACATTTAACACTGTATGCTACTATAACTGAT
         #27         TAAATTATTATGTAACATTAAATATACATTTAACACTGTATGCTACTATAACTGAT
         #5          TAAATTATTATGTAACATTAAATATACATTTAACACTGTATGCTACTATAACTGAT
         #25         TAAATTATTATGTAACATTAAATATACATTTAACACTGTATGCTACTATAACTGAT
         #23         TAAATTATTATGTAACATTAAATATACATTTAACACTGTATGCTACTATAACTGAT
         #22         TAAATTATTATGTAACATTAAATATACATTTAACACTGTATGCTACTATAACTGAT
         #21         TAAATTATTATGTAACATTAAATATACATTTAACACTGTATGCTACTATAACTGAT
         #20         TAAATTATTATGTAACATTAAATATACATTTAACACTGTATGCTACTATAACTGAT
         #19         TAAATTATTATGTAACATTAAATATACATTTAACACTGTATGCTACTATAACTGAT
         #6          TAAATTATTATGTAACATTAAATATACATTTAACACTGTATGCTACTATAACTGAT
         #16         TAAATTATTATGTAACATTAAATATACATTTAACACTGTATGCTACTATAACTGAT
         #8          TAAATTATTATGTAACATTAAATATACATTTAACACTGTATGCTACTATAACTGAT
         #10         TAAATTATTATGTAACATTAAATATACATTTAACACTGTATGCTACTATAACTGAT
         #13         TAAATTATTATGTAACATTAAATATACATTTAACACTGTATGCTACTATAACTGAT
         #1          TAAATTATTATGTAACATTAAATATACATTTAACACTGTATGCTACTATAACTGA
         #9          TAAATTATTATGTAACATTAAATATACATTTAACACTGTATGCTACTATAACTGA
         #14         TAAATTATTATGTAACATTAAATATACATTTAACACTGTATGCTACTATAACTGA
         #15         TAAATTATTATGTAACATTAAATATACATTTAACACTGTATGCTACTATAACTGA
         #2          TAAATTATTATGTAACATTAAATATACATTTAACACTGTATGCTACTATAACTGA
         #4          TAAATTATTATGTAACATTAAATATACATTTAACACTGTATGCTACTATAACTGA
         #17         TAAATTATTATGTAACATTAAATATACATTTAACACTGTATGCTACTATAACTG
         #26         TAAATTATTATGTAACATTAAATATACATTTAACACTGTATGCTACTATAACTG
```

(A) The 5' untranslated regions of ORFs encoded by pGKL plasmids. Plasmid DNA (pGKL1 or pGKL2 respectively) is shown for each ORF with the AUG initiation site (marked by vertical line and arrow which points in the direction of transcription) and part of DNA upstream of the start codon including Upstream Conserved Sequence (UCS, in yellow box). In the case of *K1ORF4*, *K2ORF3* and *K2ORF4*, new putative UCS sequences were found (shown in blue box), based on the detected transcripts. Numbers on both sides of the plasmid sequence indicate the position of the DNA sequence relative to the GI: 163932456 (for pGKL1) or GI: 2868 (for pGKL2), respectively. Individual obtained cDNA sequences corresponding to transcripts of a given ORF are aligned below the template DNA. After isolation of mRNA from *K. lactis* IFO1267 strain, cDNA was prepared by SuperScript III Reverse Transcriptase (M-MuLV mutant; clone names beginning with the number sign #), or by AMV Reverse Transcriptase (Finnzymes; clone names beginning with the letter A; this was performed only for *K1ORF2*, *K1ORF3* and *K2ORF5*). In this comparison, we have also included clones obtained from a double-mutant strain *K. lactisΔpbp1Δpab1* (see text; clone names beginning with the letter P; this was performed only for *K1ORF2* and *K1ORF3*). From the uniform distribution of the clones is evident that neither used reverse transcriptase nor strain used for mRNA isolation / cDNA synthesis / clone preparation affects the results. At least 20 independent clones were sequenced and aligned with plasmid (template) DNA sequence of each ORF. Sequence parts that show sequence identity to the template plasmid DNA are labelled in red. Sequence parts that do not show sequence identity to the template plasmid DNA, thus contain non-templated nucleotides of mRNA transcripts are labelled in green. Guanosine residues which correspond to the original caps at the 5' ends of the mRNAs are depicted as underlined black G. All sequences are shown in 5' to 3' transcription orientation, regardless of ORF natural orientation in pGKL plasmids.

(B) The 5' untranslated regions of actin gene (KLLA0_D05357g) encoded by *K. lactis* IFO1267 strain. Chromosomal DNA is shown for *ACT* gene AUG initiation site including (marked by vertical line and arrow which points in the direction of transcription) and part of DNA upstream of the start codon. Numbers on both sides of the chromosomal sequence indicate the position of the DNA sequence relative to the EnsemblFungi Id: KLLA0_D05357g. Individual obtained cDNA sequences corresponding to transcripts of a given ORF are aligned below the template DNA.

(C) The 3' untranslated regions of *K1ORF1*, *K2ORF5* and *K2ORF10* encoded by pGKL plasmids. Template DNA with indicated stop codon (in the gray box) is shown for each ORF. Numbers on both sides of the plasmid sequence indicate the position of the DNA sequence relative to the GenBank records indicated above. Unique obtained sequences (prepared using SuperScript III Reverse Transcriptase) corresponding to transcripts of a given ORF are aligned under the template DNA. At least 20 independent clones were sequenced and aligned with plasmid (template) DNA of each ORF.

Table S6. 5' mRNA cap occurrence frequency in pGKL transcripts with different number of non-templated adenosines in 5' poly(A) leader.

No. of non-templated adenosines	No. of sequences	No. of sequences with 5' mRNA cap	5' mRNA cap occurrence frequency
0	88	72	82%
1	25	19	76%
2	37	20	54%
3	23	6	26%
4	30	2	7%
5	33	1	3%
6+	114	3	2.6%

Table S7. The degree of 5' capping and 5' polyadenylation of pGKL mRNAs is determined by the respective UCR promoter region.

	**% of 5' non-templately polyadenylated RNAs**	**% of 5' capped RNAs**	**Median / mean of adenosines added per mRNA**	**No. of 5' non-templated adenosines**
***K2ORF2* under control of native promoter ***	8.2	91.7	0.0 / 0.8	1
***K2ORF2* under control of *K1UCR1* promoter**	92.3	7.7	5.0 / 4.9	2-11
***K1ORF1* under control of native promoter ***	85.1	22.2	5.0 / 5.3	1-10
**G418 under control of *K1UCR2 promoter***	96.4	3.6	5.5 / 5.3	0-10
***K1ORF2* under control of native promoter ***	96.2	7.5	6.0 / 6.0	2-14

Data represents 5' RACE analysis of the *K1ORF1*, *K1ORF2*, *K2ORF2* and *G418*^{R} mRNAs in the *K. lactis* IFO1267_pRKL2-1 strain and/or its parental strain *K. lactis* IFO1267 (marked with asterisk).

Table S8. Number of non-templated adenosine nucleotides in pGKL transcripts increases with the number of 5' poly(A) leader adenosines encoded by the template.

Templated adenosines	No. of sequences	Non-templated adenosines (median / mean)
2	36	2.0 / 2.5
3	139	4.0 / 4.1
4	94	6.0 / 5.8
5	104	6.0 / 6.8

Table S9. 5' RACE analysis revealed that deletion of the *PAB1* gene does not remarkably affect the structure of the 5' ends of the pGKL mRNAs in *K. lactis* cells.

	% of 5' non-templately polyadenylated mRNAs	% of 5' capped mRNAs	Median / mean of adenosines added per mRNA	No. of 5' non-templated adenosines
K1ORF2 **(IFO1267) ***	96.2	7.5	6.0 / 6.0	2-14
K1ORF2 **IFO1267 *pbp1Δ pab1Δ***	100.0	0.0	7.0 / 7.2	5-10
K1ORF3 **(IFO1267) ***	98.2	5.3	7.0 / 6.7	0-20
K1ORF3 **IFO1267 *pbp1Δ pab1Δ***	100.0	14.2	4.0 / 5.5	1-11

Data represents 5' RACE analysis of the *K1ORF2* and *K1ORF3* mRNAs in the *K. lactis* IFO1267 *pbp1Δ pab1Δ* strain and its parental strain *K. lactis* IFO1267 (marked with asterisk).

Table S10. Detailed statistical results of the length of 5' poly(A) leaders as depicted in Figure 4. Adjusted p-values corresponding to all possible ORF pairs are depicted in the triangle below**.**

	K1ORF1	*K1ORF2*	*K1ORF3*	*K1ORF4*	*K2ORF1*	*K2ORF10*	*K2ORF11*	*K2ORF4*	*K2ORF5*	*K2ORF6*	*K2ORF7*	*K2ORF8*
K1ORF2	7.7550E-02											
K1ORF3	2.3586E-03	1.5796E-01										
K1ORF4	9.4012E-01	1.3077E-01	6.8512E-03									
K2ORF1	7.1584E-07	4.7946E-15	5.1673E-20	1.8159E-06								
K2ORF10	7.9875E-01	5.3549E-02	2.0836E-03	7.5276E-01	2.5665E-05							
K2ORF11	6.3346E-01	3.9942E-01	4.4490E-02	7.1093E-01	2.6219E-07	4.6810E-01						
K2ORF4	1.8324E-05	1.5043E-10	6.2869E-14	2.7037E-05	9.5564E-01	1.9880E-04	5.2996E-06					
K2ORF5	3.7964E-07	2.1512E-16	7.3018E-22	1.1537E-06	9.2680E-01	1.9815E-05	1.6321E-07	9.0119E-01				
K2ORF6	6.3971E-01	3.1921E-01	2.3704E-02	7.1093E-01	4.8730E-08	4.6810E-01	9.5564E-01	2.0464E-06	2.2551E-08			
K2ORF7	1.4373E-03	6.2947E-02	5.0437E-01	3.3298E-03	7.6681E-16	1.1487E-03	1.8723E-02	2.0175E-12	1.2879E-16	1.0618E-02		
K2ORF8	9.9868E-23	3.8536E-44	1.0758E-52	1.9026E-20	2.1119E-07	1.6511E-17	2.6452E-21	5.7407E-05	8.2255E-09	2.8832E-24	*4.0538E-36*	
K2ORF9	5.5264E-05	3.1585E-10	8.0237E-14	8.2700E-05	7.1093E-01	6.1356E-04	1.6495E-05	7.1093E-01	7.6347E-01	6.4047E-06	*4.6069E-12*	*1.0000E-06*

Table S11. Detailed statistical analysis of the results depicted in Figure 6. Adjusted p-values corresponding to all possible pairs are shown below.

No. of 5' adenosines encoded by the template			
	**2A**	**3A**	**4A**
**3A**	1.9208E-03		
**4A**	1.8633E-09	4.7759E-06	
**5A**	3.4732E-11	2.1735E-08	4.0279E-01

Table S12. Guanosine caps at the 5' ends of the pGKL mRNAs are N7-methylated. Tabular representation of the 5' RACE experiments using total RNA prepared from *K. lactis* IFO1267. RNA preparations were analyzed untreated or treated with Rai1 or hDcp2. Results clearly show that pGKL and actin mRNAs were significantly decapped by hDcp2 but were resistant to decapping by Rai1. Numbers represent fractions of capped RNAs from all the independent cDNA clones analyzed. The latter are depicted as numbers in brackets labelled with ^{NAC} (NAC – number of analyzed clones). Numbers labelled with an asterisk are replicated from Table S4.

Analyzed ORF / gene	% of 5' capped RNAs	% of 5' capped RNAs after incubation with Rai1	% of 5' capped RNAs after incubation with hDcp2
K2ORF1	48.7* / (39^{NAC})	40.0 / (15^{NAC})	2.7 / (36^{NAC})
K2ORF2	91.7* / (24^{NAC})	92.3 / (13^{NAC})	0 / (13^{NAC})
actin	100* / (49^{NAC})	100 / (28^{NAC})	0 / (15^{NAC})

Table S13. Summary of 5' RACE experiments of polysomal analyses. Messenger RNAs of each ORF subjected to 5' RACE analysis are characterized by the following data: 1/ ORF name; 2/ yeast strain used for mRNA isolation and cultivation temperature; 3/ source of isolated mRNA; 4/ total number of analyzed clones; 5/ median and mean of added adenosines per mRNA molecule; 6/ median of all 5' adenosines (templated and non-templated) per mRNA molecule; 7/ number of 5' capped mRNAs; 8/ number of 5' polyadenylated mRNAs; 9/ number of 5' polyadenylated and uncapped mRNAs; 10/ number of 5' capped mRNAs and non-polyadenylated mRNAs. In blue are depicted mRNAs purified from yeast strains cultivated at permissive (24°C) temperatures, in red are depicted mRNAs purified from yeast strains cultivated at non-permissive (37°C) temperatures. Total - these numbers are replicated from Table S4; polysomal - high polysome fraction (see Figure 11); unbound - mRNAs not found in polysomes (see Figure 11). Figures 12 and 13 are based on the data shown in this table.

ORF	Yeast strain	RNA	No. of analysed clones	Median / Mean of added adenosines per mRNA		Median of all 5' adenosines (templated and non-templated) per mRNA	No. of 5' capped mRNAs	No. of 5' polyadenylated mRNAs	No. of 5' polyadenylated and uncapped mRNAs	No. of 5' capped mRNAs and non-polyadenylated mRNAs
K1ORF2	*K. lactis* IFO1267 28 °C	total[1]	53	6.0	6.0	10	4	51	48	2
		polysomal	13	8.0	8.0	12	1	13	12	0
	S. cerevisiae CWO4ρ^0*cdc33-42* [pGKL1/2] 24 °C	unbound	20	6.0	5.4	10	2	19	18	0
		polysomal	16	6.0	6.7	10	0	16	16	0
	S. cerevisiae CWO4ρ^0*cdc33-42* [pGKL1/2] 37 °C	unbound	9	9.0	9.4	13	0	9	9	0
		polysomal	15	9.0	9.3	13	1	14	14	1
K2ORF5	*K. lactis* IFO1267 28 °C	total[1]	50	2.0	2.7	5	17	45	30	2
		unbound	10	2.0	2.7	5	4	7	6	3
		polysomal	14	2.0	2.5	5	8	11	6	3
	S. cerevisiae CWO4ρ^0*cdc33-42* [pGKL1/2] 24 °C	unbound	16	2.0	2.3	5	1	12	12	1
		polysomal	26	1.0	1.8	4	15	16	8	8
	S. cerevisiae CWO4ρ^0*cdc33-42* [pGKL1/2] 37 °C	unbound	10	1.0	1.6	4	1	5	5	1
		polysomal	20	1.0	1.4	4	11	10	7	8
K2ORF8	*K. lactis* IFO1267 28 °C	total[1]	79	0	1.0	2	68	36	11	43
	S. cerevisiae CWO4ρ^0*cdc33-42* [pGKL1/2] 24 °C	unbound	9	0	0.6	2	7	3	2	6
		polysomal	10	0	0.3	2	8	2	2	8
	S. cerevisiae CWO4ρ^0*cdc33-42* [pGKL1/2] 37 °C	unbound	8	0	0.5	2	7	3	1	5
		polysomal	ND	N/A	N/A	N/A	N/A	N/A	N/A	N/A
K2ORF10	*K. lactis* IFO1267 28 °C	total[1]	20	4.5	4.8	8	0	18	18	0
	S. cerevisiae CWO4ρ^0*cdc33-42* [pGKL1/2] 24 °C	unbound	21	1.0	2.6	5	3	17	15	1
		polysomal	21	1.0	2.2	5	8	15	10	3
	S. cerevisiae CWO4ρ^0*cdc33-42* [pGKL1/2] 37 °C	unbound	16	1.0	1.8	5	0	12	12	0
		polysomal	23	0	1.8	4	4	12	10	4

[1] from Table S4

SUPPLEMENTARY REFERENCES

Altmann, M., Sonenberg, N., and Trachsel, H. (1989). Translation in Saccharomyces cerevisiae: Initiation Factor 4E-Dependent Cell-Free System. *Molecular and Cellular Biology* 9(10)**,** 4467-4472.

Faul, F., Erdfelder, E., Lang, A.G., and Buchner, A. (2007). G*Power 3: a flexible statistical power analysis program for the social, behavioral, and biomedical sciences. *Behav Res Methods* 39(2)**,** 175-191.

Gueldener, U., Heinisch, J., Koehler, G.J., Voss, D., and Hegemann, J.H. (2002). A second set of loxP marker cassettes for Cre-mediated multiple gene knockouts in budding yeast. *Nucleic Acids Res* 30(6)**,** e23.

Guldener, U., Heck, S., Fielder, T., Beinhauer, J., and Hegemann, J.H. (1996). A new efficient gene disruption cassette for repeated use in budding yeast. *Nucleic Acids Res* 24(13)**,** 2519-2524.

Gunge, N., Tamaru, A., Ozawa, F., and Sakaguchi, K. (1981). Isolation and Characterization of Linear Deoxyribonucleic Acid Plasmids from Kluyveromyces lactis and the Plasmid-Associated Killer Character. *Journal of Bacteriology* 145(1)**,** 382-390.

Gunge, N., and Yamane, C. (1984). Incompatibility of linear DNA killer plasmids pGKL1 and pGKL2 from Kluyveromyces lactis with mitochondrial DNA from Saccharomyces cerevisiae. *J Bacteriol* 159(2)**,** 533-539.

Lin, R.J., Kim, D.H., Castanotto, D., Westaway, S., and Rossi, J.J. (1996). "RNA preparation from yeast cells " in *A Laboratory Guide to RNA: Isolation, Analysis, and Synthesis,* ed. P.A. Krieg. Wiley-Liss, New York), 43-50.

Masek, T., Vopalensky, V., Suchomelova, P., and Pospisek, M. (2005). Denaturing RNA electrophoresis in TAE agarose gels. *Anal Biochem* 336(1)**,** 46-50.

Sýkora, M., Pospíšek, M., Novák, J., Mrvová, S., Krásný, L., and Vopálenský, V. (2018). Transcription apparatus of the yeast virus-like elements: Architecture, function, and evolutionary origin. *PLoS Pathog* 14(10)**,** e1007377. doi: 10.1371/journal.ppat.1007377.

Valis, K., Masek, T., Novotna, D., Pospisek, M., and Janderova, B. (2006). Immunity to killer toxin K1 is connected with the Golgi-to-vacuole protein degradation pathway. *Folia Microbiol (Praha)* 51(3)**,** 196-202.

Woods, D.R., and Bevan, E.A. (1968). Studies on Nature of Killer Factor Produced by Saccharomyces Cerevisiae. *Journal of General Microbiology* 51**,** 115-126. doi: 10.1099/00221287-51-1-115.
